# The Kinetochore-Microtubule Coupling Machinery Is Repurposed in Sensory Nervous System Morphogenesis

**DOI:** 10.1016/j.devcel.2019.02.002

**Published:** 2019-03-25

**Authors:** Dhanya K. Cheerambathur, Bram Prevo, Tiffany-Lynn Chow, Neil Hattersley, Shaohe Wang, Zhiling Zhao, Taekyung Kim, Adina Gerson-Gurwitz, Karen Oegema, Rebecca Green, Arshad Desai

**Affiliations:** 1 Ludwig Institute for Cancer Research, San Diego Branch, La Jolla, CA 92093, USA; 2 Department of Cellular & Molecular Medicine, University of California, San Diego, San Diego, La Jolla, CA 92093, USA

**Keywords:** kinetochore, chromosome segregation, mitosis, microtubule, KMN network, morphogenesis, sensory neuron, nervous system, dendrite, Ndc80 complex, Mis12 complex, Knl1

## Abstract

Dynamic coupling of microtubule ends to kinetochores, built on the centromeres of chromosomes, directs chromosome segregation during cell division. Here, we report that the evolutionarily ancient kinetochore-microtubule coupling machine, the KMN (Knl1/Mis12/Ndc80-complex) network, plays a critical role in neuronal morphogenesis. We show that the KMN network concentrates in microtubule-rich dendrites of developing sensory neurons that collectively extend in a multicellular morphogenetic event that occurs during *C. elegans* embryogenesis. Post-mitotic degradation of KMN components in sensory neurons disrupts dendritic extension, leading to patterning and functional defects in the sensory nervous system. Structure-guided mutations revealed that the molecular interface that couples kinetochores to spindle microtubules also functions in neuronal development. These results identify a cell-division-independent function for the chromosome-segregation machinery and define a microtubule-coupling-dependent event in sensory nervous system morphogenesis.

## Highlights

• Kinetochore-microtubule coupling machinery acts in developing post-mitotic neurons
• Post-mitotic removal of kinetochore components disrupts the sensory nervous system
• Kinetochore components concentrate in sensory dendrites and promote their extension
• The microtubule-coupling interface in developing neurons and mitosis is similar

## Graphical Abstract

**Figure:**
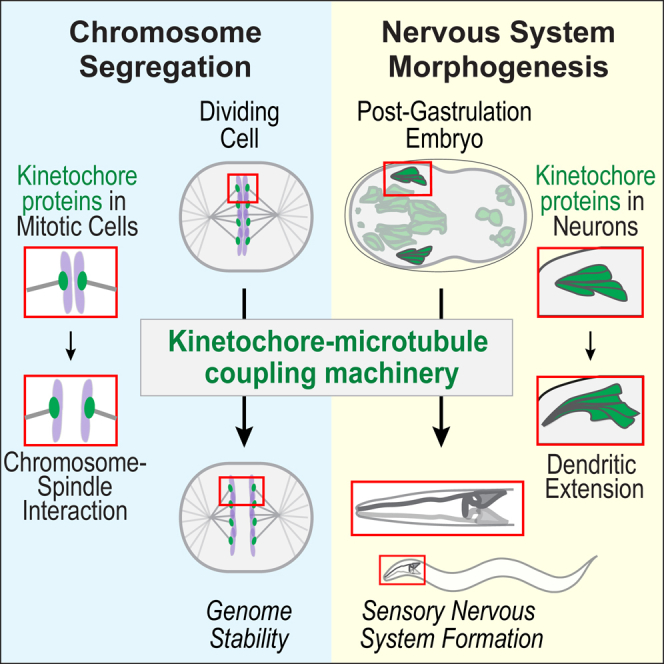

## Teaser

The conserved Knl1/Mis12/Ndc80 (KMN) network constitutes the microtubule-coupling interface at kinetochores in mitosis. Cheerambathur et al. find that the KMN network also acts in the developing sensory nervous system. KMN components concentrate in dendrites of developing neurons and promote their extension, employing the same microtubule-coupling interface that acts in mitosis.

## Introduction

During cell division, the centromere regions of chromosomes assemble large protein machines called kinetochores to connect chromosomes to dynamic spindle microtubules (Cheeseman, 2014, Musacchio and Desai, 2017). At the kinetochore, the conserved 10-subunit Knl1 complex/Mis12 complex/Ndc80 complex (KMN) network is the primary chromosome-microtubule coupler (Cheeseman, 2014, Musacchio and Desai, 2017). The KMN network facilitates chromosome segregation by forming dynamic attachments to ends of polymerizing and depolymerizing spindle microtubules. The KMN network also ensures segregation fidelity by coordinating this mechanical coupling with control of cell cycle progression by acting as a scaffold for checkpoint signaling machinery.

Within the KMN network, the Ndc80 complex forms the primary microtubule coupling module (Cheeseman et al., 2006, Deluca et al., 2006), the Knl1 complex serves as a scaffold for the Ndc80 complex and signaling machinery, and the Mis12 complex links the KMN network to centromeric chromatin (Musacchio and Desai, 2017). At the kinetochore, KMN complexes generate a multivalent high-density microtubule-binding site that couples chromosomes to dynamic microtubule plus ends. Microtubule-coupling activity resides within the Ndc80 subunit of the Ndc80 complex, which harbors two distinct microtubule-interacting elements: a folded calponin homology (CH) domain that recognizes a specific site on the microtubule lattice (Alushin et al., 2010) and a basic unstructured N-terminal tail that provides electrostatic affinity to the negatively charged microtubule surface and mediates cooperative binding along the lattice (Alushin et al., 2012, Ciferri et al., 2008). To date, the sole known function for the specialized microtubule coupling and signaling roles of the KMN network is in chromosome segregation during cell division.

Here, we show that the KMN network serves a critical cell-division-independent function in establishing the proper architecture of the sensory nervous system during *C. elegans* embryogenesis. KMN components concentrate in the microtubule-rich dendrites of developing post-mitotic sensory neurons and their controlled, post-mitotic degradation perturbs initial dendritic extension of sensory neuron bundles, resulting in architectural and functional defects of the sensory nervous system. We additionally demonstrate that the function of the KMN network in sensory nervous system development requires the microtubule-coupling activity resident in the Ndc80 complex. These results identify a new role for the evolutionarily ancient chromosome-segregation machinery and define an early morphogenetic event that establishes the architecture and function of the sensory nervous system.

## Results

### KMN Components Localize to Dendrites of the Developing Sensory Nervous System during Late Embryogenesis

**Figure 1:**
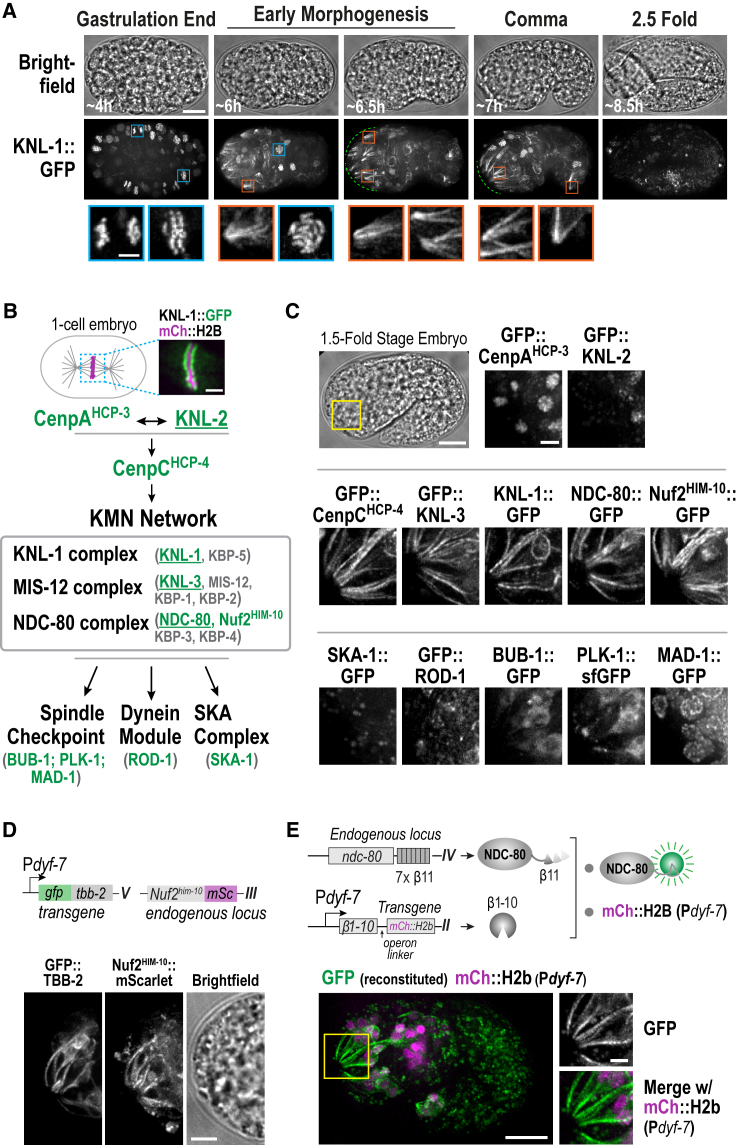
Kinetochore Proteins Concentrate in the Dendritic Extensions of Sensory Neurons (A) Images of *in situ*-tagged KNL-1::GFP at different stages of embryogenesis. KNL-1 localized to chromosomes in dividing cells (blue boxes) and to non-chromosomal structures (orange boxes; dashed green line). Time is relative to first cleavage. Scale bars, 10 μm and 2 μm (insets). (B) Kinetochore assembly in the early *C. elegans* embryo. Proteins labeled in green were *in situ* GFP-tagged for localization analysis; underlined proteins were subjected to functional analysis. Scale bar, 2 μm. (C) Localization of indicated *in situ*-tagged GFP fusions in the anterior of 1.5-fold stage embryos. Scale bars, 10 μm (brightfield) and 2.5 μm (magnified anterior region). (D) Localization of mScarlet-fused Nuf2^HIM-10^ and P*dyf-7*-controlled GFP::β -tubulin (TBB-2). Scale bar, 5 μm. (E) Split GFP analysis with indicated complementing pieces. Scale bars, 10 μm and 2.5 μm (inset).

A hint that the KMN network functions outside of its well-studied cell division context came from analysis of *in situ* GFP-tagged KNL-1 during *C. elegans* embryogenesis. KNL-1 exhibited the expected localization to kinetochores of dividing cells until the end of gastrulation (Sulston et al., 1983) (Figure 1A). However, as the morphogenetic events that structure the tissues and convert the embryonic cell mass into an elongated larva initiated, KNL-1 appeared on cytoplasmic filamentous structures that were prominent in the developing head region in the embryo anterior (Figure 1A). As the embryo began to elongate, the non-chromosomal localization of KNL-1 peaked between “comma” stage and “1.5-fold” stage before declining in intensity and was not observed at later embryonic stages or in L1 larvae (Figure 1A; data not shown). *In situ* GFP-tagging of 12 kinetochore components ranging from constituents of centromeric chromatin (CENPA^HCP-3^ and KNL-2) to the spindle checkpoint (BUB-1 and MAD-1; Figure 1B) revealed that while all components localized to kinetochores in dividing cells, only CENP-C^HCP-4^ and the KMN subunits KNL-3, NDC-80, and Nuf2^HIM-10^ exhibited non-chromosomal localization similar to KNL-1 (Figures 1C and S1A; this localization was also observed for Aurora B^AIR-2^ kinase (Figure S1B), which localizes to chromatin and the spindle midzone in dividing cells (Carmena et al., 2012, Oegema et al., 2001).

The pattern of KMN network localization suggested concentration in the microtubule-rich dendrites of developing sensory neurons (Figures 1D, S1C, and S2A) (Heiman and Shaham, 2009). Imaging of embryos with cell-type-specific promoters driving nuclear and plasma membrane markers indicated that the P*dyf-7* promoter, which is active during early morphogenesis in the majority of sensory neurons in the head (Heiman and Shaham, 2009), best mimicked the spatiotemporal pattern of KMN localization (Figures S1D, S1E, and S2C). To confirm that the non-chromosomal localization of KMN is in developing sensory neurons, we used the split GFP system (Cabantous et al., 2005, Kamiyama et al., 2016). The non-chromosomal localization of NDC-80 in the developing head was reconstituted by expressing complementing elements of GFP from the endogenous *ndc-80* locus and from a P*dyf-7*-controlled transgene (Figures 1E and S2B). These observations suggest a chromosome segregation-independent role for the kinetochore-microtubule coupling machinery in the developing nervous system.

### Post-Mitotic Degradation of KMN Components Perturbs Sensory Nervous System Architecture and Function

**Figure 2:**
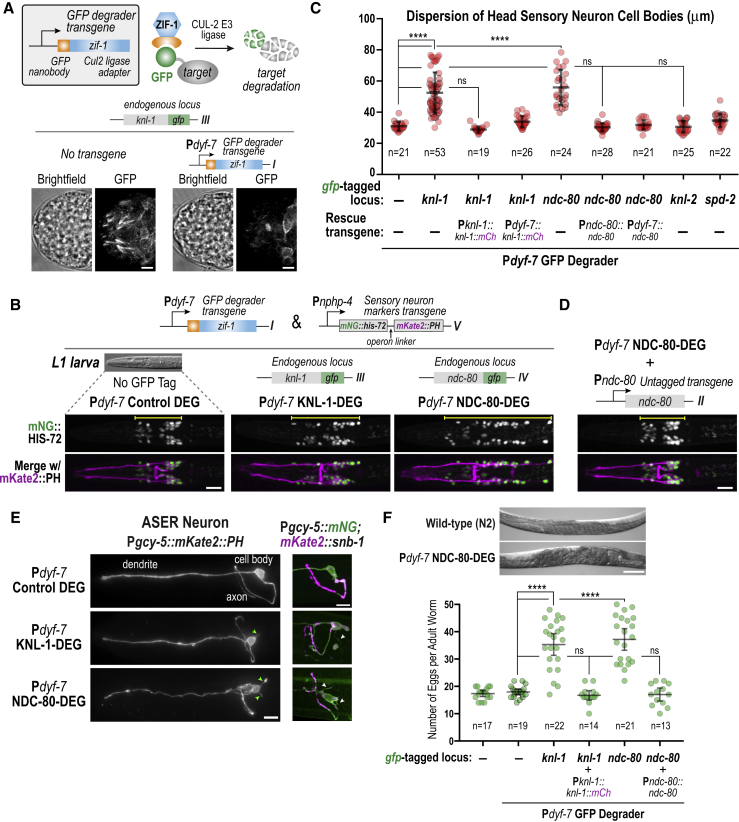
Post-Mitotic Degradation of KMN Proteins Causes Sensory Nervous System Defects (A) Approach used to degrade *in situ* GFP fusions (top). Images below show loss of KNL-1::GFP signal in presence of a P*dyf-7*-controlled GFP degrader. Scale bar, 5 μm. (B) Images of head sensory neuron nuclei and plasma membranes for the indicated conditions. Scale bar, 10 μm. (C) Quantification of dispersion of sensory neuron cell bodies, measured as indicated with yellow lines in (B). Error bars denote the 95% confidence interval. ∗∗∗∗ and ns indicate p < 0.0001 and not significant, respectively. (D) Rescue of sensory nervous system defect by transgene-encoded untagged NDC-80. Scale bar, 10 μm. (E) Profile of ASER neuron (left) and localization of the synaptic marker SNB-1 (right) in the ASER neuron in the indicated conditions. Arrowheads point to ectopic neurites. Scale bar, 10 μm. (F) Egg-laying defect: example image (top) and quantification for indicated conditions (bottom). Scale bar, 100 μm. Error bars denote the 95% confidence interval. ∗∗∗∗ and ns indicate p < 0.0001 and not significant, respectively.

To assess KMN function in developing sensory neurons, we used the P*dyf-7* promoter to express a GFP degrader (Caussinus et al., 2011, Wang et al., 2017) in embryos where *in situ* tagged GFP fusions were the sole source of KNL-1 or NDC-80 (Figure 2A). Imaging of the resulting embryos confirmed loss of the filamentous GFP signal in the head region of the embryo (Figures 2A and S3A). As P*dyf-7* is activated after cell division ceases in the majority of sensory neurons (Heiman and Shaham, 2009), this approach enabled assessment of the post-mitotic role of KMN proteins in sensory nervous system development. Embryos expressing the GFP degrader without an *in situ* GFP-tagged target (P*dyf-7* Control DEG) served as a control. To assess the effect of loss of KNL-1 or NDC-80, we visualized the sensory nervous system in L1 larvae expressing nuclear and plasma membrane markers in ciliated sensory neurons (Figure 2B) (Winkelbauer et al., 2005). In control worms, sensory neuron cell bodies are tightly clustered in a ∼30 μm wide region on either side of the nerve ring. This stereotypical architecture was highly perturbed after degradation of KNL-1 or NDC-80 (Figures 2B and 2C). A similar phenotype was observed following degradation of the KMN component KNL-3 and in a *dyf-7* mutant (Figure S3B) but not with the essential cell division proteins KNL-2 or SPD-2, which are required for kinetochore and centrosome function, respectively (Kemp et al., 2004, Maddox et al., 2007) (Figure 2C); of note, KNL-2 and SPD-2 degradation in the developing intestine revealed mitotic defects similar to KNL-1 or NDC-80 degradation (Figure S3C), indicating that both were susceptible to the action of the GFP degrader. GFP degrader-resistant versions of KNL-1 or NDC-80 expressed under control of endogenous regulatory sequences or P*dyf-7* fully rescued the sensory nervous system architecture defect (Figures 2C and 2D).

To examine the effect of KNL-1 or NDC-80 degradation at a cellular level, we also visualized the morphology of an individual sensory neuron by expressing markers, including the synaptic marker SNB-1, specifically in the ASER neuron (Nonet, 1999). This approach revealed defects in axonal morphology as well as the presence of ectopic neurites along the axon (Figures 2E and S3D). The P*dyf-7*-controlled degradation of KMN components resulted in gross phenotypes associated with impairment of the sensory nervous system, most notably defective egg-laying and reduced fertility (Figures 2F and S3E) (Schafer, 2006). These phenotypes were also rescued by GFP degrader-resistant versions of KNL-1 and NDC-80 (Figures 2F and S3E). Given the severe disorganization of the sensory nervous system, we did not pursue more sophisticated sensory assays (Bargmann et al., 1993, Ward, 1973). We conclude that KMN components have a cell-division-independent function in developing neurons that is required to establish proper sensory nervous system architecture.

### KMN Components Act during the Initial Dendritic Extension of the Amphid Neuron Bundles

**Figure 3:**
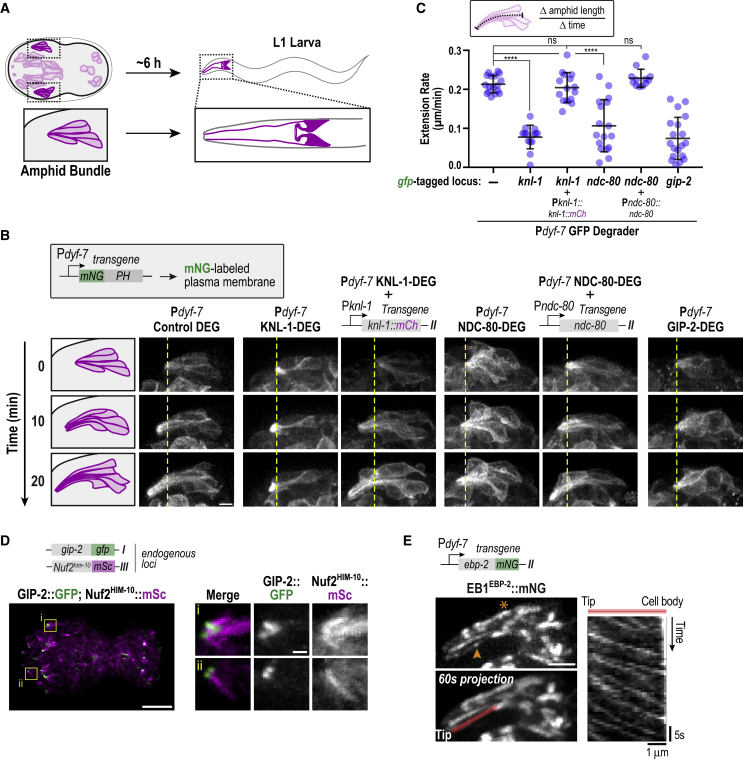
KMN Proteins Are Important for Amphid Bundle Dendrite Extension (A) Schematic relating amphid sensilla in the embryo to their eventual structure in the L1 larva (each sensilla has 12 neurons; only 5 are depicted). (B) Live imaging of amphid bundle dendrite extension for the indicated conditions using a P*dyf-7* controlled plasma membrane marker. Scale bar, 2.5 μm. (C) Quantification of amphid bundle dendrite extension rate. Error bars denote the 95% confidence interval. ∗∗∗∗ and ns indicate p < 0.0001 and not significant, respectively. (D) Color overlay of gamma-tubulin complex (GIP-2::GFP) and KMN protein (Nuf2^HIM-10^::mSc) localization. Magnified insets with individual channel signals are shown on the right. Scale bars, 10 μm (embryo) and 1 μm (inset). Line profile along dendrite of bottom inset is shown on the right. (E) EB1^EBP-2^::mNG dynamics in the amphid bundle. A plus end comet (arrowhead) and a cluster (asterisk) are highlighted in the top still image. Kymograph along the red line in the 60 s time projection image (bottom) is shown on the right. Scale bar, 2.5 μm.

KMN proteins are not present in head sensory neurons after the 2-fold stage in embryogenesis, indicating that the defects observed in L1 larvae are a consequence of KMN inhibition at an earlier developmental stage. To understand how the KMN network contributes to the morphogenesis of the sensory nervous system, we therefore analyzed the effects of post-mitotic KMN inhibition at the time when the localization of KMN components in developing neurons is most prominent (Figure 1A). During the early stages of embryo elongation, two clusters of 12 sensory neurons (called amphid bundles) positioned on either side of the embryo collectively extend microtubule-rich dendritic projections toward the embryo anterior (Figures 3A and 3B) (Heiman and Shaham, 2009). Time-lapse imaging revealed that the rate of amphid bundle dendritic extension toward the embryo anterior was significantly reduced following degradation of KMN proteins (Figures 3B, 3C, and S4A; Video S1); in addition, the organization of the cell bodies within the amphid bundles was abnormal (data not shown). Both phenotypes were rescued by transgenes encoding GFP degrader-resistant KMN components (Figures 3B and 3C; Video S1). Thus, the KMN network is required for a multicellular morphogenetic event during embryogenesis in which bundles of sensory neurons collectively extend microtubule-rich dendritic projections.

Video S1. Amphid Bundle Dendrite Extension in Developing *C. elegans* Embryos, Related to Figure 3B P*dyf-7* Control DEG (left), P*dyf-7* KNL-1-DEG (middle), and P*dyf-7* KNL-1-DEG + P*knl-1* KNL-1::mCherry (right). Time in h:min. Scale bar, 2.5 μm. Images rescaled; 1 pixel true resolution = 2 x 2 pixels in the video.

The established function of the KMN network in microtubule coupling at kinetochores and the presence of microtubules in the extending dendritic projections (Figure 1D) suggested that KMN function in sensory neurons is microtubule related. To test this idea, we first assessed the role of microtubules in the extension of dendritic projections by the amphid bundles by degrading GIP-2, an essential subunit of the microtubule-nucleating γ-tubulin complex (Roostalu and Surrey, 2017, Wang et al., 2015). GIP-2 degradation resulted in a dendrite extension defect similar to KNL-1 or NDC-80 degradation (Figures 3B and 3C), consistent with an essential role for microtubules. We note that following KMN inhibition, sensory neurons in L1 larvae are mispositioned, but dendrites are not substantially reduced in length (Figures 2B and S4B). This is in contrast to the inhibition of GIP-2 (data not shown) or DYF-7, both of which lead to a reduction in dendrite length (Heiman and Shaham, 2009). This comparison suggests that in addition to the KMN-dependent mechanism that positions sensory neurons and promotes dendrite extension, other mechanisms contribute to dendrite extension later in embryogenesis. If the earlier KMN-based mechanism is defective, these later mechanisms still promote dendrite extension but are unable to correct defects in neuronal positioning.

Given the role of the KMN network in coupling to dynamic microtubules at kinetochores, we analyzed the microtubule cytoskeleton within extending amphid bundle dendrites by imaging GIP-2 and the microtubule plus end-tracking protein EB1^EBP-2^. GIP-2 was prominently concentrated in dendrite bundle tips, unlike KMN proteins, which localized along the dendrite length (Figures 3D and S4C). EB1^EBP-2^ comets emerged from the tip region and were primarily directed toward cell bodies (Figures 3E and S4D; Video S2); this organization is consistent with reported predominance in dendrites of microtubules with their minus ends facing out and their plus ends extending toward the cell body (Yan et al., 2013). EB1^EBP-2^ also formed clusters along the length of the amphid bundle (Figure 3E) whose precise nature is unclear; these clusters were non-overlapping with KMN proteins in the extending dendrites (Figure S4E). Following KMN degradation, the shorter dendrites and organizational defects made quantitative comparisons of EB1 dynamics and distribution difficult; however, observed EB1 comets exhibited normal velocity (Figure S4F). Overall, these observations indicate that the KMN network is critical for proper extension of microtubule-rich dendrites and suggest that they provide a microtubule-related function during this early event in the development of sensory neurons.

Video S2. EB1EBP-2::mNG Dynamics in the Amphid Bundle of a Developing *C. elegans* Embryo, Related to Figure 3E Time in min:s. Scale bar, 2.5 μm. Images rescaled; 1 pixel true resolution = 2 x 2 pixels in the video.

### Microtubule Binding Elements of the NDC-80 Complex Are Required for the Function of the KMN Network in Sensory Nervous System Development

**Figure 4:**
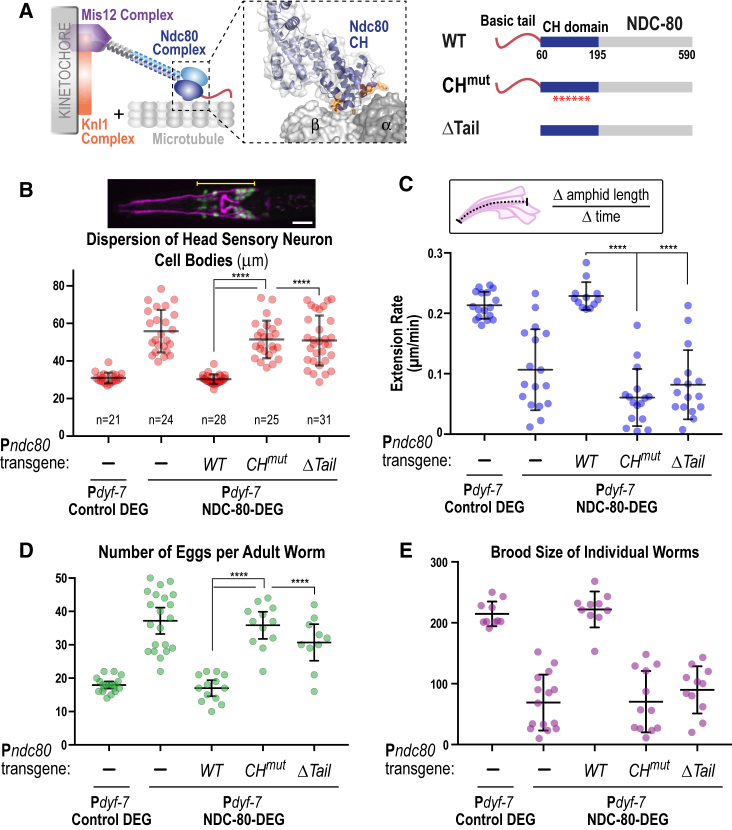
Microtubule-Binding Elements of the KMN Network Are Required for Function in the Developing Sensory Nervous System (A) Schematic highlighting the two microtubule-binding elements of the KMN network: the NDC-80 CH domain that docks onto the microtubule surface and the NDC-80 N-terminal basic tail. Untagged wild-type and mutant NDC-80 variants expressed by single copy transgenes are indicated on the right. (B–E) Phenotypic analysis of transgene-encoded NDC-80 variants following P*dyf*-7-mediated degradation of endogenous GFP-tagged NDC-80. P*dyf*-7 Control DEG, P*dyf*-7 NDC-80 DEG, and P*dyf*-7 NDC-80 DEG plus WT NDC-80 data are the same as in Figures 2C, 2F, and 3C. Scale bar, 10 μm. Error bars denote the 95% confidence interval. ∗∗∗∗ and ns indicate p < 0.0001 and not significant, respectively.

As direct imaging of the microtubule cytoskeleton in amphid bundle dendrites after post-mitotic KNL-1 or NDC-80 degradation proved challenging, we took a different approach to address if the role of the KMN network in sensory nervous system morphogenesis involves its microtubule-coupling activity. For this purpose, we relied on prior studies in chromosome segregation, which established a critical role for microtubule lattice recognition by a conserved set of 6 residues on the surface of NDC-80’s CH domain (Alushin et al., 2010, Ciferri et al., 2008) (Figures 4A and S5A). By expressing degrader-resistant NDC-80 variants at levels equivalent to endogenous NDC-80 (Cheerambathur et al., 2013, 2017) while degrading *in situ* GFP-tagged endogenous NDC-80, we found that the CH domain mutant of NDC-80 fully phenocopied loss of NDC-80 in sensory nervous system architecture (Figures 4B and S5B), amphid bundle dendrite extension (Figures 4C and S5C), egg-laying, and fertility (Figures 4D and 4E). NDC-80 harbors a second *in vitro* microtubule-binding entity, a basic N-terminal tail (Figure 4A) that does not contribute substantially to chromosome segregation in *C. elegans* (Cheerambathur et al., 2017). In contrast to chromosome segregation in early embryos, deletion of the NDC-80 N-terminal tail had an impact comparable to the NDC-80 CH domain mutant in the developing sensory nervous system (Figures 4B–4D and S5B–S5D). As the NDC-80 N-tail is implicated in cooperative binding to the surface of the microtubule (Alushin et al., 2012, Ciferri et al., 2008), this result suggests that N-tail-mediated cooperative microtubule lattice binding may be more critical in the context of KMN network function in the nervous system than in chromosome segregation. We conclude that both microtubule-binding elements of the NDC-80 complex are essential for the post-mitotic function of the KMN network in sensory nervous system development.

We next addressed if disrupting microtubule-binding activity of NDC-80 affects the dendritic localization of other KMN network components. This analysis was challenged by slow red fluorescent protein maturation as well as aggregation of red fluorescent protein fusions; the same targets ( e.g., KNL-1) fused to GFP did not exhibit aggregation. Crossing KNL-1::mCherry into strains where NDC-80 was degraded and replaced with untagged wild-type or CH mutant NDC-80, we found that, even when dendritic extension was impaired, KNL-1::mCherry still localized to dendrites (Figure S5D). With the caveat of the weaker signal due to issues with the fluorophore, this observation suggests that a microtubule binding-independent mechanism analogous to centromeric chromatin in dividing cells may recruit the MIS-12 complex and KNL-1 to the developing dendrites.

## Discussion

Chromosome segregation and neuronal morphogenesis are two processes in which the microtubule cytoskeleton plays an integral role. Here, we show that the KMN network, the ancient microtubule-coupling machine that evolved to harness the intrinsic dynamics of microtubule polymers to drive chromosome segregation, has been repurposed to act during a specific early step in sensory nervous system morphogenesis. Our findings suggest that the microtubule coupling function of the KMN network is required for the initial stages of dendritic extension during embryogenesis, which in turn is critical for establishing the proper architecture and function of the sensory nervous system. While our efforts focused on the sensory nervous system, KMN proteins are also expressed in other neuron types ( e.g., ventral nerve cord precursors; Figure S2B) and extending their analysis to other classes of neurons that form during embryogenesis as well as post-embryonically will be important. In a study in *Drosophila* embryos (Zhao et al., 2019), kinetochore proteins were independently identified as being important for nervous system architecture, suggesting potential conservation of the function we describe here.

Defining the precise spatial relationship between KMN proteins and the microtubule network in dendrites will be important to understand their function in nervous system development. The amphid bundles have dendrites from 12 neurons that are tightly bundled; in addition, these bundles are located at some depth in the embryo (the precise depth varies based on embryo orientation), which causes signal and resolution loss. In addition, we faced unexpected difficulties with the use of red fluorescent proteins at this stage in embryogenesis. mCherry, mKate2, and mScarlet all mature slowly (relative to GFP; in multiple cases, we have the same protein *in situ* tagged with GFP or one or more of the red fluorescent proteins, which allowed directly comparisons) and they formed aggregates; the same red fluorescent protein fusions did not exhibit aggregation in early embryos. While we do not understand why aggregation occurs in later stage embryos, it limited experiments involving red-green fluorescent protein pairs at the stage of embryogenesis when the sensory nervous system is developing. Thus, the precise spatial relationship between KMN proteins and microtubules will need to be defined in the future using better probes and more advanced imaging modalities.

The role of the KMN network in sensory neuronal development during embryogenesis is distinct from well-studied neuronal microtubule-binding proteins that stabilize different classes of microtubules in mature neurons (Kapitein and Hoogenraad, 2015). We show that the neuronal function of the KMN network requires the conserved NDC-80 CH domain-microtubule lattice interaction, which is central to forming a dynamic microtubule end-coupled interface during chromosome segregation. We find that dendritic extension of developing sensory neurons also requires the unstructured basic N-terminal tail of NDC-80, which mediates cooperative binding to microtubules *in vitro* but whose function in chromosome segregation is less clear. We suggest that cooperative binding of KMN complexes along the lattice may be particularly significant in the context of neuronal development. While the mutant analysis establishes the importance of microtubule-coupling elements of NDC-80, their precise contribution during dendritic extension awaits future investigation. Relatively little is known about the initial remodeling of the microtubule cytoskeleton that occurs during dendritic extension in the context of embryogenesis; in the sensory amphid bundles analyzed here, this extension is a multicellular event, the driving force for which is currently not known. The small spatial dimensions involved, together with the tight bundling of dendrites from different neurons in the embryo interior, also limits direct imaging-based analysis of KMN components and microtubules within individual dendrites. While our data are consistent with the idea that the KMN network is utilized to structure and stabilize the microtubule cytoskeleton in developing dendrites, a deeper mechanistic understanding of this process will require the development of a significantly better picture of the complex organizational changes that occur during this event.

Our results show that the KNL-1 and MIS-12 complexes are as important as the NDC-80 complex for sensory nervous system development. During chromosome segregation, the MIS-12 complex and KNL-1 recruit NDC-80 to centromeric chromatin in order to generate a high density of microtubule-binding complexes on the chromosome surface. Within developing neurons, where KMN localization is outside the nucleus, KNL-1 and MIS-12 may still act by recruiting NDC-80 complexes. An important goal of future work will be to define the mechanism(s) that recruit KMN proteins to the dendritic extensions. In addition to its function in NDC-80 recruitment, KNL-1 acts as a scaffold for signaling components that prevent errors during chromosome segregation. It will be intriguing to test if the signaling roles of KNL-1 are also important for the neuronal function of the KMN network.

In addition to defining a new function for ancient chromosome-segregation machinery, our findings suggest new potential reasons for the rapid evolution of kinetochore proteins ( e.g., Knl1 in primates (Genin et al., 2012)) and for the existence of human microcephaly mutations in Knl1 (Genin et al., 2012). We suggest that post-mitotic functions of kinetochore components in nervous system development during embryogenesis, rather than solely in chromosome segregation, may contribute to their rapid evolutionary dynamics and to their association with neurodevelopmental disorders in humans.

## STAR★Methods

### Key Resources Table

**Table:** 

REAGENT or RESOURCE	SOURCE	IDENTIFIER
**Experimental Model: *C. elegans* Organisms/Strains**		

*C. elegans N2 Bristol*	Caenorhabditis Genetics Center	N2
*ltSi1[pOD809/pJE110; Pknl-1::KNL-1reencoded::mCherry; cb-unc-119(+)]II;unc-119(ed3) III*	Espeut et al., 2012, PMID: 22331849	OD334
*ltSi120[[pDC170;Pndc-80:NDC-80 reencoded; cb-unc-119(+)]II; unc-119(ed3)III*	Cheerambathur et al. 2013, PMID: 24231804,	OD611
*ltSi121[pDC175;Pndc-80:NDC-80 (Mutant D1-59) reencoded; cb-unc-119(+)]II; unc-119(ed3)III*	Cheerambathur et al., 2013, PMID: 24231804	OD612
*ltSi272[pOD1272/pSW094; Phlh-1::mCherry::his-72::unc-54_3’UTR; Ppha-4::mCherry::his-72::tbb-2_3’UTR; cb-unc-119(+)]I; unc-119(ed3)III*	This study	OD1006
*ltSi511[pOD1498/pSW207; Pcnd-1::mCherry-PH::unc-54_3'UTR; cb-unc-119(+)]II*	This study	OD1593
*ltSi711[pDC267;Pndc-80:NDC-80(66,96,100,125,144,155AAAAAA) reencoded; cb-unc-119(+)]II#1; unc-119(ed3)III*	Cheerambathur et al., 2017, PMID: 28535376	OD2412
*ska-1(lt28[ska-1::GFP]::loxp)I*	Cheerambathur et al., 2017, PMID: 28535376	OD692
*plk-1(lt18[plk-1::sGFP]::loxp)*	Martino et al., 2017, PMID: 29065307	OD2425
*gip-2(lt19[gip-2::GFP]::loxP::cb-unc-119(+)::loxP)I; unc-119(ed3)III*	Wang et al., 2015, PMID: 26371552	OD2509
*ltSi910[pOD2044/pSW378; Pelt-2::vhhGFP4::ZIF-1::operon-linker::mCherry::histone::tbb-2_3'UTR; cb-unc-119(+)]II; unc-119(ed3)III*	Wang et al., 2015, PMID: 26371552	OD2768
*mdf-1(lt39[GFP::tev::loxP::3xFlag::mdf-1])V*	Wang et al., 2015, PMID: 26371552	OD2906
*him-10(lt52[him-10::GFP]) III*	This Study	OD2953
*ltSi1016[pDC337; Pdyf-7::vhhGFP4::ZIF-1::dyf-7_3'UTR; cb-unc-119(+)]I #2; unc-119(ed3)III*	This Study	OD3025
*knl-1(lt53[knl-1::GFP::tev::loxP::3xFlag]))III*	This Study	OD3026
*ndc-80(lt54[ndc-80::GFP::tev::loxP::3xFlag])IV*	This Study	OD3029
*knl-3 (lt46 [GFP::knl-3]) V*	This Study	OD3101
*ltSi1033 [pDC352; Pdyf-7::mCherry::PH_unc-54_3'UTR; cb-unc-119(+)]II; unc-119(ed3)III*	This Study	OD3152
*ltSi1016[pDC337; Pdyf-7::vhhGFP4::ZIF-1::dyf-7_3'UTR; cb-unc-119(+)]I ] #2; unc-119(ed3)III?; knl-3[lt46(GFP::knl-3)]V*	This Study	OD3174
*air-2(lt58[air-2::GFP::tev::loxP::3xFlag]) I*	This Study	OD3230
*ltSi1038 [pDC344; Pgcy-5::mKate-2::PH_unc-54_3'UTR; cb-unc-119(+)]II; unc-119(ed3)III*	This Study	OD3242
*dyf-7 (lt60)X*	This Study	OD3244
*ltSi1016[pDC337; Pdyf-7::vhhGFP4::ZIF-1::dyf-7_3'UTR; cb-unc-119(+)]I #2; unc-119(ed3)III?; knl-1(lt53[knl-1::GFP::tev::loxP::3xFlag) IV*	This Study	OD3245
*ltSi1016[pDC337; Pdyf-7::vhhGFP4::ZIF-1::dyf-7_3'UTR; cb-unc-119(+)]I #2; ltSi1038 [pDC344; Pgcy-5::mKate-2::PH_unc-54_3'UTR; cb-unc-119(+)]II; unc-119(ed3)III; unc-119(ed3)III?; knl-1(lt53[knl-1::GFP::tev::loxP::3xFlag) IV*	This Study	OD3247
*ltSi1016[pDC337; Pdyf-7::vhhGFP4::ZIF-1::dyf-7_3'UTR; cb-unc-119(+)]I #2; ltSi1038 [pDC344; Pgcy-5::mKate-2::PH_unc-54_3'UTR; cb-unc-119(+)]II; unc-119(ed3)III ?*	This Study	OD3249
*ltSi1041 [pDC376; Pdyf-7::ebp-2::mNeonG_tbb-2_3'UTR::operon-linker::mCherry::PH_unc-54_3'UTR; cb-unc-119(+)]II; unc-119(ed3)III*	This Study	OD3252
*ltSi1016[pDC337; Pdyf-7::vhhGFP4::ZIF-1::dyf-7_3'UTR; cb-unc-119(+)]I ] #2; unc-119(ed3)III? ; ndc-80(lt54[ndc-80::GFP::tev::loxP::3xFlag])IV*	This Study	OD3258
*ltSi1016[pDC337; Pdyf-7::vhhGFP4::ZIF-1::dyf-7_3'UTR]#2 I; ltSi1041 [pDC376; Pdyf-7::ebp-2::mNeonG_tbb-2_3'UTR::operon-linker::mCherry::PH_unc-54_3'UTR; cb-unc-119(+)]II] ; unc-119(ed3)III?*	This Study	OD3275
*ltSi1016[pDC337; Pdyf-7::vhhGFP4::ZIF-1::dyf-7_3'UTR]#2 I; ltSi1041 [pDC376; Pdyf-7::ebp-2::mNeonG_tbb-2_3'UTR::operon-linker::mCherry::PH_unc-54_3'UTR; cb-unc-119(+)]II] ; knl-1(lt53[knl-1::GFP::tev::loxP::3xFlag]) III*	This Study	OD3276
*ltSi1016[pDC337; Pdyf-7::vhhGFP4::ZIF-1::dyf-7_3'UTR; cb-unc-119(+) knl-1(lt53[knl-1::GFP::tev::loxP::3xFlag]) IIIltSi1016[pDC337; Pdyf-7::vhhGFP4::ZIF-1::dyf-7_3'UTR; cb-unc-119(+)]#2 I; ltSi1038 [pDC344; Pgcy-5::mKate-2::PH_unc-54_3'UTR; cb-unc-119(+)]II; unc-119(ed3)III? ; ndc-80(lt54[ndc-80::GFP::tev::loxP::3xFlag])IV*	This Study	OD3281
*ltSi1033 [pDC352; Pdyf-7::mCherry::PH_unc-54_3'UTR; cb-unc-119(+)]II ; unc-119(ed3)III?; knl-3[lt46(GFP::knl-3)]V*	This Study	OD3295
*ltSi1045[pDC378; Pdyf-7::mNeonG::PH_tbb-2_3'UTR::operon-linker::mCherry::his-72_unc-54_3'UTR; cb-unc-119(+)]II; unc-119(ed3)III*	This Study	OD3301
*ltSi1016[pDC337; Pdyf-7::vhhGFP4::ZIF-1::dyf-7_3'UTR; cb-unc-119(+)]I ; ltSi1045[pDC378; Pdyf-7::mNeonG::PH_tbb-2_3'UTR::operon-linker::mCherry::his-72_unc-54_3'UTR; cb-unc-119(+)]II; unc-119(ed3)III?*	This Study	OD3319
*ltSi1016[pDC337; Pdyf-7::vhhGFP4::ZIF-1::dyf-7_3'UTR; cb-unc-119(+)]I ; ltSi1045[pDC378; Pdyf-7::mNeonG::PH_tbb-2_3'UTR::operon-linker::mCherry::his-72_unc-54_3'UTR; cb-unc-119(+)]II; knl-1(lt53[knl-1::GFP::tev::loxP::3xFlag]) III*	This Study	OD3320
*ltSi1016[pDC337; Pdyf-7::vhhGFP4::ZIF-1::dyf-7_3'UTR; cb-unc-119(+)]#2 I] ; ltSi1[pOD809/pJE110; Pknl-1::KNL-1reencoded::mCherry; cb-unc-119(+)]II; knl-1(lt53[knl-1::GFP::tev::loxP::3xFlag]) III*	This Study	OD3346
*ltSi1016[pDC337; Pdyf-7::vhhGFP4::ZIF-1::dyf-7_3'UTR; cb-unc-119(+)]I ; ltSi1045[pDC378; Pdyf-7::mNeonG::PH_tbb-2_3'UTR::operon-linker::mCherry::his-72_unc-54_3'UTR; cb-unc-119(+)]II;unc-119(ed3)III?; ndc-80(lt54[ndc-80::GFP::tev::loxP::3xFlag])IV*	This Study	OD3348
*ltSi1016[pDC337; Pdyf-7::vhhGFP4::ZIF-1::dyf-7_3'UTR; cb-unc-119(+)]I ; ltSi120[[pDC170;Pndc-80:NDC-80 reencoded; cb-unc-119(+)]II #3; unc-119(ed3)III? ; ndc-80(lt54[ndc-80::GFP::tev::loxP::3xFlag])IV*	This Study	OD3355
*ltSi1016[pDC337; Pdyf-7::vhhGFP4::ZIF-1::dyf-7_3'UTR; cb-unc-119(+)]I ; unc-119(ed3)III; ltSi711[pDC267;Pndc-80:NDC-80(66,96,100,125,144,155AAAAAA) reencoded; cb-unc-119(+)]II#1; unc-119(ed3)III? ; ndc-80(lt54[ndc-80::GFP::tev::loxP::3xFlag])IV*	This Study	OD3356
*ltSi1050 [pDC389; Pdyf-7::mCH::his-72_unc-54_3'UTR; cb-unc-119(+)]II; unc-119(ed3)III*	This Study	OD3358
*ltSi1052 [pDC392; Pelt-3::mCH::his-72_unc-54_3'UTR; cb-unc-119(+)]II; unc-119(ed3)III*	This Study	OD3361
*rod-1(lt62[GFP::rod-1) IV*	This Study	OD3367
*ltSi272[pOD1272/pSW094; Phlh-1::mCherry::his-72::unc-54_3’UTR; Ppha-4::mCherry::his-72::tbb-2_3’UTR; cb-unc-119(+)]I; unc-119(ed3)III?; knl-3[lt46(gfp::knl-3)]V*	This Study	OD3372
*ltSi511[pOD1498/pSW207; Pcnd-1::mCherry-PH::unc-54_3'UTR; cb-unc-119(+)]II; unc-119(ed3)III?; knl-3[lt46(GFP::knl-3)]V*	This Study	OD3373
*ltSi1050 [pDC389; Pdyf-7::mCH::his-72_unc-54_3'UTR; cb-unc-119(+)]II; unc-119(ed3)III?; knl-3[lt46(GFP::knl-3)]V*	This Study	OD3398
*ltSi1052 [pDC392; Pelt-3::mCH::his-72_unc-54_3'UTR; cb-unc-119(+)]II; unc-119(ed3)III?; knl-3[lt46(gfp::knl-3)]V*	This Study	OD3399
*knl-1( it75[knl-1::mCherry]) III*	This Study	OD3392
*ltSi1016[pDC337; Pdyf-7::vhhGFP4::ZIF-1::dyf-7_3'UTR; cb-unc-119(+)]I ; ltSi1045[pDC378; Pdyf-7::mNeonG::PH_tbb-2_3'UTR::operon-linker::mCherry::his-72_unc-54_3'UTR; cb-unc-119(+)]II;unc-119(ed3)III?; knl-3[lt46(GFP::knl-3)]V*	This Study	OD3401
*knl-2(lt73[GFP::knl-2])I*	This Study	OD3407
*ltSi1016[pDC337; Pdyf-7::vhhGFP4::ZIF-1::dyf-7_3'UTR; cb-unc-119(+)]I ; ltSi120[[pDC170;Pndc-80:NDC-80 reencoded; cb-unc-119(+)]II #3; unc-119(ed3)III? ; ndc-80(lt54[ndc-80::GFP::tev::loxP::3xFlag])IV; ltSi1054[oxTi365;pDC378; Pdyf-7::mNeonG::PH_tbb-2_3'UTR::operon-linker::mCherry::his-72_unc-54_3'UTR; cb-unc-119(+)] V*	This Study	OD3408
*hcp-4(lt72[GFP::hcp-4])I*	This Study	OD3410
*ltSi1016[pDC337; Pdyf-7::vhhGFP4::ZIF-1::dyf-7_3'UTR; cb-unc-119(+)]I; unc-119(ed3)III; ltSi121[pDC175;Pndc-80:NDC-80 (Mutant D1-59) reencoded; cb-unc-119(+)]II #1; unc-119(ed3)III? ; ndc-80(lt54[ndc-80::GFP::tev::loxP::3xFlag])IV; ltSi1054[oxTi365;pDC378; Pdyf-7::mNeonG::PH_tbb-2_3'UTR::operon-linker::mCherry::his-72_unc-54_3'UTR; cb-unc-119(+)] V*	This Study	OD3412
*ltSi1016[pDC337; Pdyf-7::vhhGFP4::ZIF-1::dyf-7_3'UTR; cb-unc-119(+)]I ; unc-119(ed3)III; ltSi711[pDC267;Pndc-80:NDC-80(66,96,100,125,144,155AAAAAA) reencoded; cb-unc-119(+)]II#1; unc-119(ed3)III? ; ndc-80(lt54[ndc-80::GFP::tev::loxP::3xFlag])IV; ltSi1054[oxTi365;pDC378; Pdyf-7::mNeonG::PH_tbb-2_3'UTR::operon-linker::mCherry::his-72_unc-54_3'UTR; cb-unc-119(+)] V*	This Study	OD3413
*ltSi1016[pDC337; Pdyf-7::vhhGFP4::ZIF-1::dyf-7_3'UTR; cb-unc-119(+)]#2 I] ; ltSi1[pOD809/pJE110; Pknl-1::KNL-1reencoded::RFP; cb-unc-119(+)]II; knl-1(lt53[knl-1::GFP::tev::loxP::3xFlag]) III; ltSi1054[oxTi365;pDC378; Pdyf-7::mNeonG::PH_tbb-2_3'UTR::operon-linker::mCherry::his-72_unc-54_3'UTR; cb-unc-119(+)] V*	This Study	OD3431
*spd-2(lt76[gfp::spd-2]) I*	This Study	OD3453
*hcp-3(lt78[GFP::hcp-3])I*	This Study	OD3463
*bub-1(lt82 [bub-1::GFP])I*	This Study	OD3516
*ltSi1168[pDC589;Pdyf-7::ndc-80 reencoded-::mCherry::dyf_3'UTR; cb-unc-119(+)]II; unc-119(ed3)III*	This Study	OD3915
*unc-119(ed3)III; ltSi1174[oxTi365; pDC591; Pnphp-4::mNeonGreen-his-72:tbb-2_3'UTR;;gpd-2/3 operon linker-mKate2-PH:unc-34_3'UTR]V*	This Study	OD3919
*ltSi1169[pDC588;Pdyf-7::knl-1 reencoded-::mCherry::dyf_3'UTR; cb-unc-119(+)]II; unc-119(ed3)III*	This Study	OD3920
*ltSi1175[pDC585; Pcnd-1::mCH::his-11_unc-54_3'UTR; cb-unc-119(+)]II; unc-119(ed3)III*	This Study	OD3921
*ltSi1016[pDC337; Pdyf-7::vhhGFP4::ZIF-1::dyf-7_3'UTR; cb-unc-119(+)]I; unc-119(ed3)III?; ltSi1174[oxTi365; pDC591; Pnphp-4::mNeonGreen-his-72:tbb-2_3'UTR;;gpd-2/3 operon linker-mKate2-PH:unc-34_3'UTR]V*	This Study	OD3924
*ltSi1175[pDC585; Pcnd-1::mCH::his-11_unc-54_3'UTR; cb-unc-119(+)]II; unc-119(ed3)III?; knl-3[lt46(gfp::knl-3)]V*	This Study	OD3927
*ltSi1016[pDC337; Pdyf-7::vhhGFP4::ZIF-1::dyf-7_3'UTR; cb-unc-119(+)] I] ; knl-1(lt53[knl-1::GFP::tev::loxP::3xFlag]) III; ltSi1174[oxTi365; pDC591; Pnphp-4::mNeonGreen-his-72:tbb-2_3'UTR;;gpd-2/3 operon linker-mKate2-PH:unc-34_3'UTR]V*	This tudy	OD3938
*ltSi910[pOD2044/pSW378; Pelt-2::vhhGFP4::ZIF-1::operon-linker::mCherry::histone::tbb-2_3'UTR; cb-unc-119(+)]II;knl-1(lt53[knl-1::GFP::tev::loxP::3xFlag])III*	This Study	OD3931
*ltSi910[pOD2044/pSW378; Pelt-2::vhhGFP4::ZIF-1::operon-linker::mCherry::histone::tbb-2_3'UTR; cb-unc-119(+)]II; unc-119(ed3)III?;ndc-80(lt54[ndc-80::GFP::tev::loxP::3xFlag])IV*	This Study	OD3932
*ltSi910[pOD2044/pSW378; Pelt-2::vhhGFP4::ZIF-1::operon-linker::mCherry::histone::tbb-2_3'UTR; cb-unc-119(+)]II; unc-119(ed3)III?;knl-3 (lt46 [GFP::knl-3]) V*	This Study	OD3933
*spd-2(lt76[gfp::spd-2])I; ltSi910[pOD2044/pSW378; Pelt-2::vhhGFP4::ZIF-1::operon-linker::mCherry::histone::tbb-2_3'UTR; cb-unc-119(+)]II; unc-119(ed3)III*	This Study	OD3934
*knl-2(lt73[gfp::knl-2])I; ltSi910[pOD2044/pSW378; Pelt-2::vhhGFP4::ZIF-1::operon-linker::mCherry::histone::tbb-2_3'UTR; cb-unc-119(+)]II; unc-119(ed3)III?*	This Study	OD3936
*ltSi1016[pDC337; Pdyf-7::vhhGFP4::ZIF-1::dyf-7_3'UTR; cb-unc-119(+)]I ; unc-119(ed3)III? ;Pnphp-4::mNeonGreen-his-72:tbb-2_3'UTR;;gpd-2/3 operon linker-mKate2-PH:unc-34_3'UTR] knl-3[lt46(GFP::knl-3)]V*	This Study	OD3948
*ltSi1016[pDC337; Pdyf-7::vhhGFP4::ZIF-1::dyf-7_3'UTR; cb-unc-119(+)] gip-2(lt19[gip-2::GFP]::loxP::cb-unc-119(+)::loxP)I ; ltSi1045[pDC378; Pdyf-7::mNeonG::PH_tbb-2_3'UTR::operon-linker::mCherry::his-72_unc-54_3'UTR; cb-unc-119(+)]II;unc-119(ed3)III?*	This Study	OD3951
*ltSi1016[pDC337; Pdyf-7::vhhGFP4::ZIF-1::dyf-7_3'UTR; cb-unc-119(+)]I ; ltSi120[[pDC170;Pndc-80:NDC-80 reencoded; cb-unc-119(+)]II #3; unc-119(ed3)III? ; ndc-80(lt54[ndc-80::GFP::tev::loxP::3xFlag])IV; Pnphp-4::mNeonGreen-his-72:tbb-2_3'UTR;;gpd-2/3 operon linker-mKate2-PH:unc-34_3'UTR]V*	This Study	OD3952
*ltSi1016[pDC337; Pdyf-7::vhhGFP4::ZIF-1::dyf-7_3'UTR; cb-unc-119(+)]I ; unc-119(ed3)III; ltSi121[pDC175;Pndc-80:NDC-80 (Mutant D1-59) reencoded; cb-unc-119(+)]II #1; unc-119(ed3)III? ; ndc-80(lt54[ndc-80::GFP::tev::loxP::3xFlag])IV; Pnphp-4::mNeonGreen-his-72:tbb-2_3'UTR;;gpd-2/3 operon linker-mKate2-PH:unc-34_3'UTR]V*	This Study	OD3953
*ltSi1016[pDC337; Pdyf-7::vhhGFP4::ZIF-1::dyf-7_3'UTR; cb-unc-119(+)]I ; unc-119(ed3)III; ltSi711[pDC267;Pndc-80:NDC-80(66,96,100,125,144,155AAAAAA) reencoded; cb-unc-119(+)]II#1; unc-119(ed3)III? ; ndc-80(lt54[ndc-80::GFP::tev::loxP::3xFlag])IV; ltSi1055[oxTi365;[pDC344; Pnphp-4::mNeonGreen-his-72:tbb-2_3'UTR;;gpd-2/3 operon linker-mKate2-PH:unc-34_3'UTR]V*	This Study	OD3954
*ltSi1016[pDC337; Pdyf-7::vhhGFP4::ZIF-1::dyf-7_3'UTR; cb-unc-119(+)]I; unc-119(ed3)III?; ltSi1174[oxTi365; pDC591; Pnphp-4::mNeonGreen-his-72:tbb-2_3'UTR;;gpd-2/3 operon linker-mKate2-PH:unc-34_3'UTR]V; dyf-7 (it60)X*	This Study	OD3965
*ltSi1016[pDC337; Pdyf-7::vhhGFP4::ZIF-1::dyf-7_3'UTR; cb-unc-119(+)] knl-2(lt73[GFP::knl-2])I; unc-119(ed3)III?; ltSi1174[oxTi365; pDC591; Pnphp-4::mNeonGreen-his-72:tbb-2_3'UTR;;gpd-2/3 operon linker-mKate2-PH:unc-34_3'UTR]V*	This Study	OD3971
*unc-119(ed3) III; ltSi1181[oxTi365; pDC559; Pdyf-7:GFP-tbb-2_dyf-7_3'UTR; cb-unc-119(+)]V*	This Study	OD3974
*ltSi1184[pDC593; Pdyf-7::sGFPS1-10::tbb-2_3'UTR::gpd-2/3 operon linker::mCherry-his-15::unc-54-3'UTR;; cb-unc-119(+)] II; unc-119(ed3) III*	This Study	OD3976
*ltSi1016[pDC337; Pdyf-7::vhhGFP4::ZIF-1::dyf-7_3'UTR; cb-unc-119(+)] I] ; ltSi1[pOD809/pJE110; Pknl-1::KNL-1reencoded::RFP; cb-unc-119(+)]II; knl-1(lt53[knl-1::GFP::tev::loxP::3xFlag]) III; ltSi1174[oxTi365; pDC591; Pnphp-4::mNeonGreen-his-72:tbb-2_3'UTR;;gpd-2/3 operon linker-mKate2-PH:unc-34_3'UTR]V*	This Study	OD3977
*ndc-80[lt126 (ndc-80::7XGFP-11)] IV*	This Study	OD3995
*ltSi1184[pDC593; Pdyf-7::sGFPS1-10::tbb-2_3'UTR::gpd-2/3 operon linker::mCherry-his-15::unc-54-3'UTR;; cb-unc-119(+)] II; unc-119(ed3) III?; ndc-80[lt126 (ndc-80::7XGFP-11)] IV*	This Study	OD3997
*ltSi1016[pDC337; Pdyf-7::vhhGFP4::ZIF-1::dyf-7_3'UTR; cb-unc-119(+)]I ; unc-119(ed3)III? ; ndc-80(lt54[ndc-80::GFP::tev::loxP::3xFlag])IV; ltSi1174[oxTi365; pDC591; Pnphp-4::mNeonGreen-his-72:tbb-2_3'UTR;;gpd-2/3 operon linker-mKate2-PH:unc-34_3'UTR]V*	This Study	OD3998
*ltSi1191[pDC603; Pgcy-5::mNeonGreen-PH::tbb-3'UTR; Pgcy-5::mKate2-snb-1::snb-1_3'UTR; cb-unc-119(+)] II; unc-119(ed3) III*	This Study	OD4000
*ltSi1016[pDC337; Pdyf-7::vhhGFP4::ZIF-1::dyf-7_3'UTR; cb-unc-119(+)]I ;ltSi1191[pDC603; Pgcy-5::mNeonGreen-PH::tbb-3'UTR; Pgcy-5::mKate2-snb-1::snb-1_3'UTR; cb-unc-119(+)] II; unc-119(ed3) III?*	This Study	OD4011
*ltSi1016[pDC337; Pdyf-7::vhhGFP4::ZIF-1::dyf-7_3'UTR; cb-unc-119(+)] I] ;ltSi1191[pDC603; Pgcy-5::mNeonGreen-PH::tbb-3'UTR; Pgcy-5::mKate2-snb-1::snb-1_3'UTR; cb-unc-119(+)] II; knl-1(lt53[knl-1::GFP::tev::loxP::3xFlag]) III*	This Study	OD4012
*ltSi1016[pDC337; Pdyf-7::vhhGFP4::ZIF-1::dyf-7_3'UTR; cb-unc-119(+)]I ; unc-119(ed3)III? ;ltSi1191[pDC603; Pgcy-5::mNeonGreen-PH::tbb-3'UTR; Pgcy-5::mKate2-snb-1::snb-1_3'UTR; cb-unc-119(+)] II; unc-119(ed3) III?; ndc-80(lt54[ndc-80::GFP::tev::loxP::3xFlag])IV*	This Study	OD4013
*ltSi1016[pDC337; Pdyf-7::vhhGFP4::ZIF-1::dyf-7_3'UTR; cb-unc-119(+)] spd-2(lt76[GFP::spd-2]) I; unc-119(ed3)III?; ltSi1174[oxTi365; pDC591; Pnphp-4::mNeonGreen-his-72:tbb-2_3'UTR;;gpd-2/3 operon linker-mKate2-PH:unc-34_3'UTR]V*	This Study	OD4014
*ltSi1186[ pDC599; Pcnd-1::sGFPS1-10::tbb-2_3'UTR::gpd-2/3 operon linker::mCherry-his-15::unc-54-3'UTR;; cb-unc-119(+)] II; unc-119(ed3) III*	This Study	OD4015
*ltSi1016[pDC337; Pdyf-7::vhhGFP4::ZIF-1::dyf-7_3'UTR; cb-unc-119(+)] I]; ltSi1169[pDC588;Pdyf-7::knl-1 reencoded-::mCherry::dyf_3'UTR; cb-unc-119(+)]II; knl-1(lt53[knl-1::GFP::tev::loxP::3xFlag]) III; ltSi1174[oxTi365; pDC591; Pnphp-4::mNeonGreen-his-72:tbb-2_3'UTR;;gpd-2/3 operon linker-mKate2-PH:unc-34_3'UTR]V*	This Study	OD4025
*ltSi1016[pDC337; Pdyf-7::vhhGFP4::ZIF-1::dyf-7_3'UTR; cb-unc-119(+)]I ; ltSi1168[pDC589;Pdyf-7::ndc-80 reencoded-::mCherry::dyf_3'UTR; cb-unc-119(+)]II; unc-119(ed3)III? ; ndc-80(lt54[ndc-80::GFP::tev::loxP::3xFlag])IV; ltSi1174[oxTi365; pDC591; Pnphp-4::mNeonGreen-his-72:tbb-2_3'UTR;;gpd-2/3 operon linker-mKate2-PH:unc-34_3'UTR]V*	This Study	OD4026
*him-10[lt130(him-10::mScarlet)] III; unc-119(ed3) III?; ltSi1181[oxTi365; pDC559; Pdyf-7:GFP-tbb-2_dyf-7_3'UTR; cb-unc-119(+)]V*	This Study	OD4038
*ltSi1186[ pDC599; Pcnd-1::sGFPS1-10::tbb-2_3'UTR::gpd-2/3 operon linker::mCherry-his-15::unc-54-3'UTR;; cb-unc-119(+)] II; unc-119(ed3) III?; ndc-80[lt126 (ndc-80::7XGFP-11)] IV*	This Study	OD4039
*him-10[lt130(him-10::mScarlet)] III*	This Study	OD4040
*gip-2(lt19[gip-2::GFP]::loxP::cb-unc-119(+)::loxP)I; unc-119(ed3)III?; him-10[lt130(him-10::mScarlet)] III*	This Study	OD4107
*ltSi1041 [pDC376; Pdyf-7::ebp-2::mNeonG_tbb-2_3'UTR::operon-linker::mCherry::PH_unc-54_3'UTR; cb-unc-119(+)]II ; unc-119(ed3)III?; him-10[lt130(him-10::mScarlet)] III*	This Study	OD4109
*ltSi1016[pDC337; Pdyf-7::vhhGFP4::ZIF-1::dyf-7_3'UTR; cb-unc-119(+)]I ]; knl-1( it75[knl-1::mCherry]) III*	This Study	DKC05
*ltSi1016[pDC337; Pdyf-7::vhhGFP4::ZIF-1::dyf-7_3'UTR; cb-unc-119(+)]I ]; knl-1( it75[knl-1::mCherry]) III;ndc-80(lt54[ndc-80::GFP::tev::loxP::3xFlag])IV*	This Study	DKC06
*ltSi1016[pDC337; Pdyf-7::vhhGFP4::ZIF-1::dyf-7_3'UTR; cb-unc-119(+)]I ]; ltSi120[[pDC170;Pndc-80:NDC-80 reencoded; cb-unc-119(+)]II #3; knl-1( it75[knl-1::mCherry]) III;ndc-80(lt54[ndc-80::GFP::tev::loxP::3xFlag])IV*	This Study	DKC07
*ltSi1016[pDC337; Pdyf-7::vhhGFP4::ZIF-1::dyf-7_3'UTR; cb-unc-119(+)]I ]; ltSi711[pDC267;Pndc-80:NDC-80(66,96,100,125,144,155AAAAAA) reencoded; cb-unc-119(+)]II#1; knl-1( it75[knl-1::mCherry]) III;ndc-80(lt54[ndc-80::GFP::tev::loxP::3xFlag])IV*	This Study	DKC08

**Software and Algorithms**		

Image J (Fiji)	N/A	http://rsbweb.nih.gov/ij/
GraphPad Prism	N/A	http://www.graphpad.com/
Pymol 1.8	N/A	https://www.pymol.org

**Other**		

Andor Technology System – Yokogawa Spinning Disk Unit	Andor / Nikon	N/A
Deltavision Elite – Applied Precision / PCO	GE Healthcare / Olympus	N/A
Zeiss LSM 880 AxioObserver System	Zeiss	N/A
Zeiss Z1 AxioObserver System – Yokogawa Spinning Disk Unit	Zeiss / Photometrics	N/A

### Contact for Reagent and Resource Sharing

Further information and requests regarding reagents should be directed to and will be fulfilled by the Lead Contact, Arshad Desai (abdesai@ucsd.edu).

### Experimental Model and Subject Details

All *C. elegans* strains were maintained at 20°C on standard Nematode Growth Media (NGM) plates seeded with OP50 bacteria. The genotypes of the C. elegans strains used in this study are described in *Reagents and Resources*.

### Method Details

#### *C. elegans* Transgenic Strain Construction

Single copy transgenic integrations were engineered using the transposon based mos1 mediated Single Copy Insertion (mosSCI) method (Frøkjaer-Jensen et al., 2008). Briefly, the strains were generated by injecting a mixture of repair plasmid containing the transgene and a positive selection marker, transposase plasmid, and the three plasmids encoding fluorescent markers for negative selection (Frøkjaer-Jensen et al., 2008) [pCFJ90 (P*myo-2::mCherry*), pCFJ104 (P*myo-3::mCherry*) and pGH8 (P*rab-3::mCherry*)] into appropriate *C. elegans* strains that contain mos1 insertion sites at specific genomic locations within Chr I, II or V. Positive integrants were identified by selecting worms that were moving and did not contain fluorescent selection markers. Integration of transgenes were confirmed by PCR, spanning both homology arms. Endogenous tagging of various genes (see Table S1) at the N- or C-teminus with GFP or mScarlet and generation of the *dyf-7* deletion were done using CRISPR/Cas9 methods (Dickinson et al., 2015, Paix et al., 2015, Waaijers et al., 2013). The specific method and guide RNAs used to generate each strain are described in Table S1. Briefly, for the generation of GFP or mScarlet fusions, the repair template (consisting of ∼1500bp flanking either GFP or mScarlet) was co-injected into wildtype N2 animals with plasmids containing Cas9 (pDD162 (Dickinson et al., 2013)), the respective guide RNA sequences, and the three plasmids encoding fluorescent markers for negative selection (Frøkjaer-Jensen et al., 2008) [pCFJ90 (P*myo-2::mCherry*), pCFJ104 (P*myo-3::mCherry*) and pGH8 (P*rab-3::mCherry*)]. Recombinant strains were identified by appropriate selection method and were confirmed by PCR, spanning both homology regions.

#### Plasmid Construction

All plasmids were constructed using the Gibson Assembly method (Gibson et al., 2009). The cloning strategy for the GFP nanobody fragment (vhhGFP4) fused to ZIF-1 genomic DNA, and the regulatory elements for *ndc-80* and *knl-1* single copy transgene insertion, under endogenous promoter, have been previously described (Cheerambathur et al., 2013, Espeut et al., 2012, Wang et al., 2017). The regulatory sequences used for the generation of all other strains, and the codon-optimized sequences for Split GFP and mScarlet are in Table S2.

#### Fluorescence Microscopy and Image Analysis

Live embryo imaging experiments were performed using an Andor Technology confocal imaging system (described below), unless noted otherwise. The Andor Revolution XD Confocal System was equipped with a Yokogawa spinning disk unit (CSU-10, Yokogawa Corporation of America) that was mounted on a Nikon inverted microscope (TE2000-E, Nikon) housing a 100X 1.4 NA Plan Apochromat (Nikon) objective. Two solid-state 100 mW lasers provided illumination and a back-illuminated EMCCD camera (iXon DV887; Andor Technology) was used for detection. Embryos in Figure S5D were imaged using a Zeiss LSM 880 AxioObserver with a 1.46 NA 100X Alpha Plan Apochromat (Zeiss) objective. A 561 nm HeNe laser provided illumination and emission light was detected using a GaAsp detector. 8 x 0.5 μm z-stacks, containing 904 pixel by 904 pixel images having a spatial resolution of 94 nm/pixel, were acquired and projected. For imaging, embryos were mounted in M9 on a 2% agarose pad, which was subsequently covered with a 22 x 22 mm coverslip and sealed with VaLaP (1:1:1 vasoline:lanolin:paraffin). Z-stacks were acquired to cover approximately half the embryo depth from the side facing the coverslip / objective.

To quantify neurite extension, 11 x 0.5 μm z-stacks were acquired at 1 min intervals and 100 ms exposure. Z-stacks were projected and bundle extension was measured using a segmented line (spline fit) drawn over the top of the amphid bundle extending from the tip to a reference point at the back of the cellular complex using Image J (Fiji).

To quantify protein degradation, 21 x 0.5 μm z-stacks were acquired at an exposure of 100 ms (for OD3029 and OD3258, see Key Resources Table) or at 200 ms (for OD3026 and OD3245, see Key Resources Table). Z-stacks were projected and the integrated fluorescence intensity was measured in the anterior part of the head (comma stage embryos), using Image J (Fiji), while applying an intensity threshold of 10000 and excluding any signal arising from kinetochore localization.

To image EBP-2 dynamics, 5 x 0.75 μm z-stacks were acquired at 0.8 sec intervals and 50 ms exposure. Z-stacks were projected and kymographs were generated using the KymographDirect (Mangeol et al., 2016) Image J/Fiji plugin. EBP-2 velocities were extracted from the kymographs manually.

To image sensory neurons expressing P*nphp-4* driven mKate-PH and mNeonGreen histone, L1 stage worms were anesthetized in 5mM Levamisole and mounted in M9 on a 2% agarose pad. 30 x 0.5 μm z-stacks were acquired using an inverted Zeiss Axio Observer Z1 system with a Yokogawa spinning-disk confocal head (CSU-X1), a 63X 1.4 NA Plan Apochromat objective (Zeiss, Oberkochen, Germany), and an EMCCD camera (QuantEM:512SC, Photometrics, Tucson, AZ). Maximum intensity projections of z-stacks were made using Image J (Fiji) and the cell body distribution width was measured as the distance between the most anterior and most posterior cell body within the head region. To image the ASER neuron expressing mKate-PH, synchronized adult worms were anesthetized in 5 mM Levamisole and mounted in M9 on a 2% agarose pad. 60 x 0.5 μm z-stacks were acquired using a deconvolution microscope (Deltavision Elite; Applied Precision) equipped with a CMOS camera (pco.edge 5.5 sCMOS; PCO) and a 60X 1.42NA PlanApo N objective (Olympus). The z-stacks were deconvolved and maximum intensity projections were made using softWorRx (Applied Precision).

To image the ASER neuron expressing mNeonGreen and mKate-SNB-1 markers, synchronized adult worms were anesthetized as described above. 60 x 0.5 μm z-stacks were acquired using an inverted Zeiss Axio Observer Z1 system with a Yokogawa spinning-disk confocal head (CSU-X1), a 63X 1.40 NA Plan Apochromat objective (Zeiss, Oberkochen, Germany), and an EMCCD camera (QuantEM:512SC, Photometrics, Tucson, AZ). Maximum intensity projections of z-stacks were made using Image J (Fiji).

#### Assay for Egg-Laying Defect

The number of unlaid eggs was determined as described previously (Koelle and Horvitz, 1996). Briefly, late stage L4s were collected and grown at 20°C for 36 hours. Each adult worm was bleached in 2 μl 50% sodium hypochlorite solution to dissolve the mother. Subsequently, the embryos, which are protected from the bleach by their eggshell, were counted.

#### Fertility Assay

L4 worms were singled onto NGM plates and transferred to a fresh plate two additional times, every 24 hours. The progeny on each plate was counted 24 hours after the transfer and summed to give the brood size.

#### Dye-Fill Assay

Dye-fill assays were carried out by incubating 100 worms in a 1/500 dilution of DiI (Invitrogen) in M9 buffer for 1 hr. The worms were washed with M9, transferred on to a seeded NGM plate for 30 min to destain, anesthetized in 5mM Levamisole and mounted on a 2% agarose pad as described above. 60 x 0.5 μm z-stacks were acquired on the Andor system (described above) with a 40X 0.75 NA Plan Fluor objective (Nikon). Maximum intensity projections of z-stacks were made using Image J (Fiji).

### Quantification and Statistical Analysis

Details of the methods employed to extract and quantify various parameters in microscopy datasets are described in the image analysis section. The statistical tests used to determine significance are described in the figure legends. Pairwise comparisons were done using 2-tailed unpaired t-tests in GraphPad Prism (GraphPad Software) and the stars ^∗∗∗∗^, ^∗∗∗^, ^∗∗^ and ns correspond p<0.0001, p<0.001, p<0.01 and “not significant”, respectively.

## Supplemental Information

Document S1. Figures S1–S5 and Tables S1 and S2

Document S2. Article plus Supplemental Information
