# Supplementary material for: The Kinetochore-Microtubule Coupling Machinery Is Repurposed in Sensory Nervous System Morphogenesis
Source: Dev Cell. 2019 Mar 25;48(6):864–872.e7. doi: 10.1016/j.devcel.2019.02.002 (PMC6436928; doi:10.1016/j.devcel.2019.02.002)
Supplement: Document S1. Figures S1–S5 and Tables S1 and S2 [file mmc1.pdf]

Developmental Cell, Volume *48*

Supplemental Information

The Kinetochore-Microtubule Coupling Machinery Is Repurposed in Sensory Nervous System Morphogenesis

Dhanya K. Cheerambathur, Bram Prevo, Tiffany-Lynn Chow, Neil Hattersley, Shaohe Wang, Zhiling Zhao, Taekyung Kim, Adina Gerson-Gurwitz, Karen Oegema, Rebecca Green, and Arshad Desai

Figure. S1

Cheerambathur, Prevo et al.

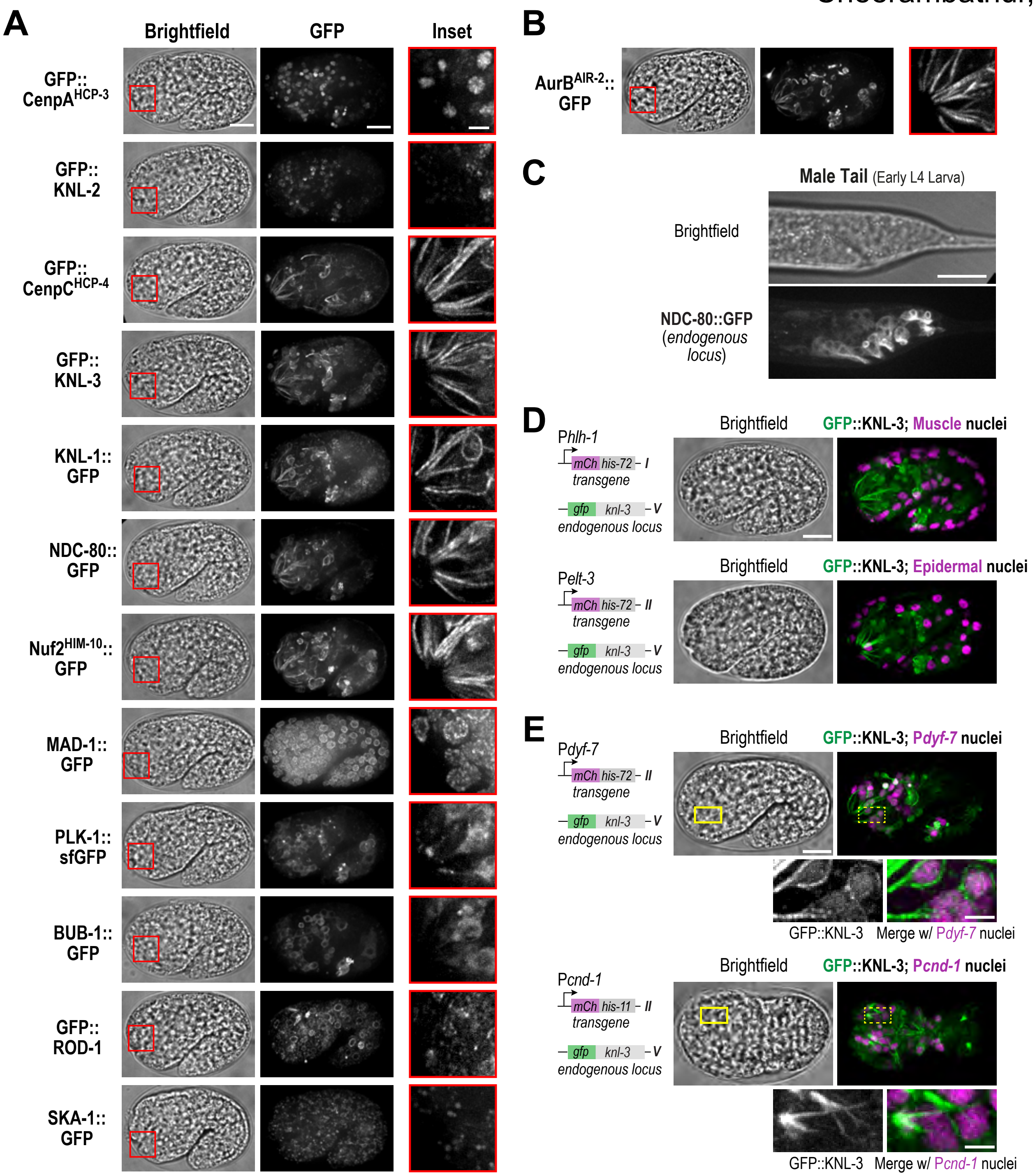

Figure. S1. Localization of kinetochore components and Aurora B^{AIR-2}, NDC-80 localization in the larval male tail, and KNL-3 localization in embryos expressing muscle, epidermal and neuronal nuclear markers (Related to Figure 1). (A) Images of full embryos and their anterior developing head regions (red box in brightfield panels) for the indicated *in situ* GFP fusions. Insets are the same as those shown in Figure 1B. Scale bars 10 µm in full embryos; 2.5 µm in inset. **(B)** Similar scale images as (A) for *in situ* GFP-tagged Aurora B^{AIR-2} kinase. **(C)** NDC-80::GFP localization in the male tail (early L4 larval stage). NDC-80 concentrates in the neurites of post-embryonic developing sensory neurons. Scale bar, 20 µm. **(D)** GFP::KNL-3 localization in embryos with single copy transgene insertions expressing nuclear-localized mCherry-tagged histone H3.3 (HIS-72) under control of muscle-specific (*hlh-1)* or epidermis-specific (*elt-3)* promoters (Krause et al.,1990; Gilleard et al.,1999) Scale bar 10 µm. **(E)** GFP::KNL-3 localization in embryos with single copy transgene insertions expressing nuclear-localized mCherry-tagged histone H3.3 (HIS-72) under control of the *dyf-7* promoter or histone H2B (HIS-11) under control of the *cnd-1* promoter. Region highlighted with yellow boxes is magnified below. The promoters P*dyf-7* and P*cnd-1* are transiently active in developing neurons. P*dyf-7* is activated at the time of early morphogenesis (Heiman et al., 2009) and P*cnd-1* is activated early in development (Hallam et al., 2000). The embryo stage shown is soon after morphogenesis initiates(see also Figure S2A). Scale bars: 10 µm for full embryo and 2.5 µm for insets.

Figure. S2

Cheerambathur, Prevo et al.

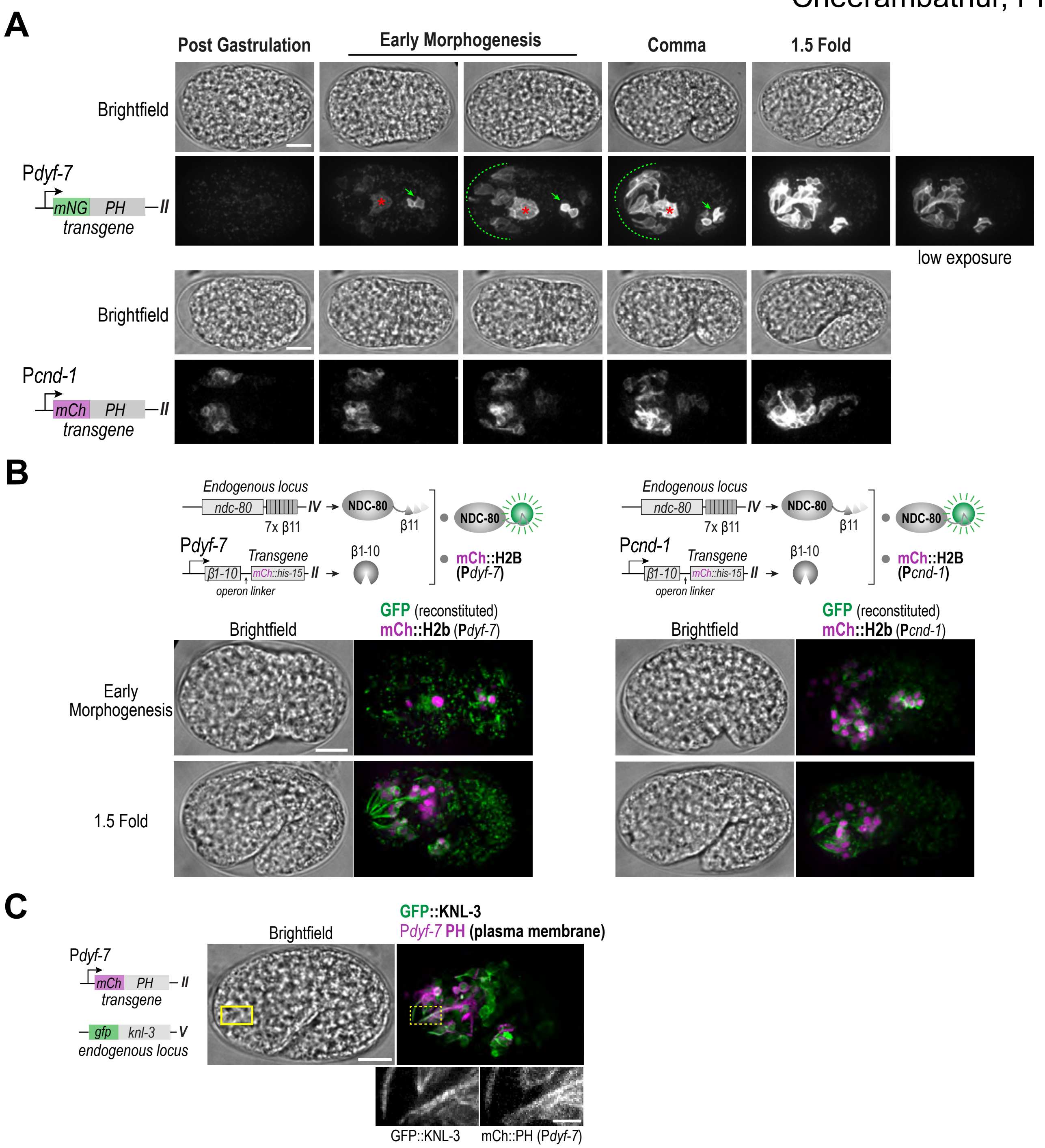

Figure. S2. Temporal and spatial profile of P*dyf-7* and P*cnd-1* activation, overlap of NDC-80 with P*dyf-7* and P*cnd-1* activation (Related to Figure 1).

(A) Images of fluorescent plasma membrane marker (the pleckstrin homology (PH) domain of mammalian Plc1δ1) expressed under control of P*dyf-7* or P*cnd-1* from a single copy transgene insertion. Localization similar to KMN proteins is highlighted (arrow & dashed line); red asterisk marks the excretory cell. Scale bar 10 µm. **(B)** Split GFP analysis using P*dyf-7* or P*cnd-1* to control expression of β1-10 of GFP in embryos where the *ndc-80* locus was engineered to fuse 7 copies of β11 to the NDC-80 C-terminus. The 1.5-fold stage embryo shown for P*dyf-7* is the same as in Figure 1E. Scale bar, 10 µm. **(C)** Image of 1.5-fold embryo with *in situ* GFP-tagged KNL-3 and P*dyf-7* controlled mCherry::PH plasma membrane marker. Region highlighted with yellow boxes is magnified below. mCherry maturation in embryos was slow and it was prone to aggregation, which greatly limited its utility for co-localization analysis. Scale bars: 10 µm for full embryo and 2.5 µm for insets.

Figure. S3

Cheerambathur, Prevo et al.

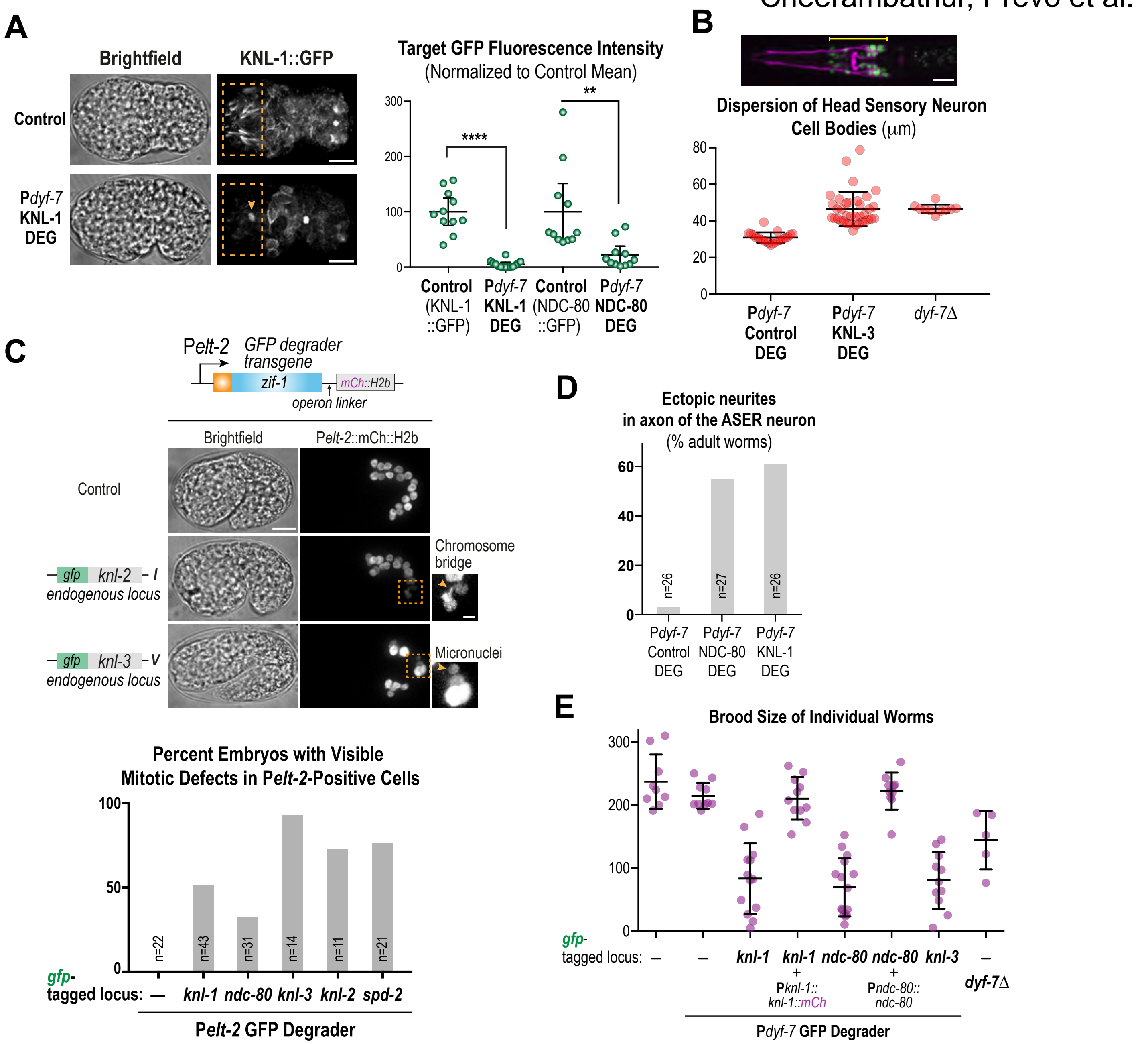

Figure. S3. GFP degrader analysis with P*dyf-7* and the intestinal promoter P*elt-2*, and phenotypes associated with P*dyf-7* controlled KMN degradation (Related to Figure 2). **(A)** Images and quantification of total GFP signal in the embryo anterior, measured during the early morphogenesis phase in embryos. The GFP signal in the embryo anterior (orange boxes) was quantified by integrating the fluorescence intensity above a threshold of 10000 a.u., while excluding GFP signal arising from kinetochore localization (arrowhead). Whole embryo images are for the anterior panels shown in Figure 2A. Scale bar, 10 µm. **(B)** Effect of KNL-3 degradation by the P*dyf-7* degrader or *dyf-7*Δ on sensory nervous system structure. The control data plotted is the same as in Figure 2C. **(C)** Mitotic defects following degradation of *in situ* GFP-tagged KMN, KNL-2 and SPD-2 in dividing cells during intestinal development, using a P*elt-2* controlled GFP degrader. P*elt-2* is activated in early intestinal development; the transgene includes a red fluorescent histone (separated from the GFP degrader by an operon linker) to mark the nuclear DNA of the cells in which the P*elt-2* promoter is active (Wang et al., 2017). Visible chromatin bridges and micronuclei (magnified on the right) in the mCh::H2b channel were scored as mitotic defects. The percentage of embryos with visible mitotic defects for the different targets of the P*elt-2* GFP degrader are plotted below. As not all mitotic defects result in visible bridges or micronuclei, the measured percentage is likely an underestimate. Scale bars: 10 µm (primary panels); 2.5 µm (magnified insets). **(D)** Quantification of ectopic neurite frequency in the ASER neuron for the indicated conditions. **(E)** Brood size of individual worms for the indicated conditions.

Figure. S4

Cheerambathur, Prevo et al.

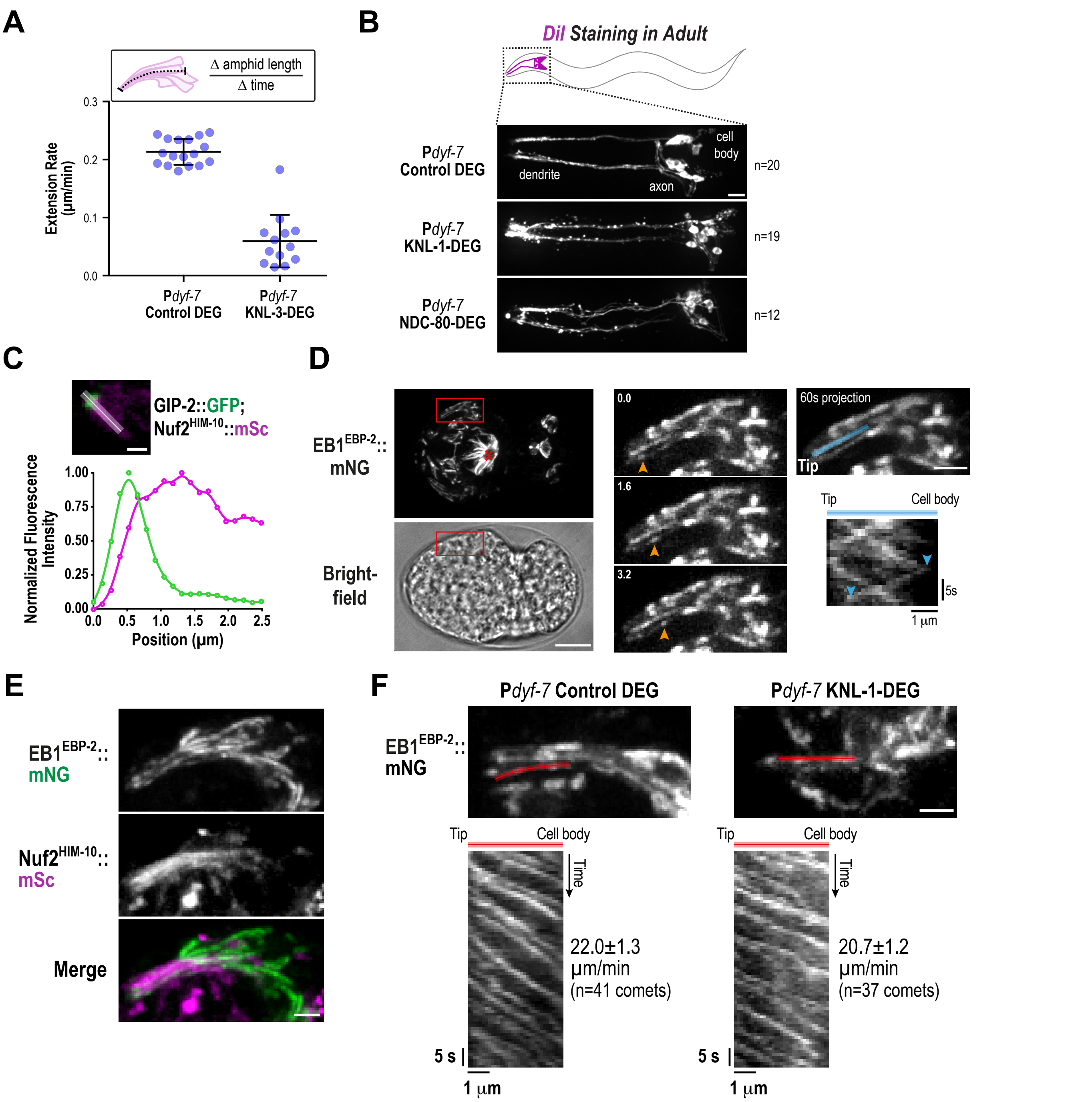

Figure. S4. Analysis of amphid dendrites: role of KNL-3, dye-filling and microtubule dynamics (Related to Figure 3).
(A) Dendrite extension rate following P*dyf-7* controlled KNL-3 degradation. Control data plotted is the same as in Figure 3C. **(B)** Dye-fill assay in adult worms for the indicated conditions. Following P*dyf-7* controlled degradation of KNL-1 and NDC-80, the amphid neurons were able to dye-fill indicating they have assembled cilia. Scale bar, 10 μm. **(C)** 3 pixel-wide linescan profile along dendrite of lower inset. Scale bar,1 μm (ii) in Figure 3D. Scale bar, 1 μm. **(D)** $EB1^{EBP-2}$ dynamics in amphid bundles. Whole embryo images highlight the amphid bundle magnified in Figure 3E and to the right. Asterisk marks the excretory cell, in which P*dyf-7* is active but KMN proteins are not expressed. Middle column shows fast timelapse images (time is in seconds); arrows point to a microtubule plus end comet. The kymograph for this dendrite is shown in Figure 3E. Column on the right shows a kymograph with plus end trajectories in both directions, indicating mixed polarity. The image for the 60s projection is the same as in Figure 3E. Scale bar for whole embryo images: 10 μm; for amphid bundles: 2.5 μm. **(E)** 2-color localization analysis of $EB1^{EBP-2}$ and *in situ* mScarlet-tagged $Nuf2^{HIM-10}$. Scale bar, 2 μm. **(F)** $EB1^{EBP-2}$ dynamics in P*dyf-7* Control DEG and P*dyf-7* KNL-1 DEG amphid bundles. Scale bar, 2 μm. Red lines indicate location of kymographs depicted below. Mean and 95% CI of growth velocity measure-mements is shown to the right of each kymograph.

Figure. S5

Cheerambathur, Prevo et al.

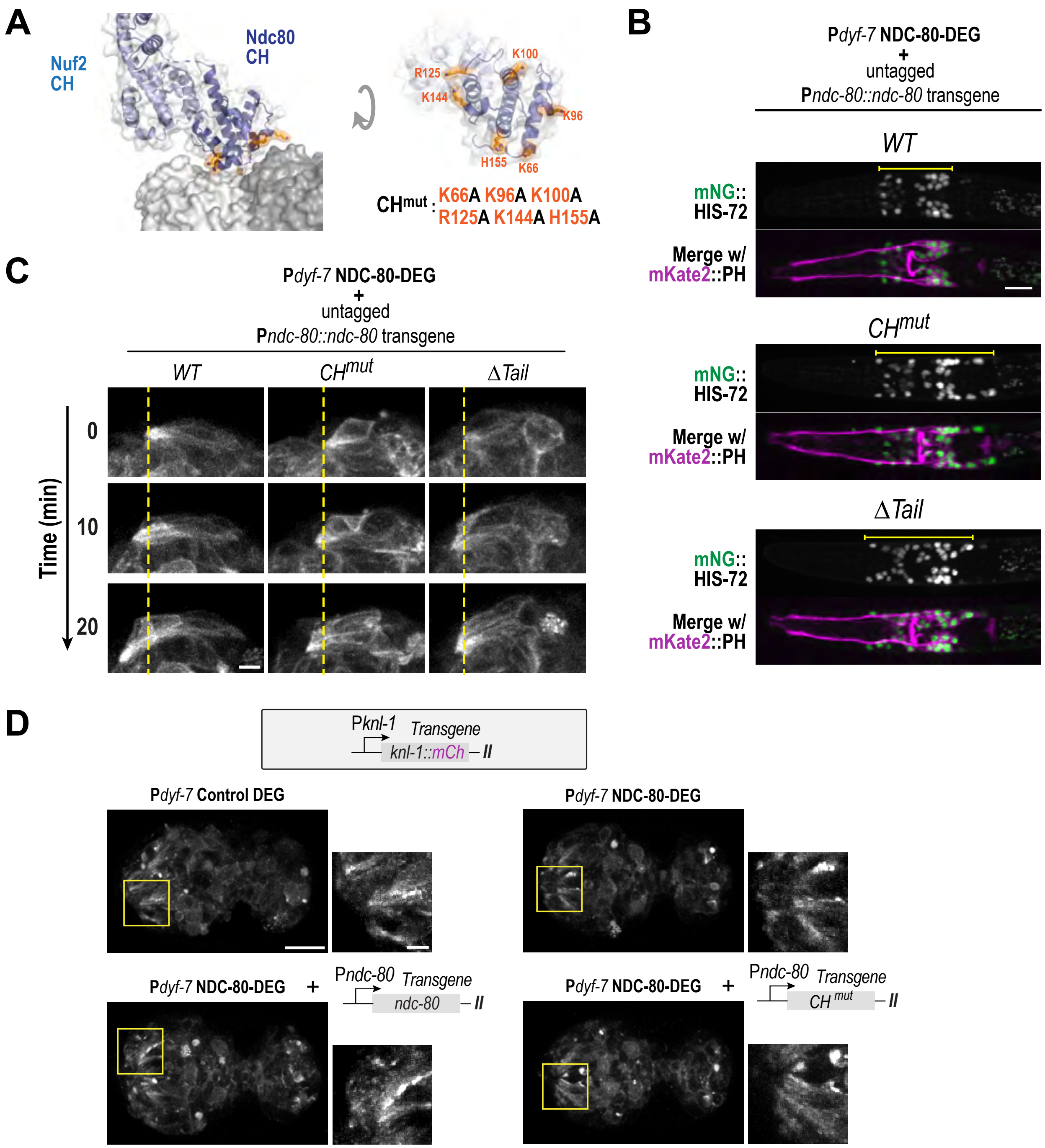

Figure. S5. Analysis of NDC-80 complex microtubule binding mutants (Related to Figure 5). **(A)** Structure of human Ndc80 complex bound to the microtubule surface (PDB: 3IZ0). Residues of the Ndc80 CH domain critical for microtubule binding are indicated on the rotated, *en face* view on the right. Amino acid numbers are for *C. elegans* NDC-80. **(B)** Sensory nervous system architecture in the L1 larval head visualized as in Figure. 2B. Scale bar, 10 µm. **(C)** Amphid bundle dendrite extension analysis. The WT images as the same as in Figure. 3B. Scale bar, 2.5 µm. **(D)** KNL-1::mCherry localization. Scale bar, 10 µm (embryo); 2.5 µm (inset).

References:

Gilleard, J.S., Shafi, Y., Barry, J.D., McGhee, J.D., 1999. ELT-3: A Caenorhabditis elegans GATA factor expressed in the embryonic epidermis during morphogenesis. Dev. Biol. 208, 265–280. doi:10.1006/dbio.1999.9202

Hallam, S., Singer, E., Waring, D., Jin, Y., 2000. The C. elegans NeuroD homolog cnd-1 functions in multiple aspects of motor neuron fate specification. Development 127, 4239–4252.

Heiman, M.G., Shaham, S., 2009. DEX-1 and DYF-7 establish sensory dendrite length by anchoring dendritic tips during cell migration. Cell 137, 344–355. doi:10.1016/j.cell.2009.01.057

Krause, M., Fire, A., Harrison, S.W., Priess, J., Weintraub, H., 1990. CeMyoD accumulation defines the body wall muscle cell fate during C. elegans embryogenesis. Cell 63, 907–919.

Table S1: CRISPR-Cas9 Protocol & sgRNA Sequences Used for Strain Generation (Related to STAR Methods)

STRAIN NAME	GENOTYPE	METHOD & REFERENCE	sgRNA SEQUENCE
OD2953	*him-10(lt52[him-10::GFP])III*	Direct Integration (Waaijers et al., 2013)	ACGGAAAAACTCGAATCGTT
OD3026	*knl-1(lt53[knl-1::GFP::tev::loxP::3xFlag])III*	SEC (Dickinson et al., 2015)	TCGAATGCTGGTGTTCTCTA
OD3029	*ndc-80(lt54[ndc-80::GFP::tev::loxP::3xFlag])IV*	SEC (Dickinson et al., 2015)	ATGTGCTGGCATTGAAAAGG
OD3101	*knl-3(lt46 [GFP::knl-3])V*	Direct Integration (Waaijers et al., 2013)	CGCGGCTCTGACCGAGAATG
OD3230	*air-2(lt58[air-2::GFP::tev::loxP::3xFlag])I*	SEC (Dickinson et al., 2015)	TTTTTGCCTCCATCATTCCC, GGAAGGAACACTACTGGATCC
OD3244	*dyf-7(lt60)X*	Ribonucleoprotein complex (Paix, et al., 2015)	CTTCAAGTATGAATCAATTG, TTTGGCAGGTGTCCAGTGAA
OD3367	*rod-1(lt62[GFP::rod-1)IV*	Direct Integration (Waaijers et al., 2013)	CCACAGCTTTTGCTTCGCCT
OD3407	*knl-2(lt73[GFP::knl-2])I*	Direct Integration (Waaijers et al., 2013)	CATCTACTAATCTCTGTGCA
OD3453	*spd-2(lt76[gfp::spd-2]) I*	Direct Integration (Waaijers et al., 2013)	TATTCTCAGCGTATTAAAA, TGTTCATTACAGAGATTCAT
OD3410	*hcp-4(lt72[GFP::hcp-4])I*	Direct Integration (Waaijers et al., 2013)	ACAATCGTACTGCGGGTTCG
OD3463	*hcp-3(lt78[GFP::hcp-3])I*	Direct Integration (Waaijers et al., 2013)	CGATGACACCCCAATTATTG
OD3516	*bub-1(lt82 [bub-1::GFP])I*	Direct Integration (Waaijers et al., 2013)	TCATTGTGTTGGGCTACTTT, TTGGTTGGCGGCAAGATCAC
OD3995	*ndc-80[lt126 (ndc-80::7Xβ-11)] IV*	Direct Integration (Waaijers et al., 2013)	ATGTGCTGGCATTGAAAAGG
OD4040	*him-10[lt130(him-10::mSca)] III*	Direct Integration (Waaijers et al., 2013)	ACGGAAAAACTCGAATCGTT

Table S2: Sequences of Regulatory elements & Fluorescent Probes (Related to STAR Methods)

Regulatory element	Length	5' end	3'end
Pelt-2	2912 bp	5'-tacatctttaccggcaccagaaga-3'	5'-agaaactagaaaatagattataga-3'
Pelt-3	2507 bp	5'-cacgttgtttcacggtcatcgtcg-3'	5'-tatcgagtggaaaaagtggccaac-3'
Phlh-1	3345 bp	5'-tgggttaatgtaggtgctggaagg-3'	5'-aattttccagaaatgaacacggaa-3'
Pcnd-1	3230 bp	5'-cagctatgacacgtggctctagta-3'	5'-tgtcatccagttatattttctaca-3'
Pdyf-7	3324 bp	5'-ttcatatactttatgtacggcgta-3'	5'-ctatttcagatttaaacttcaagt-3'
Pnphp-4	874 bp	5'-aaatcagggaaagtacatttttga-3'	5'-tttgtggtaacaaagtctcgaaaa-3'
Pgcy-5	3453 bp	5'-gcggtcaactagtgtatgattcct-3'	5'-aaaaattacttattctgatgaaaa-3'
unc-54 3'UTR	699 bp	5'-gtccaattactcttcaacatccct-3'	5'-ccaatataccaaacataactgttt-3'
dyf-7 3'UTR	1203 bp	5'-aataccgccattcacctcttatttt-3'	5'-cttggtctttcttttgttttagaa-3'
tbb-2 3'UTR	330 bp	5'-atgcaagatcctttcaagcattcc-3'	5'-gccgccaagaaaaaagtctcattg-3'
snb-1 3'UTR	663 bp	5'-gtacacgacctttgtcccggataa-3'	5'-ttagacggcacaataagccaccgg-3'

Sequences of Fluorescent Probes

Probe	Sequence
mScarlet	ATGGTCAGCAAGGGAGAGGCAGTTATCAAGGAGTTCATGCGTT<br>TCAAGGTCCACATGGAGGGATCCATGAACGGACACGAGTTCGA<br>GATCGAGGGAGAGGGAGAGGGACGTCCATACGAGGGAACCCA<br>AACCGCCAAGCTCAAGGTCACCAAGgtaagtttaaacatatatatactaact<br>aaccctgattatttaaattttcagGGAGGACCACTCCCATTCTCCTGGGACA<br>TCCTCTCCCCACAATTCATGTACGGATCCCGTGCCTTCACCAAGC<br>ACCCAGCCGACATCCCAGACTACTACAAGCAATCCTTCCCAGAG<br>GGATTCAAGTGGGAGCGTGTCATGAACTTCGAGGACGGAGGAG<br>CCGTCACCGTCACCCAAGgtaagtttaaacagttcggtactaactaaccatacat<br>atttaaattttcagACACCTCCCTCGAGGACGGAACCCTCATCTACAAG<br>GTCAAGCTCCGTGGAACCAACTTCCCACCAGACGGACCAGTCAT<br>GCAAAAGAAGACCATGGGATGGGAGGCCTCCACCGAGCGTCTC<br>TACCCAGAGGACGGAGTCCTCAAGGGAGACATCAAGATGGCCC<br>TCCGTCTCAAGGACGGAGGACGTTACCTCGCCGACTTCAAGgtaa<br>gtttaaacatgattttactaactaactaatctgatttaaattttcagACCACCTACAAGG<br>CCAAGAAGCCAGTCCAAATGCCAGGAGCCTACAACGTCGACCG<br>TAAGCTCGACATCACCTCCCACAACGAGGACTACACCGTCGTCG<br>AGCAATACGAGCGTTCCGAGGGACGTCACTCCACCGGAGGAAT<br>GGACGAGCTCTACAAG

splitGFP β1-10	ATGAGTAAAGGAGAAGAACTTTTCACTGGAGTTGTCCCAATTCTT<br>GTTGAATTAGATGGTGATGTTAATGGGCACAAATTTTCTGTCCGT<br>GGAGAGGGTGAAGGTGATGCAACAATCGGAAAACTTACCCTTA<br>AATTTATTTGCACTACTGGAAAACTACCTGTTCCATGGgtaagtttaa<br>acatatatatactaactaaccctgattatttaaattttcagCCAACACTTGTCACTAC<br>TCTTACCTACGGTGTTCAATGCTTCTCTAGATACCCAGATCACAT<br>GAAACGTCATGACTTTTTCAAGAGTGCCATGCCCGAAGGTTATG<br>TACAGGAAAGAACTATATCTTTCAAAGATGACGGGAAGTACAAG<br>ACACgtaagtttaaacagttcggtactaactaaccatacatatttaaattttcagGTGCT<br>GTCGTCAAGTTTGAAGGTGATACCCTTGTTAATAGAATCGAGTTA<br>AAAGGTACTGATTTTAAAGAAGATGGAAACATTCTTGGACACAA<br>ATTGGAATACAACTTCAACTCACACAATGTATACATCACCGCAGA<br>CAAACAAAAGAATGGAATCAAAGCTgtaagtttaaacatgattttactaacta<br>actaatctaatttaaattttcagAACTTCACCGTCAGACACAACGTCGAAGA<br>TGGAAGCGTTCAACTAGCAGACCATTATCAACAAAATACTCCAAT<br>TGGCGATGGCCCTGTCCTTTTACCAGACAACCATTACCTGTCCAC<br>ACAAACCGTCCTTTCGAAAGATCCCAACGAAAAG
splitGFP 7Xβ11	GGAGGGAGGGCCGGCTCTGGAGGCTCTGGAAGAGATCATATG<br>GTCCTCCACGAATACGTCAACGCTGCCGGGATCACTGGAGGAT<br>CTGGAGGACGTGACCATATGGTCCTCCACGAATACGTCAATGCC<br>GCCGGAATCACCGGAGGTTCCGGAGGACGTGATCACATGGTCC<br>TCCACGAATACGTCAACGCTGCCGGGATCACTGGAGGAAGCGG<br>AGGACGCGATCATATGGTCCTCCACGAGTACGTTAACGCCGCTG<br>GAATCACCGGAGGATCCGGAGGTAGAGACCATATGGTCCTTCA<br>CGAATACGTCAACGCCGCTGGAATCACCGGTGGATCCGGTGGA<br>CGTGATCACATGGTTCTTCATGAGTACGTTAACGCTGCTGGAAT<br>CACC
