# Supplementary material for: The Kinetochore-Microtubule Coupling Machinery Is Repurposed in Sensory Nervous System Morphogenesis
Source: Dev Cell. 2019 Mar 25;48(6):864–872.e7. doi: 10.1016/j.devcel.2019.02.002 (PMC6436928; doi:10.1016/j.devcel.2019.02.002)
Supplement: Document S2. Article plus Supplemental Information [file mmc4.pdf]

Developmental Cell

Short Article

The Kinetochore-Microtubule Coupling Machinery Is Repurposed in Sensory Nervous System Morphogenesis

Graphical Abstract

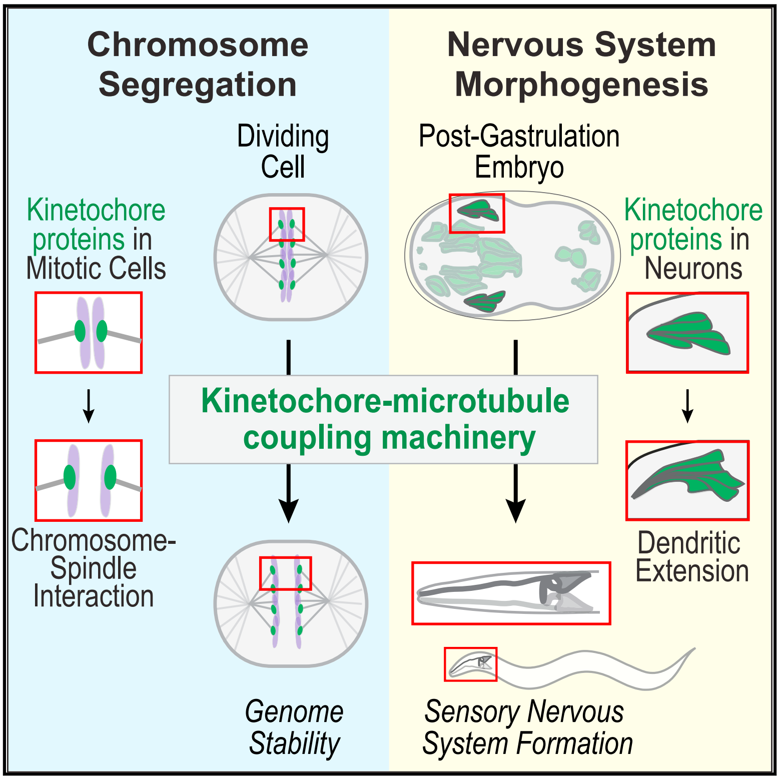

Highlights

- Kinetochore-microtubule coupling machinery acts in developing post-mitotic neurons
- Post-mitotic removal of kinetochore components disrupts the sensory nervous system
- Kinetochore components concentrate in sensory dendrites and promote their extension
- The microtubule-coupling interface in developing neurons and mitosis is similar

Authors

Dhanya K. Cheerambathur, Bram Prevo, Tiffany-Lynn Chow, ..., Karen Oegema, Rebecca Green, Arshad Desai

Correspondence

dhanya.cheerambathur@ed.ac.uk (D.K.C.), abdesai@ucsd.edu (A.D.)

In Brief

The conserved Knl1/Mis12/Ndc80 (KMN) network constitutes the microtubule-coupling interface at kinetochores in mitosis. Cheerambathur et al. find that the KMN network also acts in the developing sensory nervous system. KMN components concentrate in dendrites of developing neurons and promote their extension, employing the same microtubule-coupling interface that acts in mitosis.

Cheerambathur et al., 2019, Developmental Cell *48*, 864–872
March 25, 2019 © 2019 The Authors. Published by Elsevier Inc.
https://doi.org/10.1016/j.devcel.2019.02.002

CellPress

Developmental Cell
Short Article

The Kinetochore-Microtubule Coupling Machinery Is Repurposed in Sensory Nervous System Morphogenesis

Dhanya K. Cheerambathur,[1,2,3,4,*] Bram Prevo,[1,2,3] Tiffany-Lynn Chow,[1,2] Neil Hattersley,[1,2] Shaohe Wang,[1,2] Zhiling Zhao,[1,2] Taekyung Kim,[1,2,5] Adina Gerson-Gurwitz,[1,2] Karen Oegema,[1,2] Rebecca Green,[1,2] and Arshad Desai[1,2,6,*]
[1]Ludwig Institute for Cancer Research, San Diego Branch, La Jolla, CA 92093, USA
[2]Department of Cellular & Molecular Medicine, University of California, San Diego, San Diego, La Jolla, CA 92093, USA
[3]These authors contributed equally
[4]Present address: Wellcome Centre for Cell Biology & Institute of Cell Biology, School of Biological Sciences, The University of Edinburgh, Edinburgh EH9 3BF, UK
[5]Present Address: Department of Biology Education, Pusan National University, Pusan 609-735, Korea
[6]Lead Contact
*Correspondence: dhanya.cheerambathur@ed.ac.uk (D.K.C.), abdesai@ucsd.edu (A.D.)
https://doi.org/10.1016/j.devcel.2019.02.002

SUMMARY

Dynamic coupling of microtubule ends to kinetochores, built on the centromeres of chromosomes, directs chromosome segregation during cell division. Here, we report that the evolutionarily ancient kinetochore-microtubule coupling machine, the KMN (Knl1/Mis12/Ndc80-complex) network, plays a critical role in neuronal morphogenesis. We show that the KMN network concentrates in microtubule-rich dendrites of developing sensory neurons that collectively extend in a multicellular morphogenetic event that occurs during *C. elegans* embryogenesis. Post-mitotic degradation of KMN components in sensory neurons disrupts dendritic extension, leading to patterning and functional defects in the sensory nervous system. Structure-guided mutations revealed that the molecular interface that couples kinetochores to spindle microtubules also functions in neuronal development. These results identify a cell-division-independent function for the chromosome-segregation machinery and define a microtubule-coupling-dependent event in sensory nervous system morphogenesis.

INTRODUCTION

During cell division, the centromere regions of chromosomes assemble large protein machines called kinetochores to connect chromosomes to dynamic spindle microtubules (Cheeseman, 2014; Musacchio and Desai, 2017). At the kinetochore, the conserved 10-subunit Knl1 complex/Mis12 complex/Ndc80 complex (KMN) network is the primary chromosome-microtubule coupler (Cheeseman, 2014; Musacchio and Desai, 2017). The KMN network facilitates chromosome segregation by forming dynamic attachments to ends of polymerizing and depolymerizing spindle microtubules. The KMN network also ensures segregation fidelity by coordinating this mechanical coupling with control of cell cycle progression by acting as a scaffold for checkpoint signaling machinery.

Within the KMN network, the Ndc80 complex forms the primary microtubule coupling module (Cheeseman et al., 2006; Deluca et al., 2006), the Knl1 complex serves as a scaffold for the Ndc80 complex and signaling machinery, and the Mis12 complex links the KMN network to centromeric chromatin (Musacchio and Desai, 2017). At the kinetochore, KMN complexes generate a multivalent high-density microtubule-binding site that couples chromosomes to dynamic microtubule plus ends. Microtubule-coupling activity resides within the Ndc80 subunit of the Ndc80 complex, which harbors two distinct microtubule-interacting elements: a folded calponin homology (CH) domain that recognizes a specific site on the microtubule lattice (Alushin et al., 2010) and a basic unstructured N-terminal tail that provides electrostatic affinity to the negatively charged microtubule surface and mediates cooperative binding along the lattice (Alushin et al., 2012; Ciferri et al., 2008). To date, the sole known function for the specialized microtubule coupling and signaling roles of the KMN network is in chromosome segregation during cell division.

Here, we show that the KMN network serves a critical cell-division-independent function in establishing the proper architecture of the sensory nervous system during *C. elegans* embryogenesis. KMN components concentrate in the microtubule-rich dendrites of developing post-mitotic sensory neurons and their controlled, post-mitotic degradation perturbs initial dendritic extension of sensory neuron bundles, resulting in architectural and functional defects of the sensory nervous system. We additionally demonstrate that the function of the KMN network in sensory nervous system development requires the microtubule-coupling activity resident in the Ndc80 complex. These results identify a new role for the evolutionarily ancient chromosome-segregation machinery and define an early morphogenetic event that establishes the architecture and function of the sensory nervous system.

RESULTS

KMN Components Localize to Dendrites of the Developing Sensory Nervous System during Late Embryogenesis

A hint that the KMN network functions outside of its well-studied cell division context came from analysis of *in situ* GFP-tagged

 © 2019 The Authors. Published by Elsevier Inc.
This is an open access article under the CC BY license (http://creativecommons.org/licenses/by/4.0/).

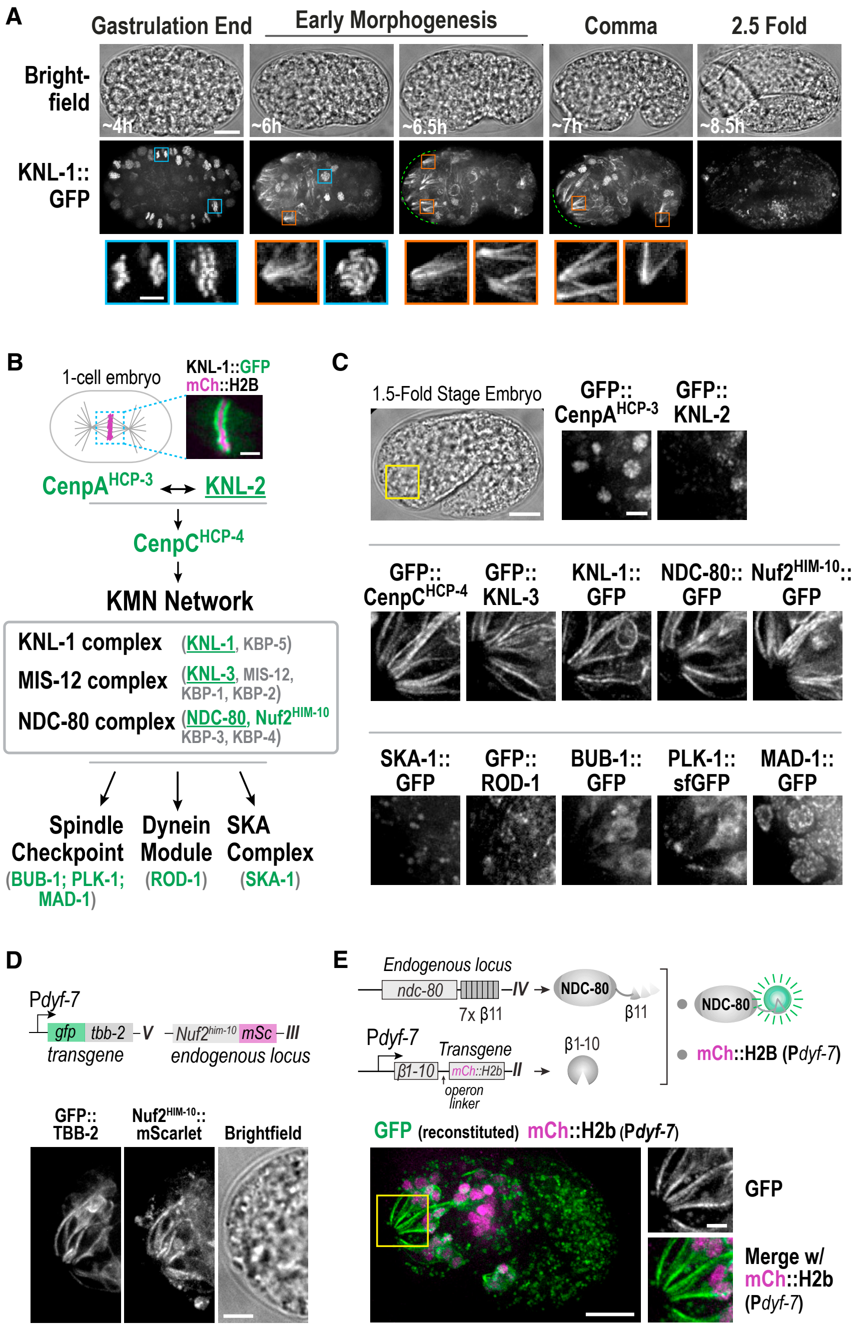

Figure 1. Kinetochore Proteins Concentrate in the Dendritic Extensions of Sensory Neurons

(A) Images of *in situ*-tagged KNL-1::GFP at different stages of embryogenesis. KNL-1 localized to chromosomes in dividing cells (blue boxes) and to non-chromosomal structures (orange boxes; dashed green line). Time is relative to first cleavage. Scale bars, 10 μm and 2 μm (insets).

(B) Kinetochore assembly in the early *C. elegans* embryo. Proteins labeled in green were *in situ* GFP-tagged for localization analysis; underlined proteins were subjected to functional analysis. Scale bar, 2 μm.

(C) Localization of indicated *in situ*-tagged GFP fusions in the anterior of 1.5-fold stage embryos. Scale bars, 10 μm (brightfield) and 2.5 μm (magnified anterior region).

(D) Localization of mScarlet-fused Nuf2^{HIM-10} and P*dyf-7*-controlled GFP::β -tubulin (TBB-2). Scale bar, 5 μm.

(E) Split GFP analysis with indicated complementing pieces. Scale bars, 10 μm and 2.5 μm (inset).

KNL-1 during *C. elegans* embryogenesis. KNL-1 exhibited the expected localization to kinetochores of dividing cells until the end of gastrulation (Sulston et al., 1983) (Figure 1A). However, as the morphogenetic events that structure the tissues and convert the embryonic cell mass into an elongated larva initiated, KNL-1 appeared on cytoplasmic filamentous structures that were prominent in the developing head region in the embryo anterior (Figure 1A). As the embryo began to elongate, the non-chromosomal localization of KNL-1 peaked between "comma" stage and "1.5-fold" stage before declining in intensity and was not observed at later embryonic stages or in L1 larvae (Figure 1A; data not shown). *In situ* GFP-tagging of 12 kinetochore components ranging from constituents of centromeric chromatin (CENPA^{HCP-3} and KNL-2) to the spindle checkpoint (BUB-1 and MAD-1; Figure 1B) revealed that while all components localized to kinetochores in dividing cells, only CENP-C^{HCP-4} and the KMN subunits KNL-3, NDC-80, and Nuf2^{HIM-10} exhibited non-chromosomal localization similar to KNL-1 (Figures 1C and S1A; this localization was also observed for Aurora B^{AIR-2} kinase (Figure S1B), which localizes to chromatin and the spindle midzone in dividing cells (Carmena et al., 2012; Oegema et al., 2001).

The pattern of KMN network localization suggested concentration in the microtubule-rich dendrites of developing sensory neurons (Figures 1D, S1C, and S2A) (Heiman and Shaham, 2009). Imaging of embryos with cell-type-specific promoters driving nuclear and plasma membrane markers indicated that the P*dyf-7* promoter, which is active during early morphogenesis in the majority of sensory neurons in the head (Heiman and Shaham, 2009), best mimicked the spatiotemporal pattern of KMN localization (Figures S1D, S1E, and S2C). To confirm that the non-chromosomal localization of KMN is in developing sensory neurons, we used the split GFP system (Cabantous et al., 2005; Kamiyama et al., 2016). The non-chromosomal localization of NDC-80 in the developing head was reconstituted by expressing complementing elements of GFP from the endogenous *ndc-80* locus and from a P*dyf-7*-controlled transgene (Figures 1E and S2B). These observations suggest a chromosome segregation-independent role for the

kinetochore-microtubule coupling machinery in the developing nervous system.

Post-Mitotic Degradation of KMN Components Perturbs Sensory Nervous System Architecture and Function

To assess KMN function in developing sensory neurons, we used the P*dyf-7* promoter to express a GFP degrader (Caussinus et al., 2011; Wang et al., 2017) in embryos where *in situ* tagged GFP fusions were the sole source of KNL-1 or NDC-80 (Figure 2A). Imaging of the resulting embryos confirmed loss of the filamentous GFP signal in the head region of the embryo (Figures 2A and S3A). As P*dyf-7* is activated after cell division ceases in the majority of sensory neurons (Heiman and Shaham, 2009), this approach enabled assessment of the post-mitotic role of KMN proteins in sensory nervous system development. Embryos expressing the GFP degrader without an *in situ* GFP-tagged target (P*dyf-7* Control DEG) served as a control. To assess the effect of loss of KNL-1 or NDC-80, we visualized the sensory nervous system in L1 larvae expressing nuclear and plasma membrane markers in ciliated sensory neurons (Figure 2B) (Winkelbauer et al., 2005). In control worms, sensory neuron cell bodies are tightly clustered in a ~30 μm wide region on either side of the nerve ring. This stereotypical architecture was highly perturbed after degradation of KNL-1 or NDC-80 (Figures 2B and 2C). A similar phenotype was observed following degradation of the KMN component KNL-3 and in a *dyf-7* mutant (Figure S3B) but not with the essential cell division proteins KNL-2 or SPD-2, which are required for kinetochore and centrosome function, respectively (Kemp et al., 2004; Maddox et al., 2007) (Figure 2C); of note, KNL-2 and SPD-2 degradation in the developing intestine revealed mitotic defects similar to KNL-1 or NDC-80 degradation (Figure S3C), indicating that both were susceptible to the action of the GFP degrader. GFP degrader-resistant versions of KNL-1 or NDC-80 expressed under control of endogenous regulatory sequences or P*dyf-7* fully rescued the sensory nervous system architecture defect (Figures 2C and 2D).

To examine the effect of KNL-1 or NDC-80 degradation at a cellular level, we also visualized the morphology of an individual sensory neuron by expressing markers, including the synaptic marker SNB-1, specifically in the ASER neuron (Nonet, 1999). This approach revealed defects in axonal morphology as well as the presence of ectopic neurites along the axon (Figures 2E and S3D). The P*dyf-7*-controlled degradation of KMN components resulted in gross phenotypes associated with impairment of the sensory nervous system, most notably defective egg-laying and reduced fertility (Figures 2F and S3E) (Schafer, 2006). These phenotypes were also rescued by GFP degrader-resistant versions of KNL-1 and NDC-80 (Figures 2F and S3E). Given the severe disorganization of the sensory nervous system, we did not pursue more sophisticated sensory assays (Bargmann et al., 1993; Ward, 1973). We conclude that KMN components have a cell-division-independent function in developing neurons that is required to establish proper sensory nervous system architecture.

KMN Components Act during the Initial Dendritic Extension of the Amphid Neuron Bundles

KMN proteins are not present in head sensory neurons after the 2-fold stage in embryogenesis, indicating that the defects observed in L1 larvae are a consequence of KMN inhibition at an earlier developmental stage. To understand how the KMN network contributes to the morphogenesis of the sensory nervous system, we therefore analyzed the effects of post-mitotic KMN inhibition at the time when the localization of KMN components in developing neurons is most prominent (Figure 1A). During the early stages of embryo elongation, two clusters of 12 sensory neurons (called amphid bundles) positioned on either side of the embryo collectively extend microtubule-rich dendritic projections toward the embryo anterior (Figures 3A and 3B) (Heiman and Shaham, 2009). Time-lapse imaging revealed that the rate of amphid bundle dendritic extension toward the embryo anterior was significantly reduced following degradation of KMN proteins (Figures 3B, 3C, and S4A; Video S1); in addition, the organization of the cell bodies within the amphid bundles was abnormal (data not shown). Both phenotypes were rescued by transgenes encoding GFP degrader-resistant KMN components (Figures 3B and 3C; Video S1). Thus, the KMN network is required for a multicellular morphogenetic event during embryogenesis in which bundles of sensory neurons collectively extend microtubule-rich dendritic projections.

The established function of the KMN network in microtubule coupling at kinetochores and the presence of microtubules in the extending dendritic projections (Figure 1D) suggested that KMN function in sensory neurons is microtubule related. To test this idea, we first assessed the role of microtubules in the extension of dendritic projections by the amphid bundles by degrading GIP-2, an essential subunit of the microtubule-nucleating γ-tubulin complex (Roostalu and Surrey, 2017; Wang et al., 2015). GIP-2 degradation resulted in a dendrite extension defect similar to KNL-1 or NDC-80 degradation (Figures 3B and 3C), consistent with an essential role for microtubules. We note that following KMN inhibition, sensory neurons in L1 larvae are mispositioned, but dendrites are not substantially reduced in length (Figures 2B and S4B). This is in contrast to the inhibition of GIP-2 (data not shown) or DYF-7, both of which lead to a reduction in dendrite length (Heiman and Shaham, 2009). This comparison suggests that in addition to the KMN-dependent mechanism that positions sensory neurons and promotes dendrite extension, other mechanisms contribute to dendrite extension later in embryogenesis. If the earlier KMN-based mechanism is defective, these later mechanisms still promote dendrite extension but are unable to correct defects in neuronal positioning.

Given the role of the KMN network in coupling to dynamic microtubules at kinetochores, we analyzed the microtubule cytoskeleton within extending amphid bundle dendrites by imaging GIP-2 and the microtubule plus end-tracking protein EB1^{EBP-2}. GIP-2 was prominently concentrated in dendrite bundle tips, unlike KMN proteins, which localized along the dendrite length (Figures 3D and S4C). EB1^{EBP-2} comets emerged from the tip region and were primarily directed toward cell bodies (Figures 3E and S4D; Video S2); this organization is consistent with reported predominance in dendrites of microtubules with their minus ends facing out and their plus ends extending toward the cell body (Yan et al., 2013). EB1^{EBP-2} also formed clusters along the length of the amphid bundle (Figure 3E) whose precise nature is unclear; these clusters were non-overlapping with KMN proteins in the extending dendrites (Figure S4E). Following KMN degradation, the shorter dendrites and organizational defects made quantitative comparisons of EB1 dynamics and distribution difficult; however,

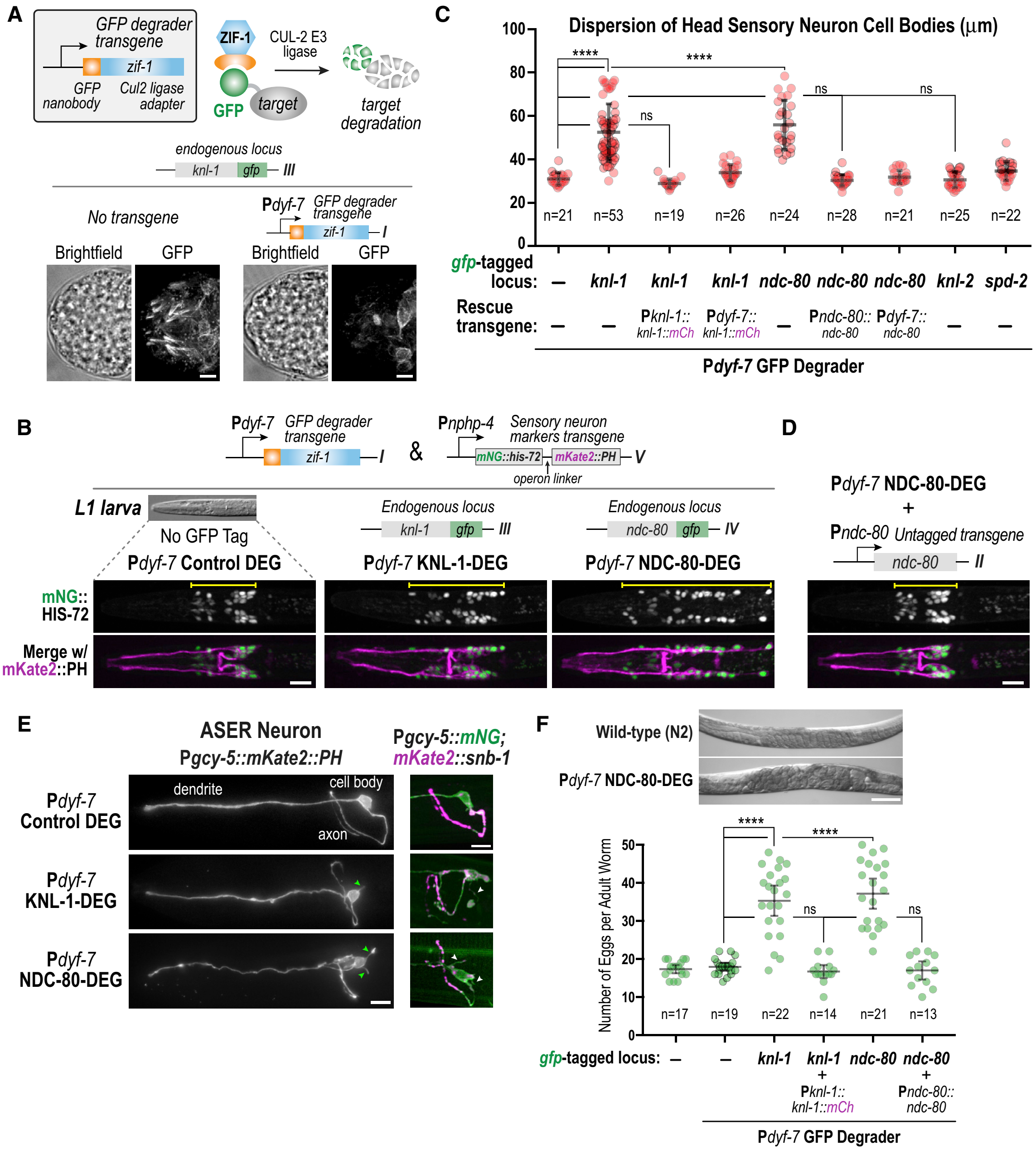

Figure 2. Post-Mitotic Degradation of KMN Proteins Causes Sensory Nervous System Defects

(A) Approach used to degrade *in situ* GFP fusions (top). Images below show loss of KNL-1::GFP signal in presence of a P*dyf-7*-controlled GFP degrader. Scale bar, 5 μm.

(B) Images of head sensory neuron nuclei and plasma membranes for the indicated conditions. Scale bar, 10 μm.

(C) Quantification of dispersion of sensory neuron cell bodies, measured as indicated with yellow lines in (B). Error bars denote the 95% confidence interval. **** and ns indicate $p < 0.0001$ and not significant, respectively.

(D) Rescue of sensory nervous system defect by transgene-encoded untagged NDC-80. Scale bar, 10 μm.

(E) Profile of ASER neuron (left) and localization of the synaptic marker SNB-1 (right) in the ASER neuron in the indicated conditions. Arrowheads point to ectopic neurites. Scale bar, 10 μm.

(F) Egg-laying defect: example image (top) and quantification for indicated conditions (bottom). Scale bar, 100 μm. Error bars denote the 95% confidence interval. **** and ns indicate $p < 0.0001$ and not significant, respectively.

observed EB1 comets exhibited normal velocity (Figure S4F). Overall, these observations indicate that the KMN network is critical for proper extension of microtubule-rich dendrites and suggest that they provide a microtubule-related function during this early event in the development of sensory neurons.

Microtubule Binding Elements of the NDC-80 Complex Are Required for the Function of the KMN Network in Sensory Nervous System Development

As direct imaging of the microtubule cytoskeleton in amphid bundle dendrites after post-mitotic KNL-1 or NDC-80 degradation

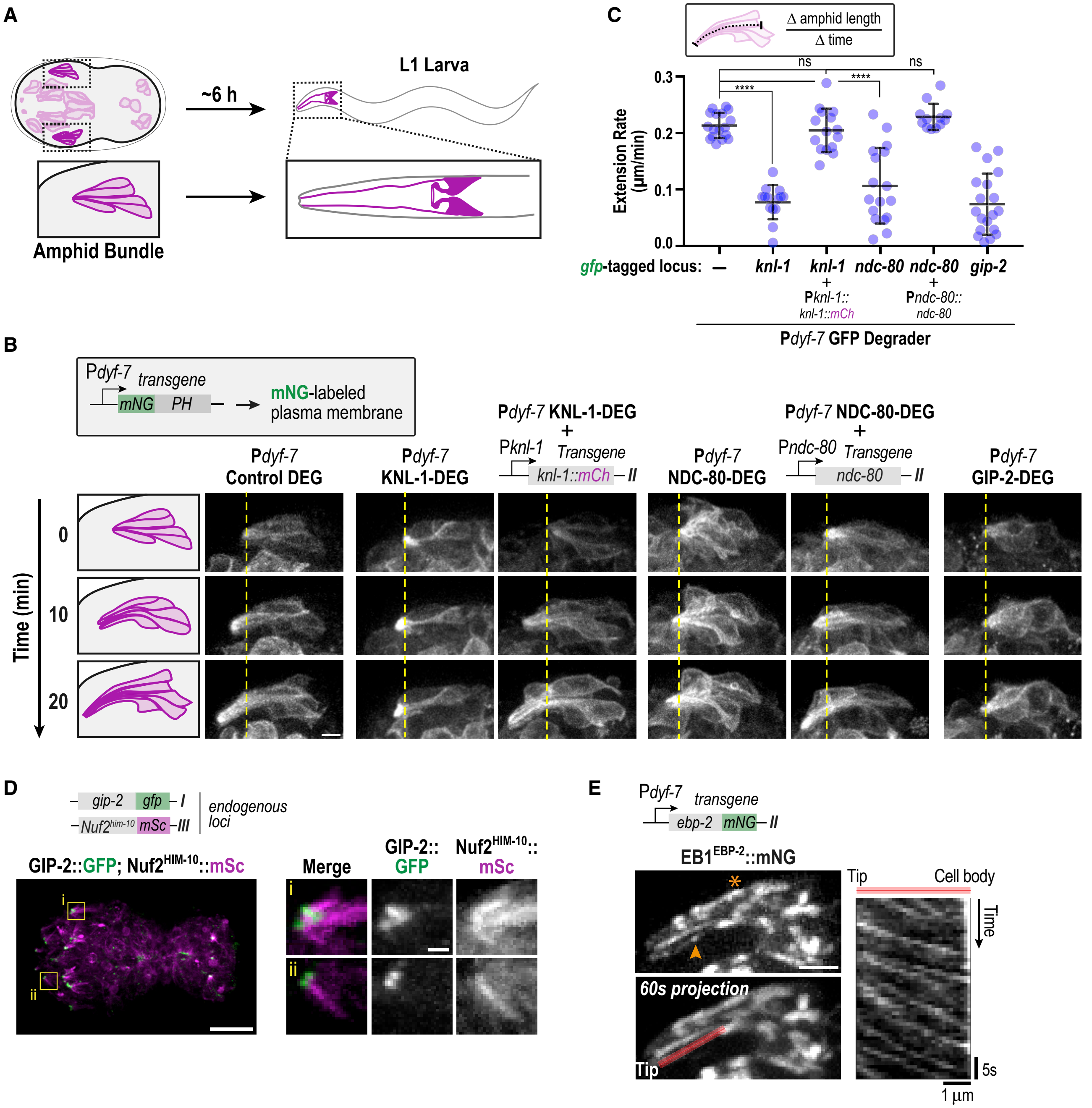

Figure 3. KMN Proteins Are Important for Amphid Bundle Dendrite Extension

(A) Schematic relating amphid sensilla in the embryo to their eventual structure in the L1 larva (each sensilla has 12 neurons; only 5 are depicted).

(B) Live imaging of amphid bundle dendrite extension for the indicated conditions using a P*dyf-7* controlled plasma membrane marker. Scale bar, 2.5 µm.

(C) Quantification of amphid bundle dendrite extension rate. Error bars denote the 95% confidence interval. **** and ns indicate $p < 0.0001$ and not significant, respectively.

(D) Color overlay of gamma-tubulin complex (GIP-2::GFP) and KMN protein ($Nuf2^{HIM-10}$::mSc) localization. Magnified insets with individual channel signals are shown on the right. Scale bars, 10 µm (embryo) and 1 µm (inset). Line profile along dendrite of bottom inset is shown on the right.

(E) $EB1^{EBP-2}$::mNG dynamics in the amphid bundle. A plus end comet (arrowhead) and a cluster (asterisk) are highlighted in the top still image. Kymograph along the red line in the 60 s time projection image (bottom) is shown on the right. Scale bar, 2.5 µm.

proved challenging, we took a different approach to address if the role of the KMN network in sensory nervous system morphogenesis involves its microtubule-coupling activity. For this purpose, we relied on prior studies in chromosome segregation, which established a critical role for microtubule lattice recognition by a conserved set of 6 residues on the surface of NDC-80's CH

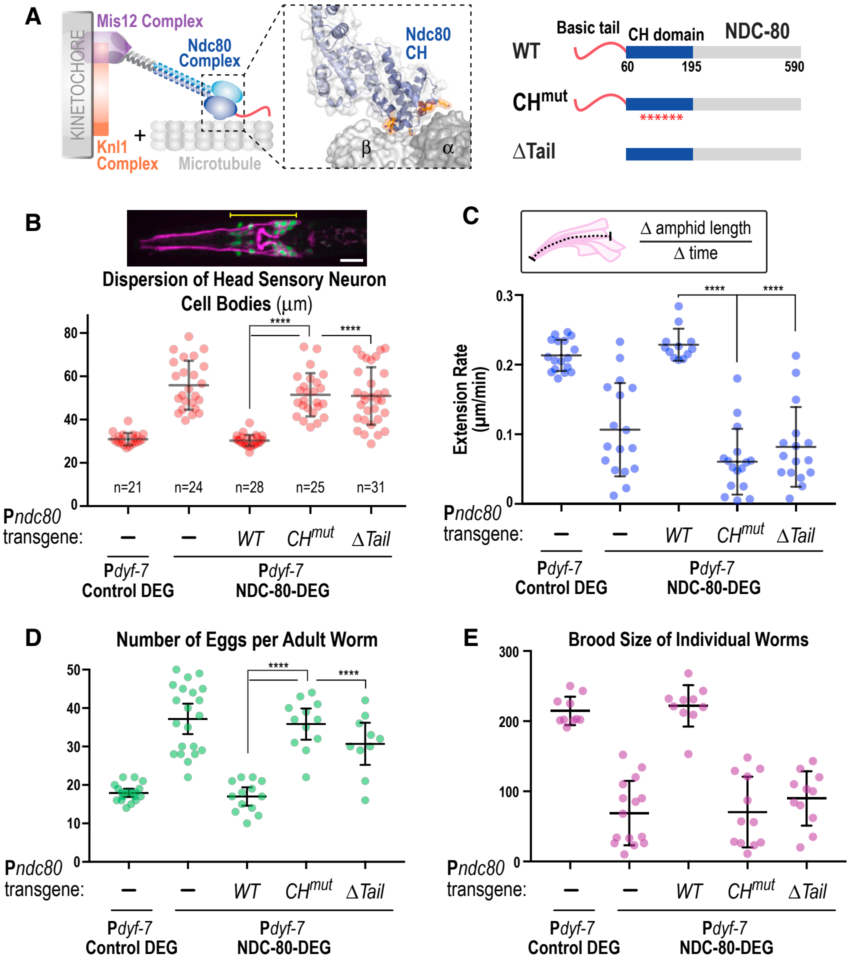

Figure 4. Microtubule-Binding Elements of the KMN Network Are Required for Function in the Developing Sensory Nervous System

(A) Schematic highlighting the two microtubule-binding elements of the KMN network: the NDC-80 CH domain that docks onto the microtubule surface and the NDC-80 N-terminal basic tail. Untagged wild-type and mutant NDC-80 variants expressed by single copy transgenes are indicated on the right.

(B–E) Phenotypic analysis of transgene-encoded NDC-80 variants following P*dyf-7*-mediated degradation of endogenous GFP-tagged NDC-80. P*dyf-7* Control DEG, P*dyf-7* NDC-80 DEG, and P*dyf-7* NDC-80 DEG plus WT NDC-80 data are the same as in Figures 2C, 2F, and 3C. Scale bar, 10 μm. Error bars denote the 95% confidence interval. **** and ns indicate $p < 0.0001$ and not significant, respectively.

domain (Alushin et al., 2010; Ciferri et al., 2008) (Figures 4A and S5A). By expressing degrader-resistant NDC-80 variants at levels equivalent to endogenous NDC-80 (Cheerambathur et al., 2013, 2017) while degrading *in situ* GFP-tagged endogenous NDC-80, we found that the CH domain mutant of NDC-80 fully phenocopied loss of NDC-80 in sensory nervous system architecture (Figures 4B and S5B), amphid bundle dendrite extension (Figures 4C and S5C), egg-laying, and fertility (Figures 4D and 4E). NDC-80 harbors a second *in vitro* microtubule-binding entity, a basic N-terminal tail (Figure 4A) that does not contribute substantially to chromosome segregation in *C. elegans* (Cheerambathur et al., 2017). In contrast to chromosome segregation in early embryos, deletion of the NDC-80 N-terminal tail had an impact comparable to the NDC-80 CH domain mutant in the developing sensory nervous system (Figures 4B–4D and S5B–S5D). As the NDC-80 N-tail is implicated in cooperative binding to the surface of the microtubule (Alushin et al., 2012; Ciferri et al., 2008), this result suggests that N-tail-mediated cooperative microtubule lattice binding may be more critical in the context of KMN network function in the nervous system than in chromosome segregation. We conclude that both microtubule-binding elements of the NDC-80 complex are essential for the post-mitotic function of the KMN network in sensory nervous system development.

We next addressed if disrupting microtubule-binding activity of NDC-80 affects the dendritic localization of other KMN network components. This analysis was challenged by slow red fluorescent protein maturation as well as aggregation of red fluorescent protein fusions; the same targets (e.g., KNL-1) fused to GFP did not exhibit aggregation. Crossing KNL-1::mCherry into strains where NDC-80 was degraded and replaced with untagged wild-type or CH mutant NDC-80, we found that, even when dendritic extension was impaired, KNL-1::mCherry still localized to dendrites (Figure S5D). With the caveat of the weaker signal due to issues with the fluorophore, this observation suggests that a microtubule binding-independent mechanism analogous to centromeric chromatin in dividing cells may recruit the MIS-12 complex and KNL-1 to the developing dendrites.

DISCUSSION

Chromosome segregation and neuronal morphogenesis are two processes in which the microtubule cytoskeleton plays an integral role. Here, we show that the KMN network, the ancient microtubule-coupling machine that evolved to harness the intrinsic dynamics of microtubule polymers to drive chromosome segregation, has been repurposed to act during a specific early step in sensory nervous system morphogenesis. Our findings suggest that the microtubule coupling function of the KMN network is required for the initial stages of dendritic extension during embryogenesis, which in turn is critical for establishing the proper architecture and function of the sensory nervous system. While our efforts focused on the sensory nervous system, KMN proteins are also expressed in other neuron types (e.g., ventral nerve cord precursors; Figure S2B) and extending their analysis to other classes of neurons that form during embryogenesis as well as post-embryonically will be important. In a study in *Drosophila*

embryos (Zhao et al., 2019), kinetochore proteins were independently identified as being important for nervous system architecture, suggesting potential conservation of the function we describe here.

Defining the precise spatial relationship between KMN proteins and the microtubule network in dendrites will be important to understand their function in nervous system development. The amphid bundles have dendrites from 12 neurons that are tightly bundled; in addition, these bundles are located at some depth in the embryo (the precise depth varies based on embryo orientation), which causes signal and resolution loss. In addition, we faced unexpected difficulties with the use of red fluorescent proteins at this stage in embryogenesis. mCherry, mKate2, and mScarlet all mature slowly (relative to GFP; in multiple cases, we have the same protein *in situ* tagged with GFP or one or more of the red fluorescent proteins, which allowed directly comparisons) and they formed aggregates; the same red fluorescent protein fusions did not exhibit aggregation in early embryos. While we do not understand why aggregation occurs in later stage embryos, it limited experiments involving red-green fluorescent protein pairs at the stage of embryogenesis when the sensory nervous system is developing. Thus, the precise spatial relationship between KMN proteins and microtubules will need to be defined in the future using better probes and more advanced imaging modalities.

The role of the KMN network in sensory neuronal development during embryogenesis is distinct from well-studied neuronal microtubule-binding proteins that stabilize different classes of microtubules in mature neurons (Kapitein and Hoogenraad, 2015). We show that the neuronal function of the KMN network requires the conserved NDC-80 CH domain-microtubule lattice interaction, which is central to forming a dynamic microtubule end-coupled interface during chromosome segregation. We find that dendritic extension of developing sensory neurons also requires the unstructured basic N-terminal tail of NDC-80, which mediates cooperative binding to microtubules *in vitro* but whose function in chromosome segregation is less clear. We suggest that cooperative binding of KMN complexes along the lattice may be particularly significant in the context of neuronal development. While the mutant analysis establishes the importance of microtubule-coupling elements of NDC-80, their precise contribution during dendritic extension awaits future investigation. Relatively little is known about the initial remodeling of the microtubule cytoskeleton that occurs during dendritic extension in the context of embryogenesis; in the sensory amphid bundles analyzed here, this extension is a multicellular event, the driving force for which is currently not known. The small spatial dimensions involved, together with the tight bundling of dendrites from different neurons in the embryo interior, also limits direct imaging-based analysis of KMN components and microtubules within individual dendrites. While our data are consistent with the idea that the KMN network is utilized to structure and stabilize the microtubule cytoskeleton in developing dendrites, a deeper mechanistic understanding of this process will require the development of a significantly better picture of the complex organizational changes that occur during this event.

Our results show that the KNL-1 and MIS-12 complexes are as important as the NDC-80 complex for sensory nervous system development. During chromosome segregation, the MIS-12 complex and KNL-1 recruit NDC-80 to centromeric chromatin in order to generate a high density of microtubule-binding complexes on the chromosome surface. Within developing neurons, where KMN localization is outside the nucleus, KNL-1 and MIS-12 may still act by recruiting NDC-80 complexes. An important goal of future work will be to define the mechanism(s) that recruit KMN proteins to the dendritic extensions. In addition to its function in NDC-80 recruitment, KNL-1 acts as a scaffold for signaling components that prevent errors during chromosome segregation. It will be intriguing to test if the signaling roles of KNL-1 are also important for the neuronal function of the KMN network.

In addition to defining a new function for ancient chromosome-segregation machinery, our findings suggest new potential reasons for the rapid evolution of kinetochore proteins (e.g., Knl1 in primates (Genin et al., 2012)) and for the existence of human microcephaly mutations in Knl1 (Genin et al., 2012). We suggest that post-mitotic functions of kinetochore components in nervous system development during embryogenesis, rather than solely in chromosome segregation, may contribute to their rapid evolutionary dynamics and to their association with neurodevelopmental disorders in humans.

STAR★METHODS

Detailed methods are provided in the online version of this paper and include the following:

- KEY RESOURCES TABLE
- CONTACT FOR REAGENT AND RESOURCE SHARING
- EXPERIMENTAL MODEL AND SUBJECT DETAILS
- METHOD DETAILS
  - *C. elegans* Transgenic Strain Construction
  - Plasmid Construction
  - Fluorescence Microscopy and Image Analysis
  - Assay for Egg-Laying Defect
  - Fertility Assay
  - Dye-Fill Assay
- QUANTIFICATION AND STATISTICAL ANALYSIS

SUPPLEMENTAL INFORMATION

Supplemental Information includes five figures, two tables, and two videos and can be found with this article online at https://doi.org/10.1016/j.devcel.2019.02.002.

ACKNOWLEDGMENTS

We thank Alex Dammermann for helpful discussions and Kevin Corbett for comments on the manuscript. This work is supported by an NIH grant (GM074215) to A.D.; A.D. and K.O. receive salary and other support from the Ludwig Institute for Cancer Research. D.K.C. is presently supported by Wellcome through a Sir Henry Dale Fellowship (208833) and core funding for Wellcome Centre for Cell Biology (203149).

AUTHOR CONTRIBUTIONS

D.K.C., R.G., and A.D. conceived the study. D.K.C., B.P., and A.D. designed the experiments. D.K.C. and B.P. conducted and analyzed the majority of

experiments. T.C. performed brood size experiments. D.K.C., B.P., N.H., S.W., Z.Z., T.K., and A.G.-G. generated *C. elegans* strains used in the experiments. K.O. and R.G. provided conceptual and experimental advice. D.K.C., B.P., and A.D. prepared the manuscript with input from K.O. and R.G.

DECLARATION OF INTERESTS

The authors declare no competing interests.

Received: July 20, 2018
Revised: November 5, 2018
Accepted: January 31, 2019
Published: February 28, 2019

REFERENCES

Alushin, G.M., Musinipally, V., Matson, D., Tooley, J., Stukenberg, P.T., and Nogales, E. (2012). Multimodal microtubule binding by the Ndc80 kinetochore complex. Nat. Struct. Mol. Biol. *19*, 1161–1167.

Alushin, G.M., Ramey, V.H., Pasqualato, S., Ball, D.A., Grigorieff, N., Musacchio, A., and Nogales, E. (2010). The Ndc80 kinetochore complex forms oligomeric arrays along microtubules. Nature *467*, 805–810.

Bargmann, C.I., Hartwieg, E., and Horvitz, H.R. (1993). Odorant-selective genes and neurons mediate olfaction in C. elegans. Cell *74*, 515–527.

Cabantous, S., Terwilliger, T.C., and Waldo, G.S. (2005). Protein tagging and detection with engineered self-assembling fragments of green fluorescent protein. Nat. Biotechnol. *23*, 102–107.

Carmena, M., Wheelock, M., Funabiki, H., and Earnshaw, W.C. (2012). The chromosomal passenger complex (CPC): from easy rider to the godfather of mitosis. Nat. Rev. Mol. Cell Biol. *13*, 789–803.

Caussinus, E., Kanca, O., and Affolter, M. (2011). Fluorescent fusion protein knockout mediated by anti-GFP nanobody. Nat. Struct. Mol. Biol. *19*, 117–121.

Cheerambathur, D.K., Gassmann, R., Cook, B., Oegema, K., and Desai, A. (2013). Crosstalk between microtubule attachment complexes ensures accurate chromosome segregation. Science *342*, 1239–1242.

Cheerambathur, D.K., Prevo, B., Hattersley, N., Lewellyn, L., Corbett, K.D., Oegema, K., and Desai, A. (2017). Dephosphorylation of the Ndc80 tail stabilizes kinetochore-microtubule attachments via the ska complex. Dev. Cell *41*, 424–437.e4.

Cheeseman, I.M. (2014). The kinetochore. Cold Spring Harb. Perspect. Biol. *6*, a015826.

Cheeseman, I.M., Chappie, J.S., Wilson-Kubalek, E.M., and Desai, A. (2006). The conserved KMN network constitutes the core microtubule-binding site of the kinetochore. Cell *127*, 983–997.

Ciferri, C., Pasqualato, S., Screpanti, E., Varetti, G., Santaguida, S., Dos Reis, G., Maiolica, A., Polka, J., De Luca, J.G., De Wulf, P., et al. (2008). Implications for kinetochore-microtubule attachment from the structure of an engineered Ndc80 complex. Cell *133*, 427–439.

Deluca, J.G., Gall, W.E., Ciferri, C., Cimini, D., Musacchio, A., and Salmon, E.D. (2006). Kinetochore microtubule dynamics and attachment stability are regulated by Hec1. Cell *127*, 969–982.

Dickinson, D.J., Pani, A.M., Heppert, J.K., Higgins, C.D., and Goldstein, B. (2015). Streamlined genome engineering with a self-excising drug selection cassette. Genetics *200*, 1035–1049.

Dickinson, D.J., Ward, J.D., Reiner, D.J., and Goldstein, B. (2013). Engineering the Caenorhabditis elegans genome using Cas9-triggered homologous recombination. Nat. Methods *10*, 1028–1034.

Espeut, J., Cheerambathur, D.K., Krenning, L., Oegema, K., and Desai, A. (2012). Microtubule binding by KNL-1 contributes to spindle checkpoint silencing at the kinetochore. J. Cell Biol. *196*, 469–482.

Frøkjaer-Jensen, C., Davis, M.W., Hopkins, C.E., Newman, B.J., Thummel, J.M., Olesen, S.P., Grunnet, M., and Jorgensen, E.M. (2008). Single-copy insertion of transgenes in Caenorhabditis elegans. Nat. Genet. *40*, 1375–1383.

Genin, A., Desir, J., Lambert, N., Biervliet, M., Van Der Aa, N., Pierquin, G., Killian, A., Tosi, M., Urbina, M., Lefort, A., et al. (2012). Kinetochore KMN network gene CASC5 mutated in primary microcephaly. Hum. Mol. Genet. *21*, 5306–5317.

Gibson, D.G., Young, L., Chuang, R.Y., Venter, J.C., Hutchison, C.A., and Smith, H.O. (2009). Enzymatic assembly of DNA molecules up to several hundred kilobases. Nat. Methods *6*, 343–345.

Heiman, M.G., and Shaham, S. (2009). DEX-1 and DYF-7 establish sensory dendrite length by anchoring dendritic tips during cell migration. Cell *137*, 344–355.

Kamiyama, D., Sekine, S., Barsi-Rhyne, B., Hu, J., Chen, B., Gilbert, L.A., Ishikawa, H., Leonetti, M.D., Marshall, W.F., Weissman, J.S., et al. (2016). Versatile protein tagging in cells with split fluorescent protein. Nat. Commun. *7*, 11046.

Kapitein, L.C., and Hoogenraad, C.C. (2015). Building the neuronal microtubule cytoskeleton. Neuron *87*, 492–506.

Kemp, C.A., Kopish, K.R., Zipperlen, P., Ahringer, J., and O'Connell, K.F. (2004). Centrosome maturation and duplication in C. elegans require the coiled-coil protein SPD-2. Dev. Cell *6*, 511–523.

Koelle, M.R., and Horvitz, H.R. (1996). EGL-10 regulates G protein signaling in the C. elegans nervous system and shares a conserved domain with many mammalian proteins. Cell *84*, 115–125.

Maddox, P.S., Hyndman, F., Monen, J., Oegema, K., and Desai, A. (2007). Functional genomics identifies a Myb domain-containing protein family required for assembly of CENP-A chromatin. J. Cell Biol. *176*, 757–763.

Mangeol, P., Prevo, B., and Peterman, E.J.G. (2016). KymographClear and KymographDirect: two tools for the automated quantitative analysis of molecular and cellular dynamics using kymographs. Mol. Biol. Cell *27*, 1948–1957.

Martino, L., Morchoisne-Bolhy, S., Cheerambathur, D.K., Van Hove, L., Dumont, J., Joly, N., Desai, A., Doye, V., and Pintard, L. (2017). Channel nucleoporins recruit PLK-1 to nuclear pore complexes to direct nuclear envelope breakdown in C. elegans. Dev. Cell *43*, 157–171.e7.

Musacchio, A., and Desai, A. (2017). A molecular view of kinetochore assembly and function. Biology (Basel) *6*, E5.

Nonet, M.L. (1999). Visualization of synaptic specializations in live C. elegans with synaptic vesicle protein-GFP fusions. J. Neurosci. Methods *89*, 33–40.

Oegema, K., Desai, A., Rybina, S., Kirkham, M., and Hyman, A.A. (2001). Functional analysis of kinetochore assembly in Caenorhabditis elegans. J. Cell Biol. *153*, 1209–1226.

Paix, A., Folkmann, A., Rasoloson, D., and Seydoux, G. (2015). High efficiency, homology-directed genome editing in Caenorhabditis elegans using CRISPR-Cas9 ribonucleoprotein complexes. Genetics *201*, 47–54.

Roostalu, J., and Surrey, T. (2017). Microtubule nucleation: beyond the template. Nat. Rev. Mol. Cell Biol. *18*, 702–710.

Schafer, W.F. (2006). Genetics of egg-laying in worms. Annu. Rev. Genet. *40*, 487–509.

Sulston, J.E., Schierenberg, E., White, J.G., and Thomson, J.N. (1983). The embryonic cell lineage of the nematode Caenorhabditis elegans. Dev. Biol. *100*, 64–119.

Waaijers, S., Portegijs, V., Kerver, J., Lemmens, B.B.L.G., Tijsterman, M., van den Heuvel, S., and Boxem, M. (2013). CRISPR/Cas9-targeted mutagenesis in Caenorhabditis elegans. Genetics *195*, 1187–1191.

Wang, S., Tang, N.H., Lara-Gonzalez, P., Zhao, Z., Cheerambathur, D.K., Prevo, B., Chisholm, A.D., Desai, A., and Oegema, K. (2017). A toolkit for GFP-mediated tissue-specific protein degradation in C. elegans. Development *144*, 2694–2701.

Wang, S., Wu, D., Quintin, S., Green, R.A., Cheerambathur, D.K., Ochoa, S.D., Desai, A., and Oegema, K. (2015). NOCA-1 Functions with γ-tubulin and in parallel to Patronin to assemble non-centrosomal microtubule arrays in C. elegans. Elife *4*, e08649.

Ward, S. (1973). Chemotaxis by the nematode Caenorhabditis elegans: identification of attractants and analysis of the response by use of mutants. Proc. Natl. Acad. Sci. U S A *70*, 817–821.

Winkelbauer, M.E., Schafer, J.C., Haycraft, C.J., Swoboda, P., and Yoder, B.K. (2005). The C. elegans homologs of nephrocystin-1 and nephrocystin-4 are cilia transition zone proteins involved in chemosensory perception. J. Cell. Sci. *118*, 5575–5587.

Yan, J., Chao, D.L., Toba, S., Koyasako, K., Yasunaga, T., Hirotsune, S., and Shen, K. (2013). Kinesin-1 regulates dendrite microtubule polarity in Caenorhabditis elegans. Elife *2*, e00133.

Zhao, G., Oztan, A., Ye, Y., and Schwarz, T.L. (2019). Kinetochore proteins have a post-mitotic function in neurodevelopment. Dev. Cell *48*, this issue, 873–882.

STAR★METHODS

KEY RESOURCES TABLE

REAGENT or RESOURCE	SOURCE	IDENTIFIER
Experimental Model: *C. elegans* Organisms/Strains		
C. elegans N2 Bristol	Caenorhabditis Genetics Center	N2
ltSi1[pOD809/pJE110; Pknl-1::KNL-1reencoded::mCherry; cb-unc-119(+)] II;unc-119(ed3) III	Espeut et al., 2012, PMID: 22331849	OD334
ltSi120[[pDC170;Pndc-80:NDC-80 reencoded; cb-unc-119(+)]II; unc-119 (ed3)III	Cheerambathur et al. 2013, PMID: 24231804,	OD611
ltSi121[pDC175;Pndc-80:NDC-80 (Mutant D1-59) reencoded; cb-unc-119(+)]II; unc-119(ed3)III	Cheerambathur et al., 2013, PMID: 24231804	OD612
ltSi272[pOD1272/pSW094; Phlh-1::mCherry::his-72::unc-54_3'UTR; Ppha-4::mCherry::his-72::tbb-2_3'UTR; cb-unc-119(+)]I; unc-119(ed3)III	This study	OD1006
ltSi511[pOD1498/pSW207; Pcnd-1::mCherry-PH::unc-54_3'UTR; cb-unc-119(+)]II	This study	OD1593
ltSi711[pDC267;Pndc-80:NDC-80(66,96,100,125,144,155AAAAAA) reencoded; cb-unc-119(+)]II#1; unc-119(ed3)III	Cheerambathur et al., 2017, PMID: 28535376	OD2412
ska-1(lt28[ska-1::GFP]::loxp)I	Cheerambathur et al., 2017, PMID: 28535376	OD692
plk-1(lt18[plk-1::sGFP]::loxp)	Martino et al., 2017, PMID: 29065307	OD2425
gip-2(lt19[gip-2::GFP]::loxP::cb-unc-119(+)::loxP)I; unc-119(ed3)III	Wang et al., 2015, PMID: 26371552	OD2509
ltSi910[pOD2044/pSW378; Pelt-2::vhhGFP4::ZIF-1::operon-linker:: mCherry::histone::tbb-2_3'UTR; cb-unc-119(+)]II; unc-119(ed3)III	Wang et al., 2015, PMID: 26371552	OD2768
mdf-1(lt39[GFP::tev::loxP::3xFlag::mdf-1])V	Wang et al., 2015, PMID: 26371552	OD2906
him-10(lt52[him-10::GFP]) III	This Study	OD2953
ltSi1016[pDC337; Pdyf-7::vhhGFP4::ZIF-1::dyf-7_3'UTR; cb-unc-119(+)] I #2 ; unc-119(ed3)III	This Study	OD3025
knl-1(lt53[knl-1::GFP::tev::loxP::3xFlag]))III	This Study	OD3026
ndc-80(lt54[ndc-80::GFP::tev::loxP::3xFlag])IV	This Study	OD3029
knl-3 (lt46 [GFP::knl-3]) V	This Study	OD3101
ltSi1033 [pDC352; Pdyf-7::mCherry::PH_unc-54_3'UTR; cb-unc-119(+)]II ; unc-119(ed3)III	This Study	OD3152
ltSi1016[pDC337; Pdyf-7::vhhGFP4::ZIF-1::dyf-7_3'UTR; cb-unc-119(+)]I] #2; unc-119(ed3)III? ; knl-3[lt46(GFP::knl-3)]V	This Study	OD3174
air-2(lt58[air-2::GFP::tev::loxP::3xFlag]) I	This Study	OD3230
ltSi1038 [pDC344; Pgcy-5::mKate-2::PH_unc-54_3'UTR; cb-unc-119(+)]II ; unc-119(ed3)III	This Study	OD3242
dyf-7 (lt60)X	This Study	OD3244
ltSi1016[pDC337; Pdyf-7::vhhGFP4::ZIF-1::dyf-7_3'UTR; cb-unc-119(+)]I #2; unc-119(ed3)III?; knl-1(lt53[knl-1::GFP::tev::loxP::3xFlag) IV	This Study	OD3245
ltSi1016[pDC337; Pdyf-7::vhhGFP4::ZIF-1::dyf-7_3'UTR; cb-unc-119(+)]I #2; ltSi1038 [pDC344; Pgcy-5::mKate-2::PH_unc-54_3'UTR; cb-unc-119(+)]II; unc-119(ed3)III; unc-119(ed3)III?; knl-1(lt53[knl-1::GFP::tev::loxP::3xFlag) IV	This Study	OD3247
ltSi1016[pDC337; Pdyf-7::vhhGFP4::ZIF-1::dyf-7_3'UTR; cb-unc-119(+)]I #2; ltSi1038 [pDC344; Pgcy-5::mKate-2::PH_unc-54_3'UTR; cb-unc-119(+)]II; unc-119(ed3)III ?	This Study	OD3249
ltSi1041 [pDC376; Pdyf-7::ebp-2::mNeonG_tbb-2_3'UTR::operon-linker:: mCherry::PH_unc-54_3'UTR; cb-unc-119(+)]II; unc-119(ed3)III	This Study	OD3252

(*Continued on next page*)

Continued

REAGENT or RESOURCE	SOURCE	IDENTIFIER
ltSi1016[pDC337; Pdyf-7::vhhGFP4::ZIF-1::dyf-7_3'UTR; cb-unc-119(+)]I] #2; unc-119(ed3)III? ; ndc-80(lt54[ndc-80::GFP::tev::loxP::3xFlag])IV	This Study	OD3258
ltSi1016[pDC337; Pdyf-7::vhhGFP4::ZIF-1::dyf-7_3'UTR]#2 I; ltSi1041 [pDC376; Pdyf-7::ebp-2::mNeonG_tbb-2_3'UTR::operon-linker:: mCherry::PH_unc-54_3'UTR; cb-unc-119(+)]II] ; unc-119(ed3)III?	This Study	OD3275
ltSi1016[pDC337; Pdyf-7::vhhGFP4::ZIF-1::dyf-7_3'UTR]#2 I; ltSi1041 [pDC376; Pdyf-7::ebp-2::mNeonG_tbb-2_3'UTR::operon-linker:: mCherry::PH_unc-54_3'UTR; cb-unc-119(+)]II] ; knl-1(lt53[knl-1::GFP:: tev::loxP::3xFlag]) III	This Study	OD3276
ltSi1016[pDC337; Pdyf-7::vhhGFP4::ZIF-1::dyf-7_3'UTR; cb-unc-119(+) knl-1(lt53[knl-1::GFP::tev::loxP::3xFlag]) IIIltSi1016[pDC337; Pdyf-7:: vhhGFP4::ZIF-1::dyf-7_3'UTR; cb-unc-119(+)]#2 I; ltSi1038 [pDC344; Pgcy-5::mKate-2::PH_unc-54_3'UTR; cb-unc-119(+)]II; unc-119(ed3)III? ; ndc-80(lt54[ndc-80::GFP::tev::loxP::3xFlag])IV	This Study	OD3281
ltSi1033 [pDC352; Pdyf-7::mCherry::PH_unc-54_3'UTR; cb-unc-119(+)]II ; unc-119(ed3)III?; knl-3[lt46(GFP::knl-3)]V	This Study	OD3295
ltSi1045[pDC378; Pdyf-7::mNeonG::PH_tbb-2_3'UTR::operon-linker:: mCherry::his-72_unc-54_3'UTR; cb-unc-119(+)]II; unc-119(ed3)III	This Study	OD3301
ltSi1016[pDC337; Pdyf-7::vhhGFP4::ZIF-1::dyf-7_3'UTR; cb-unc-119(+)]I ; ltSi1045[pDC378; Pdyf-7::mNeonG::PH_tbb-2_3'UTR::operon-linker::mCherry::his-72_unc-54_3'UTR; cb-unc-119(+)]II; unc-119(ed3)III?	This Study	OD3319
ltSi1016[pDC337; Pdyf-7::vhhGFP4::ZIF-1::dyf-7_3'UTR; cb-unc-119(+)]I ; ltSi1045[pDC378; Pdyf-7::mNeonG::PH_tbb-2_3'UTR::operon-linker:: mCherry::his-72_unc-54_3'UTR; cb-unc-119(+)]II; knl-1(lt53[knl-1::GFP:: tev::loxP::3xFlag]) III	This Study	OD3320
ltSi1016[pDC337; Pdyf-7::vhhGFP4::ZIF-1::dyf-7_3'UTR; cb-unc-119(+)]#2 I] ; ltSi1[pOD809/pJE110; Pknl-1::KNL-1reencoded::mCherry; cb-unc-119(+)]II; knl-1(lt53[knl-1::GFP::tev::loxP::3xFlag]) III	This Study	OD3346
ltSi1016[pDC337; Pdyf-7::vhhGFP4::ZIF-1::dyf-7_3'UTR; cb-unc-119(+)]I ; ltSi1045[pDC378; Pdyf-7::mNeonG::PH_tbb-2_3'UTR::operon-linker:: mCherry::his-72_unc-54_3'UTR; cb-unc-119(+)]II;unc-119(ed3)III?; ndc-80(lt54[ndc-80::GFP::tev::loxP::3xFlag])IV	This Study	OD3348
ltSi1016[pDC337; Pdyf-7::vhhGFP4::ZIF-1::dyf-7_3'UTR; cb-unc-119(+)]I ; ltSi120[[pDC170;Pndc-80:NDC-80 reencoded; cb-unc-119(+)]II #3; unc-119(ed3)III? ; ndc-80(lt54[ndc-80::GFP::tev::loxP::3xFlag])IV	This Study	OD3355
ltSi1016[pDC337; Pdyf-7::vhhGFP4::ZIF-1::dyf-7_3'UTR; cb-unc-119(+)]I ; unc-119(ed3)III; ltSi711[pDC267;Pndc-80:NDC-80(66,96,100,125,144, 155AAAAAA) reencoded; cb-unc-119(+)]II#1; unc-119(ed3)III? ; ndc-80 (lt54[ndc-80::GFP::tev::loxP::3xFlag])IV	This Study	OD3356
ltSi1050 [pDC389; Pdyf-7::mCH::his-72_unc-54_3'UTR; cb-unc-119(+)]II; unc-119(ed3)III	This Study	OD3358
ltSi1052 [pDC392; Pelt-3::mCH::his-72_unc-54_3'UTR; cb-unc-119(+)]II; unc-119(ed3)III	This Study	OD3361
rod-1(lt62[GFP::rod-1) IV	This Study	OD3367
ltSi272[pOD1272/pSW094; Phlh-1::mCherry::his-72::unc-54_3'UTR; Ppha-4::mCherry::his-72::tbb-2_3'UTR; cb-unc-119(+)]I; unc-119(ed3)III?; knl-3 [lt46(gfp::knl-3)]V	This Study	OD3372
ltSi511[pOD1498/pSW207; Pcnd-1::mCherry-PH::unc-54_3'UTR; cb-unc-119(+)]II; unc-119(ed3)III?; knl-3[lt46(GFP::knl-3)]V	This Study	OD3373
ltSi1050 [pDC389; Pdyf-7::mCH::his-72_unc-54_3'UTR; cb-unc-119(+)]II; unc-119(ed3)III?; knl-3[lt46(GFP::knl-3)]V	This Study	OD3398
ltSi1052 [pDC392; Pelt-3::mCH::his-72_unc-54_3'UTR; cb-unc-119(+)]II; unc-119(ed3)III?; knl-3[lt46(gfp::knl-3)]V	This Study	OD3399
knl-1(it75[knl-1::mCherry]) III	This Study	OD3392

(*Continued on next page*)

Continued

REAGENT or RESOURCE	SOURCE	IDENTIFIER
ltSi1016[pDC337; Pdyf-7::vhhGFP4::ZIF-1::dyf-7_3'UTR; cb-unc-119(+)]I ; ltSi1045[pDC378; Pdyf-7::mNeonG::PH_tbb-2_3'UTR::operon-linker:: mCherry::his-72_unc-54_3'UTR; cb-unc-119(+)]II;unc-119(ed3)III?; knl-3 [lt46(GFP::knl-3)]V	This Study	OD3401
knl-2(lt73[GFP::knl-2])I	This Study	OD3407
ltSi1016[pDC337; Pdyf-7::vhhGFP4::ZIF-1::dyf-7_3'UTR; cb-unc-119(+)]I ; ltSi120[[pDC170;Pndc-80:NDC-80 reencoded; cb-unc-119(+)]II #3; unc-119(ed3)III? ; ndc-80(lt54[ndc-80::GFP::tev::loxP::3xFlag])IV; ltSi1054 [oxTi365;pDC378; Pdyf-7::mNeonG::PH_tbb-2_3'UTR::operon-linker:: mCherry::his-72_unc-54_3'UTR; cb-unc-119(+)] V	This Study	OD3408
hcp-4(lt72[GFP::hcp-4])I	This Study	OD3410
ltSi1016[pDC337; Pdyf-7::vhhGFP4::ZIF-1::dyf-7_3'UTR; cb-unc-119(+)]I; unc-119(ed3)III; ltSi121[pDC175;Pndc-80:NDC-80 (Mutant D1-59) reencoded; cb-unc-119(+)]II #1; unc-119(ed3)III? ; ndc-80(lt54[ndc-80::GFP::tev::loxP:: 3xFlag])IV; ltSi1054[oxTi365;pDC378; Pdyf-7::mNeonG::PH_tbb-2_3'UTR:: operon-linker::mCherry::his-72_unc-54_3'UTR; cb-unc-119(+)] V	This Study	OD3412
ltSi1016[pDC337; Pdyf-7::vhhGFP4::ZIF-1::dyf-7_3'UTR; cb-unc-119(+)]I ; unc-119(ed3)III; ltSi711[pDC267;Pndc-80:NDC-80(66,96,100,125,144, 155AAAAAA) reencoded; cb-unc-119(+)]II#1; unc-119(ed3)III? ; ndc-80(lt54 [ndc-80::GFP::tev::loxP::3xFlag])IV; ltSi1054[oxTi365;pDC378; Pdyf-7:: mNeonG::PH_tbb-2_3'UTR::operon-linker::mCherry::his-72_unc-54_3'UTR; cb-unc-119(+)] V	This Study	OD3413
ltSi1016[pDC337; Pdyf-7::vhhGFP4::ZIF-1::dyf-7_3'UTR; cb-unc-119(+)]#2 I] ; ltSi1[pOD809/pJE110; Pknl-1::KNL-1reencoded::RFP; cb-unc-119(+)]II; knl-1(lt53[knl-1::GFP::tev::loxP::3xFlag]) III; ltSi1054[oxTi365;pDC378; Pdyf-7:: mNeonG::PH_tbb-2_3'UTR::operon-linker::mCherry::his-72_unc-54_3'UTR; cb-unc-119(+)] V	This Study	OD3431
spd-2(lt76[gfp::spd-2]) I	This Study	OD3453
hcp-3(lt78[GFP::hcp-3])I	This Study	OD3463
bub-1(lt82 [bub-1::GFP])I	This Study	OD3516
ltSi1168[pDC589;Pdyf-7::ndc-80 reencoded-::mCherry::dyf_3'UTR; cb-unc-119(+)]II; unc-119(ed3)III	This Study	OD3915
unc-119(ed3)III; ltSi1174[oxTi365; pDC591; Pnphp-4::mNeonGreen-his-72: tbb-2_3'UTR;;gpd-2/3 operon linker-mKate2-PH:unc-34_3'UTR]V	This Study	OD3919
ltSi1169[pDC588;Pdyf-7::knl-1 reencoded-::mCherry::dyf_3'UTR; cb-unc-119(+)]II; unc-119(ed3)III	This Study	OD3920
ltSi1175[pDC585; Pcnd-1::mCH::his-11_unc-54_3'UTR; cb-unc-119(+)]II; unc-119(ed3)III	This Study	OD3921
ltSi1016[pDC337; Pdyf-7::vhhGFP4::ZIF-1::dyf-7_3'UTR; cb-unc-119(+)]I; unc-119(ed3)III?; ltSi1174[oxTi365; pDC591; Pnphp-4::mNeonGreen-his-72:tbb-2_3'UTR;;gpd-2/3 operon linker-mKate2-PH:unc-34_3'UTR]V	This Study	OD3924
ltSi1175[pDC585; Pcnd-1::mCH::his-11_unc-54_3'UTR; cb-unc-119(+)]II; unc-119(ed3)III?; knl-3[lt46(gfp::knl-3)]V	This Study	OD3927
ltSi1016[pDC337; Pdyf-7::vhhGFP4::ZIF-1::dyf-7_3'UTR; cb-unc-119(+)] I] ; knl-1(lt53[knl-1::GFP::tev::loxP::3xFlag]) III; ltSi1174[oxTi365; pDC591; Pnphp-4::mNeonGreen-his-72:tbb-2_3'UTR;;gpd-2/3 operon linker-mKate2-PH:unc-34_3'UTR]V	This tudy	OD3938
ltSi910[pOD2044/pSW378; Pelt-2::vhhGFP4::ZIF-1::operon-linker::mCherry:: histone::tbb-2_3'UTR; cb-unc-119(+)]II;knl-1(lt53[knl-1::GFP::tev::loxP:: 3xFlag])III	This Study	OD3931
ltSi910[pOD2044/pSW378; Pelt-2::vhhGFP4::ZIF-1::operon-linker::mCherry:: histone::tbb-2_3'UTR; cb-unc-119(+)]II; unc-119(ed3)III?;ndc-80(lt54[ndc-80::GFP::tev::loxP::3xFlag])IV	This Study	OD3932
ltSi910[pOD2044/pSW378; Pelt-2::vhhGFP4::ZIF-1::operon-linker::mCherry:: histone::tbb-2_3'UTR; cb-unc-119(+)]II; unc-119(ed3)III?;knl-3 (lt46 [GFP:: knl-3]) V	This Study	OD3933

(*Continued on next page*)

Continued

REAGENT or RESOURCE	SOURCE	IDENTIFIER
spd-2(lt76[gfp::spd-2])I; ltSi910[pOD2044/pSW378; Pelt-2::vhhGFP4::ZIF-1::operon-linker::mCherry::histone::tbb-2_3'UTR; cb-unc-119(+)]II; unc-119(ed3)III	This Study	OD3934
knl-2(lt73[gfp::knl-2])I; ltSi910[pOD2044/pSW378; Pelt-2::vhhGFP4::ZIF-1::operon-linker::mCherry::histone::tbb-2_3'UTR; cb-unc-119(+)]II; unc-119(ed3)III?	This Study	OD3936
ltSi1016[pDC337; Pdyf-7::vhhGFP4::ZIF-1::dyf-7_3'UTR; cb-unc-119(+)]I ; unc-119(ed3)III? ;Pnphp-4::mNeonGreen-his-72:tbb-2_3'UTR;;gpd-2/3 operon linker-mKate2-PH:unc-34_3'UTR] knl-3[lt46(GFP::knl-3)]V	This Study	OD3948
ltSi1016[pDC337; Pdyf-7::vhhGFP4::ZIF-1::dyf-7_3'UTR; cb-unc-119(+)] gip-2(lt19[gip-2::GFP]::loxP::cb-unc-119(+)::loxP)I ; ltSi1045[pDC378; Pdyf-7::mNeonG::PH_tbb-2_3'UTR::operon-linker::mCherry::his-72_unc-54_3'UTR; cb-unc-119(+)]II;unc-119(ed3)III?	This Study	OD3951
ltSi1016[pDC337; Pdyf-7::vhhGFP4::ZIF-1::dyf-7_3'UTR; cb-unc-119(+)]I ; ltSi120[[pDC170;Pndc-80:NDC-80 reencoded; cb-unc-119(+)]II #3; unc-119(ed3)III? ; ndc-80(lt54[ndc-80::GFP::tev::loxP::3xFlag])IV; Pnphp-4::mNeonGreen-his-72:tbb-2_3'UTR;;gpd-2/3 operon linker-mKate2-PH:unc-34_3'UTR]V	This Study	OD3952
ltSi1016[pDC337; Pdyf-7::vhhGFP4::ZIF-1::dyf-7_3'UTR; cb-unc-119(+)]I ; unc-119(ed3)III; ltSi121[pDC175;Pndc-80:NDC-80 (Mutant D1-59) reencoded; cb-unc-119(+)]II #1; unc-119(ed3)III? ; ndc-80(lt54[ndc-80::GFP::tev::loxP::3xFlag])IV; Pnphp-4::mNeonGreen-his-72:tbb-2_3'UTR;;gpd-2/3 operon linker-mKate2-PH:unc-34_3'UTR]V	This Study	OD3953
ltSi1016[pDC337; Pdyf-7::vhhGFP4::ZIF-1::dyf-7_3'UTR; cb-unc-119(+)]I ; unc-119(ed3)III; ltSi711[pDC267;Pndc-80:NDC-80(66,96,100,125,144,155AAAAAA) reencoded; cb-unc-119(+)]II#1; unc-119(ed3)III? ; ndc-80(lt54[ndc-80::GFP::tev::loxP::3xFlag])IV; ltSi1055[oxTi365;[pDC344; Pnphp-4::mNeonGreen-his-72:tbb-2_3'UTR;;gpd-2/3 operon linker-mKate2-PH:unc-34_3'UTR]V	This Study	OD3954
ltSi1016[pDC337; Pdyf-7::vhhGFP4::ZIF-1::dyf-7_3'UTR; cb-unc-119(+)]I; unc-119(ed3)III?; ltSi1174[oxTi365; pDC591; Pnphp-4::mNeonGreen-his-72:tbb-2_3'UTR;;gpd-2/3 operon linker-mKate2-PH:unc-34_3'UTR]V; dyf-7 (it60)X	This Study	OD3965
ltSi1016[pDC337; Pdyf-7::vhhGFP4::ZIF-1::dyf-7_3'UTR; cb-unc-119(+)] knl-2(lt73[GFP::knl-2])I; unc-119(ed3)III?; ltSi1174[oxTi365; pDC591; Pnphp-4::mNeonGreen-his-72:tbb-2_3'UTR;;gpd-2/3 operon linker-mKate2-PH:unc-34_3'UTR]V	This Study	OD3971
unc-119(ed3) III; ltSi1181[oxTi365; pDC559; Pdyf-7:GFP-tbb-2_dyf-7_3'UTR; cb-unc-119(+)]V	This Study	OD3974
ltSi1184[pDC593; Pdyf-7::sGFPS1-10::tbb-2_3'UTR::gpd-2/3 operon linker::mCherry-his-15::unc-54-3'UTR;; cb-unc-119(+)] II; unc-119(ed3) III	This Study	OD3976
ltSi1016[pDC337; Pdyf-7::vhhGFP4::ZIF-1::dyf-7_3'UTR; cb-unc-119(+)] I] ; ltSi1[pOD809/pJE110; Pknl-1::KNL-1reencoded::RFP; cb-unc-119(+)]II; knl-1(lt53[knl-1::GFP::tev::loxP::3xFlag]) III; ltSi1174[oxTi365; pDC591; Pnphp-4::mNeonGreen-his-72:tbb-2_3'UTR;;gpd-2/3 operon linker-mKate2-PH:unc-34_3'UTR]V	This Study	OD3977
ndc-80[lt126 (ndc-80::7XGFP-11)] IV	This Study	OD3995
ltSi1184[pDC593; Pdyf-7::sGFPS1-10::tbb-2_3'UTR::gpd-2/3 operon linker::mCherry-his-15::unc-54-3'UTR;; cb-unc-119(+)] II; unc-119(ed3) III?; ndc-80[lt126 (ndc-80::7XGFP-11)] IV	This Study	OD3997
ltSi1016[pDC337; Pdyf-7::vhhGFP4::ZIF-1::dyf-7_3'UTR; cb-unc-119(+)]I ; unc-119(ed3)III? ; ndc-80(lt54[ndc-80::GFP::tev::loxP::3xFlag])IV; ltSi1174 [oxTi365; pDC591; Pnphp-4::mNeonGreen-his-72:tbb-2_3'UTR;;gpd-2/3 operon linker-mKate2-PH:unc-34_3'UTR]V	This Study	OD3998
ltSi1191[pDC603; Pgcy-5::mNeonGreen-PH::tbb-3'UTR; Pgcy-5::mKate2-snb-1::snb-1_3'UTR; cb-unc-119(+)] II; unc-119(ed3) III	This Study	OD4000

(*Continued on next page*)

Continued

REAGENT or RESOURCE	SOURCE	IDENTIFIER
ltSi1016[pDC337; Pdyf-7::vhhGFP4::ZIF-1::dyf-7_3'UTR; cb-unc-119(+)]I ; ltSi1191[pDC603; Pgcy-5::mNeonGreen-PH::tbb-3'UTR; Pgcy-5::mKate2-snb-1::snb-1_3'UTR; cb-unc-119(+)] II; unc-119(ed3) III?	This Study	OD4011
ltSi1016[pDC337; Pdyf-7::vhhGFP4::ZIF-1::dyf-7_3'UTR; cb-unc-119(+)] I] ; ltSi1191[pDC603; Pgcy-5::mNeonGreen-PH::tbb-3'UTR; Pgcy-5::mKate2-snb-1::snb-1_3'UTR; cb-unc-119(+)] II; knl-1(lt53[knl-1::GFP::tev::loxP::3xFlag]) III	This Study	OD4012
ltSi1016[pDC337; Pdyf-7::vhhGFP4::ZIF-1::dyf-7_3'UTR; cb-unc-119(+)]I ; unc-119(ed3)III? ;ltSi1191[pDC603; Pgcy-5::mNeonGreen-PH::tbb-3'UTR; Pgcy-5::mKate2-snb-1::snb-1_3'UTR; cb-unc-119(+)] II; unc-119(ed3) III?; ndc-80(lt54[ndc-80::GFP::tev::loxP::3xFlag])IV	This Study	OD4013
ltSi1016[pDC337; Pdyf-7::vhhGFP4::ZIF-1::dyf-7_3'UTR; cb-unc-119(+)] spd-2(lt76[GFP::spd-2]) I; unc-119(ed3)III?; ltSi1174[oxTi365; pDC591; Pnphp-4::mNeonGreen-his-72:tbb-2_3'UTR;;gpd-2/3 operon linker-mKate2-PH:unc-34_3'UTR]V	This Study	OD4014
ltSi1186[pDC599; Pcnd-1::sGFPS1-10::tbb-2_3'UTR::gpd-2/3 operon linker::mCherry-his-15::unc-54-3'UTR;; cb-unc-119(+)] II; unc-119(ed3) III	This Study	OD4015
ltSi1016[pDC337; Pdyf-7::vhhGFP4::ZIF-1::dyf-7_3'UTR; cb-unc-119(+)] I]; ltSi1169[pDC588;Pdyf-7::knl-1 reencoded-::mCherry::dyf_3'UTR; cb-unc-119(+)]II; knl-1(lt53[knl-1::GFP::tev::loxP::3xFlag]) III; ltSi1174[oxTi365; pDC591; Pnphp-4::mNeonGreen-his-72:tbb-2_3'UTR;;gpd-2/3 operon linker-mKate2-PH:unc-34_3'UTR]V	This Study	OD4025
ltSi1016[pDC337; Pdyf-7::vhhGFP4::ZIF-1::dyf-7_3'UTR; cb-unc-119(+)]I ; ltSi1168[pDC589;Pdyf-7::ndc-80 reencoded-::mCherry::dyf_3'UTR; cb-unc-119(+)]II; unc-119(ed3)III? ; ndc-80(lt54[ndc-80::GFP::tev::loxP::3xFlag]) IV; ltSi1174[oxTi365; pDC591; Pnphp-4::mNeonGreen-his-72:tbb-2_3'UTR;; gpd-2/3 operon linker-mKate2-PH:unc-34_3'UTR]V	This Study	OD4026
him-10[lt130(him-10::mScarlet)] III; unc-119(ed3) III?; ltSi1181[oxTi365; pDC559; Pdyf-7:GFP-tbb-2_dyf-7_3'UTR; cb-unc-119(+)]V	This Study	OD4038
ltSi1186[pDC599; Pcnd-1::sGFPS1-10::tbb-2_3'UTR::gpd-2/3 operon linker::mCherry-his-15::unc-54-3'UTR;; cb-unc-119(+)] II; unc-119(ed3) III?; ndc-80[lt126 (ndc-80::7XGFP-11)] IV	This Study	OD4039
him-10[lt130(him-10::mScarlet)] III	This Study	OD4040
gip-2(lt19[gip-2::GFP]::loxP::cb-unc-119(+)::loxP)I; unc-119(ed3)III?; him-10[lt130(him-10::mScarlet)] III	This Study	OD4107
ltSi1041 [pDC376; Pdyf-7::ebp-2::mNeonG_tbb-2_3'UTR::operon-linker::mCherry::PH_unc-54_3'UTR; cb-unc-119(+)]II ; unc-119(ed3)III?; him-10 [lt130(him-10::mScarlet)] III	This Study	OD4109
ltSi1016[pDC337; Pdyf-7::vhhGFP4::ZIF-1::dyf-7_3'UTR; cb-unc-119(+)]I]; knl-1(it75[knl-1::mCherry]) III	This Study	DKC05
ltSi1016[pDC337; Pdyf-7::vhhGFP4::ZIF-1::dyf-7_3'UTR; cb-unc-119(+)]I]; knl-1(it75[knl-1::mCherry]) III;ndc-80(lt54[ndc-80::GFP::tev::loxP::3xFlag])IV	This Study	DKC06
ltSi1016[pDC337; Pdyf-7::vhhGFP4::ZIF-1::dyf-7_3'UTR; cb-unc-119(+)]I]; ltSi120[[pDC170;Pndc-80:NDC-80 reencoded; cb-unc-119(+)]II #3; knl-1 (it75[knl-1::mCherry]) III;ndc-80(lt54[ndc-80::GFP::tev::loxP::3xFlag])IV	This Study	DKC07
ltSi1016[pDC337; Pdyf-7::vhhGFP4::ZIF-1::dyf-7_3'UTR; cb-unc-119(+)]I]; ltSi711[pDC267;Pndc-80:NDC-80(66,96,100,125,144,155AAAAAA) reencoded; cb-unc-119(+)]II#1; knl-1(it75[knl-1::mCherry]) III;ndc-80(lt54 [ndc-80::GFP::tev::loxP::3xFlag])IV	This Study	DKC08
Software and Algorithms		
Image J (Fiji)	N/A	http://rsbweb.nih.gov/ij/
GraphPad Prism	N/A	http://www.graphpad.com/
Pymol 1.8	N/A	https://www.pymol.org

(*Continued on next page*)

Continued		
REAGENT or RESOURCE	SOURCE	IDENTIFIER
Other		
Andor Technology System – Yokogawa Spinning Disk Unit	Andor / Nikon	N/A
Deltavision Elite – Applied Precision / PCO	GE Healthcare / Olympus	N/A
Zeiss LSM 880 AxioObserver System	Zeiss	N/A
Zeiss Z1 AxioObserver System – Yokogawa Spinning Disk Unit	Zeiss / Photometrics	N/A

CONTACT FOR REAGENT AND RESOURCE SHARING

Further information and requests regarding reagents should be directed to and will be fulfilled by the Lead Contact, Arshad Desai (abdesai@ucsd.edu).

EXPERIMENTAL MODEL AND SUBJECT DETAILS

All *C. elegans* strains were maintained at 20°C on standard Nematode Growth Media (NGM) plates seeded with OP50 bacteria. The genotypes of the C. elegans strains used in this study are described in *Reagents and Resources*.

METHOD DETAILS

C. elegans Transgenic Strain Construction

Single copy transgenic integrations were engineered using the transposon based mos1 mediated Single Copy Insertion (mosSCI) method (Frøkjaer-Jensen et al., 2008). Briefly, the strains were generated by injecting a mixture of repair plasmid containing the transgene and a positive selection marker, transposase plasmid, and the three plasmids encoding fluorescent markers for negative selection (Frøkjaer-Jensen et al., 2008) [pCFJ90 (P*myo-2::mCherry*), pCFJ104 (P*myo-3::mCherry*) and pGH8 (P*rab-3::mCherry*)] into appropriate *C. elegans* strains that contain mos1 insertion sites at specific genomic locations within Chr I, II or V. Positive integrants were identified by selecting worms that were moving and did not contain fluorescent selection markers. Integration of transgenes were confirmed by PCR, spanning both homology arms. Endogenous tagging of various genes (see Table S1) at the N- or C-teminus with GFP or mScarlet and generation of the *dyf-7* deletion were done using CRISPR/Cas9 methods (Dickinson et al., 2015; Paix et al., 2015; Waaijers et al., 2013). The specific method and guide RNAs used to generate each strain are described in Table S1. Briefly, for the generation of GFP or mScarlet fusions, the repair template (consisting of ~1500bp flanking either GFP or mScarlet) was co-injected into wildtype N2 animals with plasmids containing Cas9 (pDD162 (Dickinson et al., 2013)), the respective guide RNA sequences, and the three plasmids encoding fluorescent markers for negative selection (Frøkjaer-Jensen et al., 2008) [pCFJ90 (P*myo-2::mCherry*), pCFJ104 (P*myo-3::mCherry*) and pGH8 (P*rab-3::mCherry*)]. Recombinant strains were identified by appropriate selection method and were confirmed by PCR, spanning both homology regions.

Plasmid Construction

All plasmids were constructed using the Gibson Assembly method (Gibson et al., 2009). The cloning strategy for the GFP nanobody fragment (vhhGFP4) fused to ZIF-1 genomic DNA, and the regulatory elements for *ndc-80* and *knl-1* single copy transgene insertion, under endogenous promoter, have been previously described (Cheerambathur et al., 2013; Espeut et al., 2012; Wang et al., 2017). The regulatory sequences used for the generation of all other strains, and the codon-optimized sequences for Split GFP and mScarlet are in Table S2.

Fluorescence Microscopy and Image Analysis

Live embryo imaging experiments were performed using an Andor Technology confocal imaging system (described below), unless noted otherwise. The Andor Revolution XD Confocal System was equipped with a Yokogawa spinning disk unit (CSU-10, Yokogawa Corporation of America) that was mounted on a Nikon inverted microscope (TE2000-E, Nikon) housing a 100X 1.4 NA Plan Apochromat (Nikon) objective. Two solid-state 100 mW lasers provided illumination and a back-illuminated EMCCD camera (iXon DV887; Andor Technology) was used for detection. Embryos in Figure S5D were imaged using a Zeiss LSM 880 AxioObserver with a 1.46 NA 100X Alpha Plan Apochromat (Zeiss) objective. A 561 nm HeNe laser provided illumination and emission light was detected using a GaAsp detector. 8 x 0.5 μm z-stacks, containing 904 pixel by 904 pixel images having a spatial resolution of 94 nm/pixel, were acquired and projected. For imaging, embryos were mounted in M9 on a 2% agarose pad, which was subsequently covered with a 22 x 22 mm coverslip and sealed with VaLaP (1:1:1 vasoline:lanolin:paraffin). Z-stacks were acquired to cover approximately half the embryo depth from the side facing the coverslip / objective.

To quantify neurite extension, 11 x 0.5 μm z-stacks were acquired at 1 min intervals and 100 ms exposure. Z-stacks were projected and bundle extension was measured using a segmented line (spline fit) drawn over the top of the amphid bundle extending from the tip to a reference point at the back of the cellular complex using Image J (Fiji).

To quantify protein degradation, 21 x 0.5 μm z-stacks were acquired at an exposure of 100 ms (for OD3029 and OD3258, see Key Resources Table) or at 200 ms (for OD3026 and OD3245, see Key Resources Table). Z-stacks were projected and the integrated fluorescence intensity was measured in the anterior part of the head (comma stage embryos), using Image J (Fiji), while applying an intensity threshold of 10000 and excluding any signal arising from kinetochore localization.

To image EBP-2 dynamics, 5 x 0.75 μm z-stacks were acquired at 0.8 sec intervals and 50 ms exposure. Z-stacks were projected and kymographs were generated using the KymographDirect (Mangeol et al., 2016) Image J/Fiji plugin. EBP-2 velocities were extracted from the kymographs manually.

To image sensory neurons expressing P*nphp-4* driven mKate-PH and mNeonGreen histone, L1 stage worms were anesthetized in 5mM Levamisole and mounted in M9 on a 2% agarose pad. 30 x 0.5 μm z-stacks were acquired using an inverted Zeiss Axio Observer Z1 system with a Yokogawa spinning-disk confocal head (CSU-X1), a 63X 1.4 NA Plan Apochromat objective (Zeiss, Oberkochen, Germany), and an EMCCD camera (QuantEM:512SC, Photometrics, Tucson, AZ). Maximum intensity projections of z-stacks were made using Image J (Fiji) and the cell body distribution width was measured as the distance between the most anterior and most posterior cell body within the head region. To image the ASER neuron expressing mKate-PH, synchronized adult worms were anesthetized in 5 mM Levamisole and mounted in M9 on a 2% agarose pad. 60 x 0.5 μm z-stacks were acquired using a deconvolution microscope (Deltavision Elite; Applied Precision) equipped with a CMOS camera (pco.edge 5.5 sCMOS; PCO) and a 60X 1.42NA PlanApo N objective (Olympus). The z-stacks were deconvolved and maximum intensity projections were made using softWorRx (Applied Precision).

To image the ASER neuron expressing mNeonGreen and mKate-SNB-1 markers, synchronized adult worms were anesthetized as described above. 60 x 0.5 μm z-stacks were acquired using an inverted Zeiss Axio Observer Z1 system with a Yokogawa spinning-disk confocal head (CSU-X1), a 63X 1.40 NA Plan Apochromat objective (Zeiss, Oberkochen, Germany), and an EMCCD camera (QuantEM:512SC, Photometrics, Tucson, AZ). Maximum intensity projections of z-stacks were made using Image J (Fiji).

Assay for Egg-Laying Defect

The number of unlaid eggs was determined as described previously (Koelle and Horvitz, 1996). Briefly, late stage L4s were collected and grown at 20°C for 36 hours. Each adult worm was bleached in 2 μl 50% sodium hypochlorite solution to dissolve the mother. Subsequently, the embryos, which are protected from the bleach by their eggshell, were counted.

Fertility Assay

L4 worms were singled onto NGM plates and transferred to a fresh plate two additional times, every 24 hours. The progeny on each plate was counted 24 hours after the transfer and summed to give the brood size.

Dye-Fill Assay

Dye-fill assays were carried out by incubating 100 worms in a 1/500 dilution of DiI (Invitrogen) in M9 buffer for 1 hr. The worms were washed with M9, transferred on to a seeded NGM plate for 30 min to destain, anesthetized in 5mM Levamisole and mounted on a 2% agarose pad as described above. 60 x 0.5 μm z-stacks were acquired on the Andor system (described above) with a 40X 0.75 NA Plan Fluor objective (Nikon). Maximum intensity projections of z-stacks were made using Image J (Fiji).

QUANTIFICATION AND STATISTICAL ANALYSIS

Details of the methods employed to extract and quantify various parameters in microscopy datasets are described in the image analysis section. The statistical tests used to determine significance are described in the figure legends. Pairwise comparisons were done using 2-tailed unpaired t-tests in GraphPad Prism (GraphPad Software) and the stars ****, ***, ** and ns correspond $p<0.0001$, $p<0.001$, $p<0.01$ and "not significant", respectively.

Developmental Cell, Volume *48*

Supplemental Information

The Kinetochore-Microtubule Coupling Machinery Is Repurposed in Sensory Nervous System Morphogenesis

Dhanya K. Cheerambathur, Bram Prevo, Tiffany-Lynn Chow, Neil Hattersley, Shaohe Wang, Zhiling Zhao, Taekyung Kim, Adina Gerson-Gurwitz, Karen Oegema, Rebecca Green, and Arshad Desai

Figure. S1

Cheerambathur, Prevo et al.

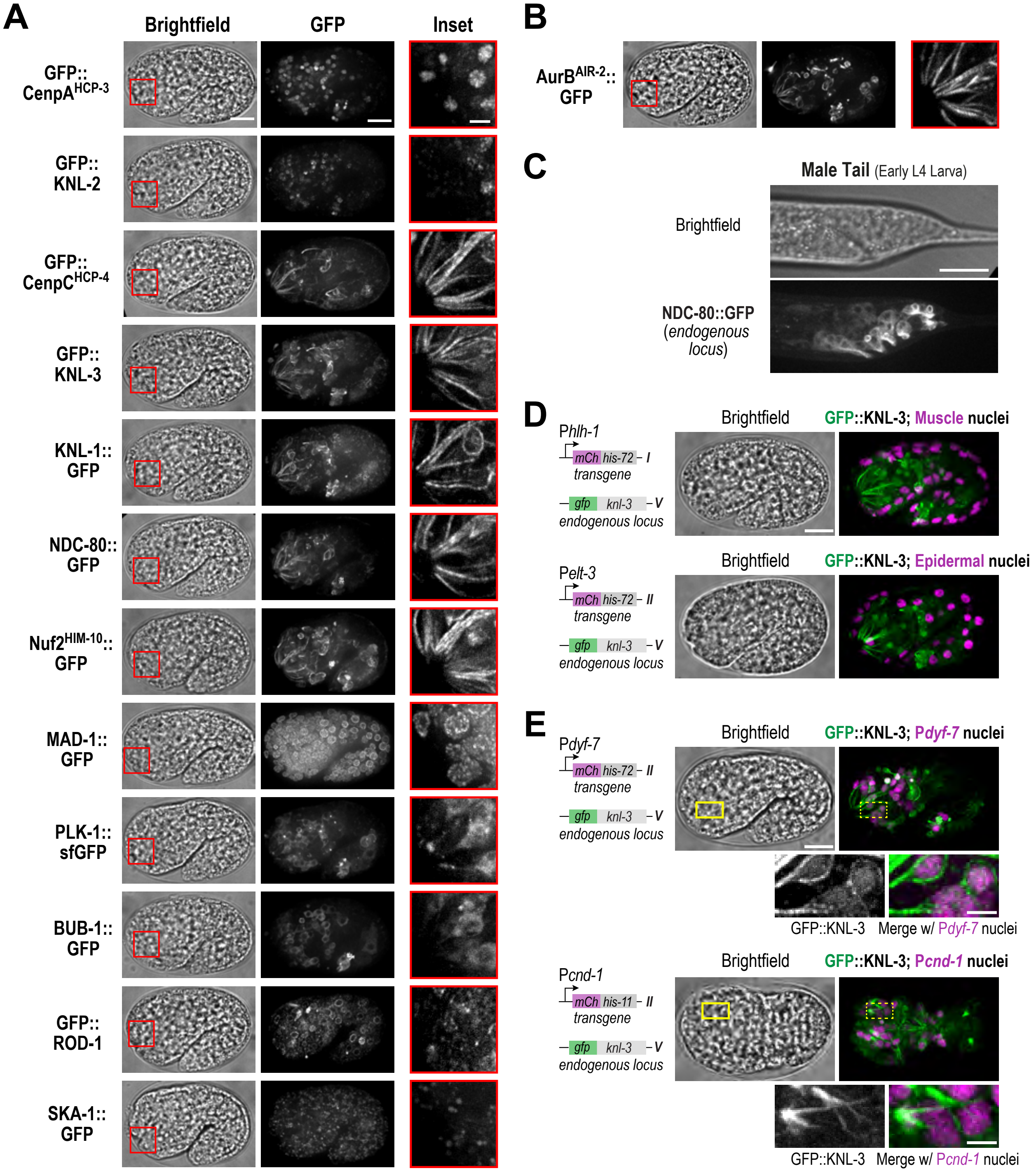

Figure. S1. Localization of kinetochore components and Aurora B^{AIR-2}, NDC-80 localization in the larval male tail, and KNL-3 localization in embryos expressing muscle, epidermal and neuronal nuclear markers (Related to Figure 1). (A) Images of full embryos and their anterior developing head regions (red box in brightfield panels) for the indicated *in situ* GFP fusions. Insets are the same as those shown in Figure 1B. Scale bars 10 µm in full embryos; 2.5 µm in inset. **(B)** Similar scale images as (A) for *in situ* GFP-tagged Aurora B^{AIR-2} kinase. **(C)** NDC-80::GFP localization in the male tail (early L4 larval stage). NDC-80 concentrates in the neurites of post-embryonic developing sensory neurons. Scale bar, 20 µm. **(D)** GFP::KNL-3 localization in embryos with single copy transgene insertions expressing nuclear-localized mCherry-tagged histone H3.3 (HIS-72) under control of muscle-specific (*hlh-1)* or epidermis-specific (*elt-3)* promoters (Krause et al.,1990; Gilleard et al.,1999) Scale bar 10 µm. **(E)** GFP::KNL-3 localization in embryos with single copy transgene insertions expressing nuclear-localized mCherry-tagged histone H3.3 (HIS-72) under control of the *dyf-7* promoter or histone H2B (HIS-11) under control of the *cnd-1* promoter. Region highlighted with yellow boxes is magnified below. The promoters P*dyf-7* and P*cnd-1* are transiently active in developing neurons. P*dyf-7* is activated at the time of early morphogenesis (Heiman et al., 2009) and P*cnd-1* is activated early in development (Hallam et al., 2000). The embryo stage shown is soon after morphogenesis initiates(see also Figure S2A). Scale bars: 10 µm for full embryo and 2.5 µm for insets.

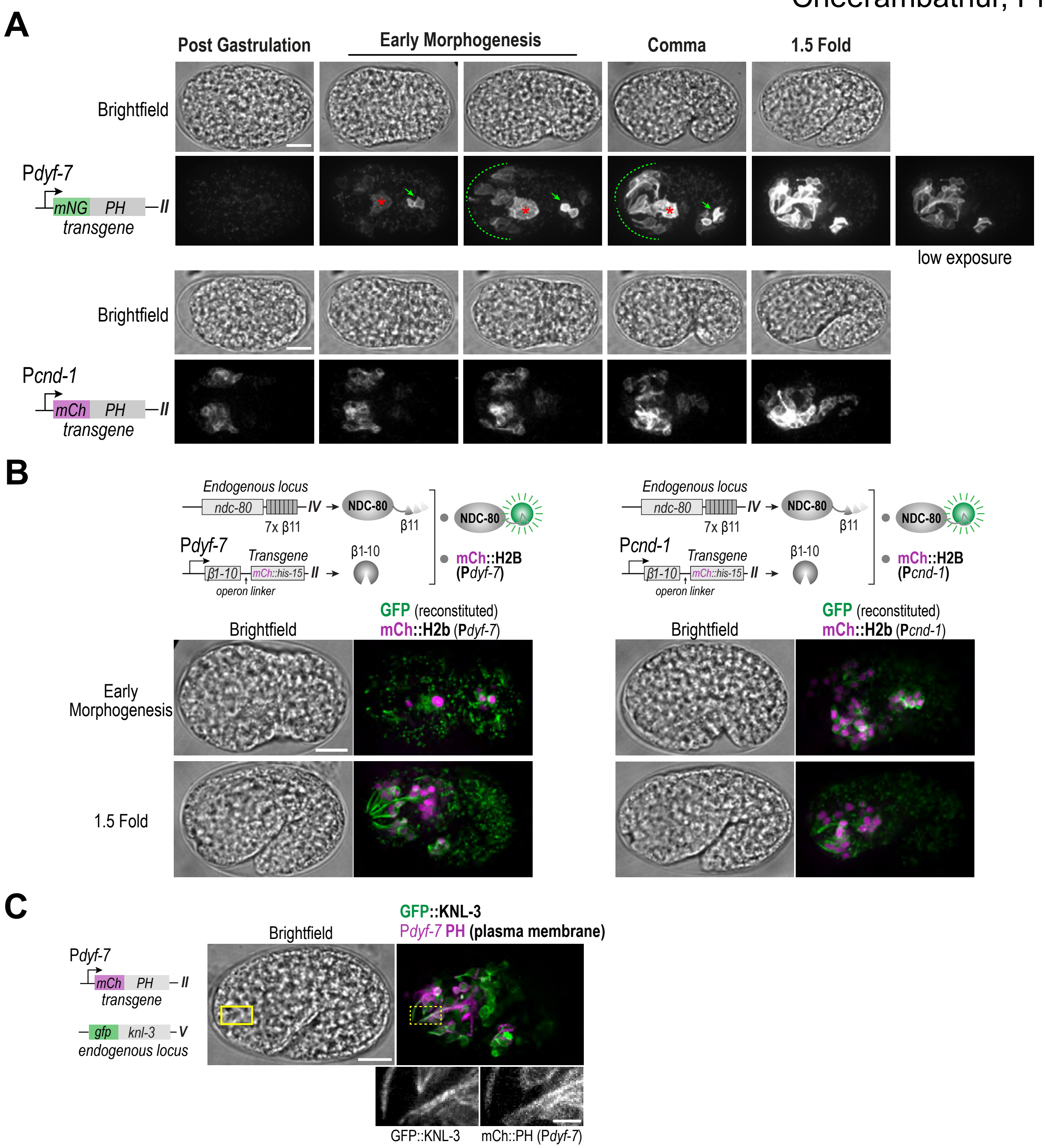

Figure. S2. Temporal and spatial profile of P*dyf-7* and P*cnd-1* activation, overlap of NDC-80 with P*dyf-7* and P*cnd-1* activation (Related to Figure 1).

(A) Images of fluorescent plasma membrane marker (the pleckstrin homology (PH) domain of mammalian Plc1δ1) expressed under control of P*dyf-7* or P*cnd-1* from a single copy transgene insertion. Localization similar to KMN proteins is highlighted (arrow & dashed line); red asterisk marks the excretory cell. Scale bar 10 µm. **(B)** Split GFP analysis using P*dyf-7* or P*cnd-1* to control expression of β1-10 of GFP in embryos where the *ndc-80* locus was engineered to fuse 7 copies of β11 to the NDC-80 C-terminus. The 1.5-fold stage embryo shown for P*dyf-7* is the same as in Figure 1E. Scale bar, 10 µm. **(C)** Image of 1.5-fold embryo with *in situ* GFP-tagged KNL-3 and P*dyf-7* controlled mCherry::PH plasma membrane marker. Region highlighted with yellow boxes is magnified below. mCherry maturation in embryos was slow and it was prone to aggregation, which greatly limited its utility for co-localization analysis. Scale bars: 10 µm for full embryo and 2.5 µm for insets.

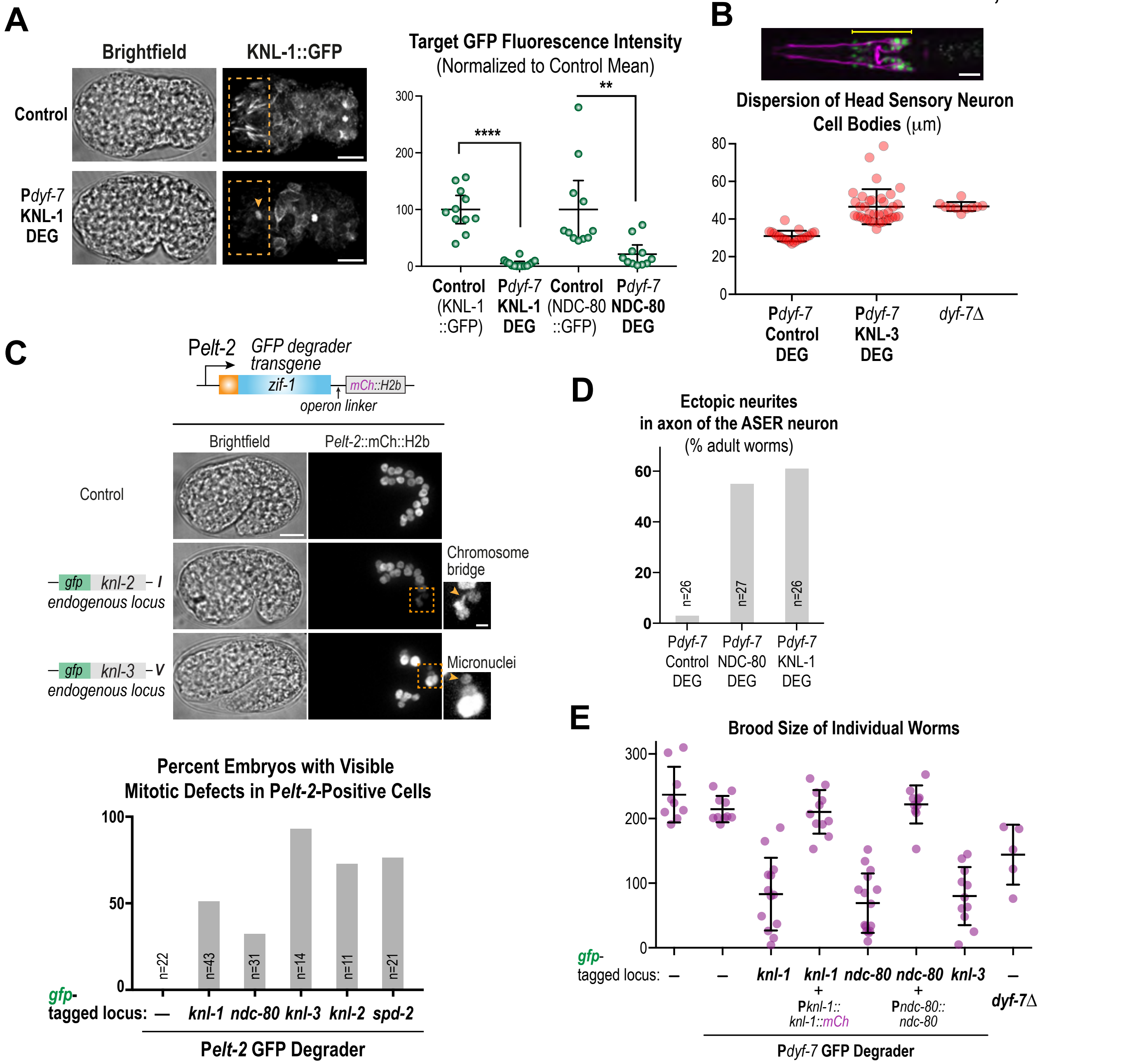

Figure. S3. GFP degrader analysis with P*dyf-7* and the intestinal promoter P*elt-2*, and phenotypes associated with P*dyf-7* controlled KMN degradation (Related to Figure 2). **(A)** Images and quantification of total GFP signal in the embryo anterior, measured during the early morphogenesis phase in embryos. The GFP signal in the embryo anterior (orange boxes) was quantified by integrating the fluorescence intensity above a threshold of 10000 a.u., while excluding GFP signal arising from kinetochore localization (arrowhead). Whole embryo images are for the anterior panels shown in Figure 2A. Scale bar, 10 µm. **(B)** Effect of KNL-3 degradation by the P*dyf-7* degrader or *dyf-7*Δ on sensory nervous system structure. The control data plotted is the same as in Figure 2C. **(C)** Mitotic defects following degradation of *in situ* GFP-tagged KMN, KNL-2 and SPD-2 in dividing cells during intestinal development, using a P*elt-2* controlled GFP degrader. P*elt-2* is activated in early intestinal development; the transgene includes a red fluorescent histone (separated from the GFP degrader by an operon linker) to mark the nuclear DNA of the cells in which the P*elt-2* promoter is active (Wang et al., 2017). Visible chromatin bridges and micronuclei (magnified on the right) in the mCh::H2b channel were scored as mitotic defects. The percentage of embryos with visible mitotic defects for the different targets of the P*elt-2* GFP degrader are plotted below. As not all mitotic defects result in visible bridges or micronuclei, the measured percentage is likely an underestimate. Scale bars: 10 µm (primary panels); 2.5 µm (magnified insets). **(D)** Quantification of ectopic neurite frequency in the ASER neuron for the indicated conditions. **(E)** Brood size of individual worms for the indicated conditions.

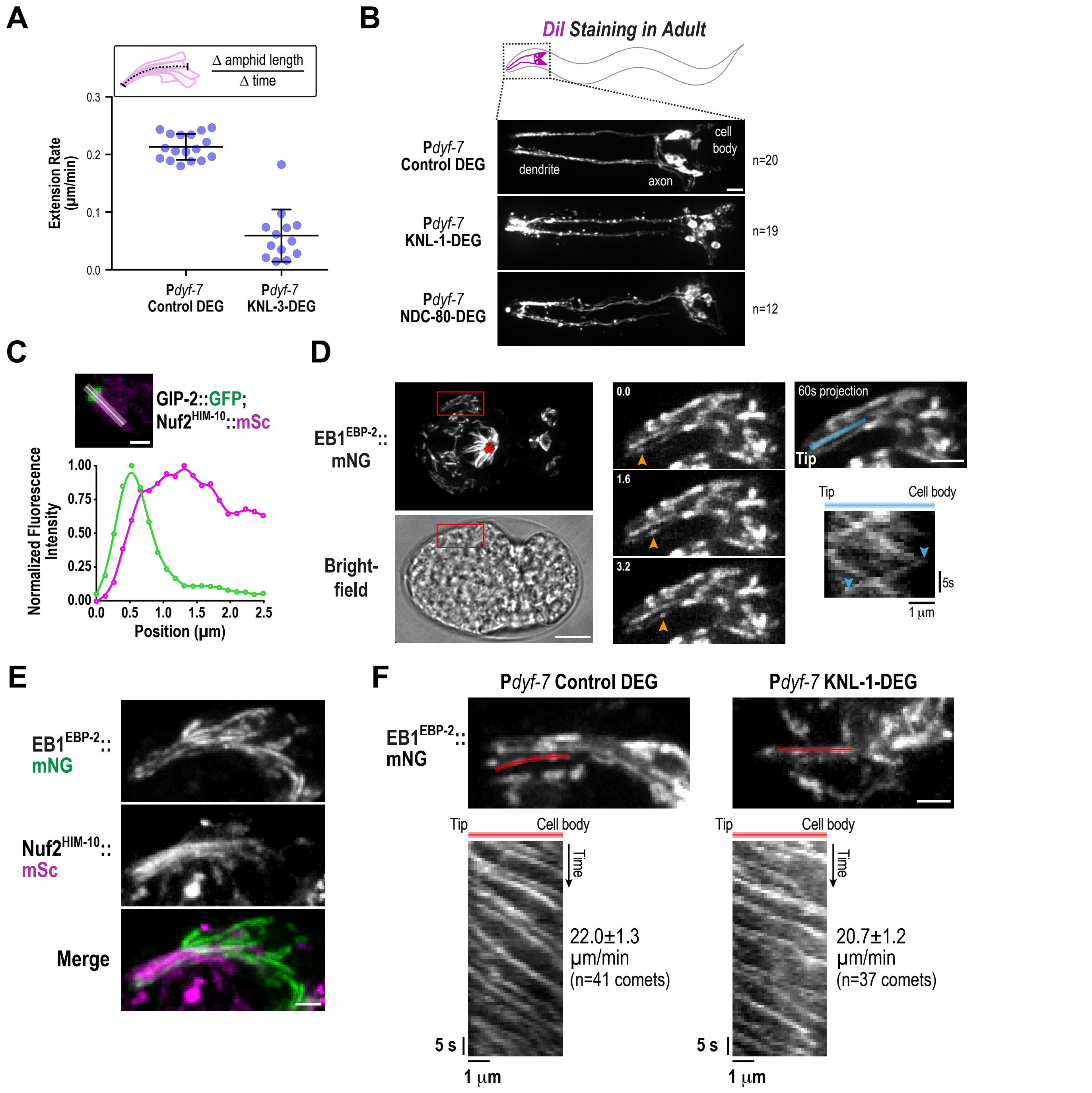

Figure. S4. Analysis of amphid dendrites: role of KNL-3, dye-filling and microtubule dynamics (Related to Figure 3). **(A)** Dendrite extension rate following P*dyf-7* controlled KNL-3 degradation. Control data plotted is the same as in Figure 3C. **(B)** Dye-fill assay in adult worms for the indicated conditions. Following P*dyf-7* controlled degradation of KNL-1 and NDC-80, the amphid neurons were able to dye-fill indicating they have assembled cilia. Scale bar, 10 μm. **(C)** 3 pixel-wide linescan profile along dendrite of lower inset. Scale bar,1 μm (ii) in Figure 3D. Scale bar, 1 μm. **(D)** $EB1^{EBP-2}$ dynamics in amphid bundles. Whole embryo images highlight the amphid bundle magnified in Figure 3E and to the right. Asterisk marks the excretory cell, in which P*dyf-7* is active but KMN proteins are not expressed. Middle column shows fast timelapse images (time is in seconds); arrows point to a microtubule plus end comet. The kymograph for this dendrite is shown in Figure 3E. Column on the right shows a kymograph with plus end trajectories in both directions, indicating mixed polarity. The image for the 60s projection is the same as in Figure 3E. Scale bar for whole embryo images: 10 μm; for amphid bundles: 2.5 μm. **(E)** 2-color localization analysis of $EB1^{EBP-2}$ and *in situ* mScarlet-tagged $Nuf2^{HIM-10}$. Scale bar, 2 μm. **(F)** $EB1^{EBP-2}$ dynamics in P*dyf-7* Control DEG and P*dyf-7* KNL-1 DEG amphid bundles. Scale bar, 2 μm. Red lines indicate location of kymographs depicted below. Mean and 95% CI of growth velocity measure-mements is shown to the right of each kymograph.

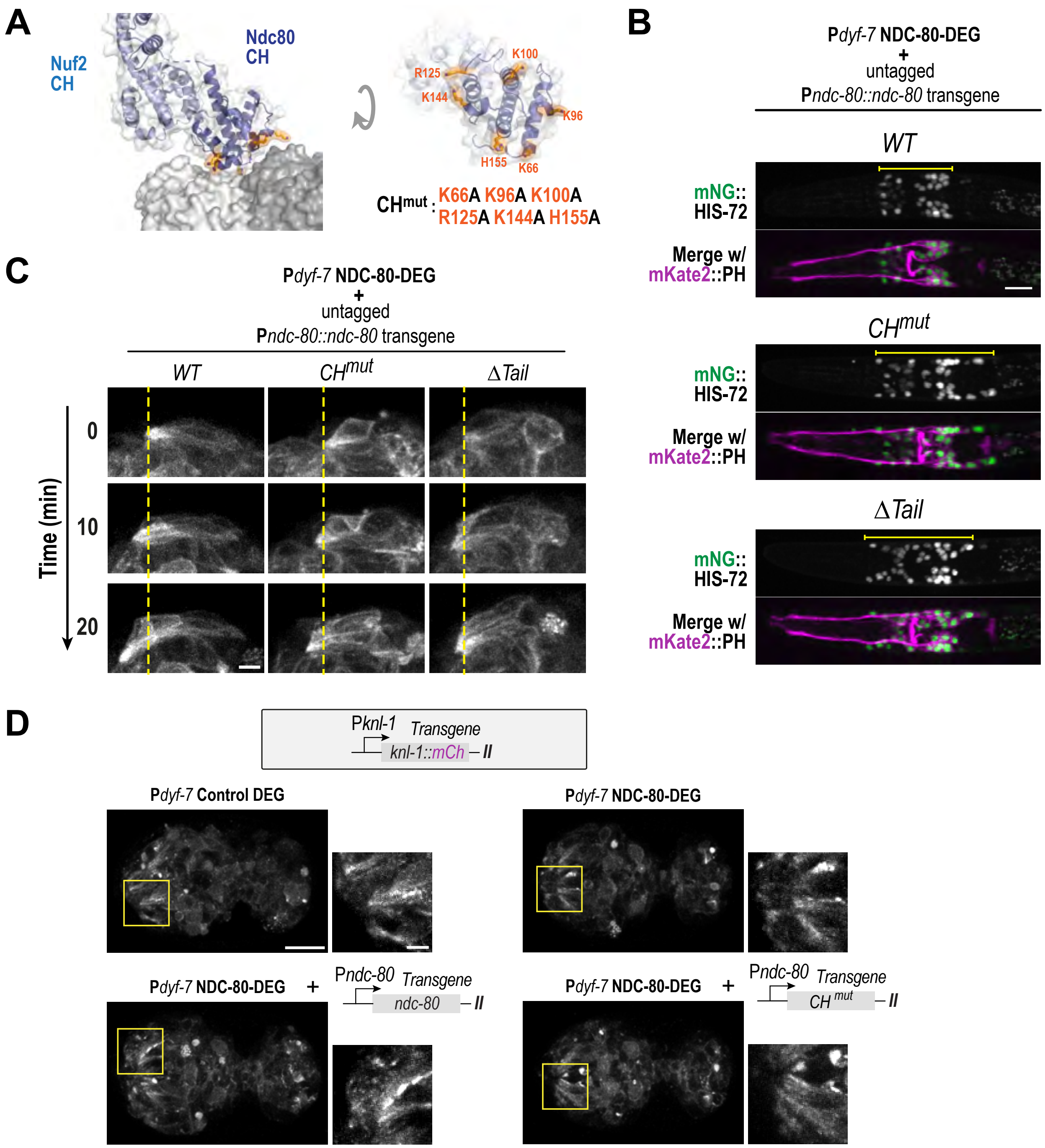

Figure. S5. Analysis of NDC-80 complex microtubule binding mutants (Related to Figure 5). **(A)** Structure of human Ndc80 complex bound to the microtubule surface (PDB: 3IZ0). Residues of the Ndc80 CH domain critical for microtubule binding are indicated on the rotated, *en face* view on the right. Amino acid numbers are for *C. elegans* NDC-80. **(B)** Sensory nervous system architecture in the L1 larval head visualized as in Figure. 2B. Scale bar, 10 µm. **(C)** Amphid bundle dendrite extension analysis. The WT images as the same as in Figure. 3B. Scale bar, 2.5 µm. **(D)** KNL-1::mCherry localization. Scale bar, 10 µm (embryo); 2.5 µm (inset).

References:

Gilleard, J.S., Shafi, Y., Barry, J.D., McGhee, J.D., 1999. ELT-3: A Caenorhabditis elegans GATA factor expressed in the embryonic epidermis during morphogenesis. Dev. Biol. 208, 265–280. doi:10.1006/dbio.1999.9202

Hallam, S., Singer, E., Waring, D., Jin, Y., 2000. The C. elegans NeuroD homolog cnd-1 functions in multiple aspects of motor neuron fate specification. Development 127, 4239–4252.

Heiman, M.G., Shaham, S., 2009. DEX-1 and DYF-7 establish sensory dendrite length by anchoring dendritic tips during cell migration. Cell 137, 344–355. doi:10.1016/j.cell.2009.01.057

Krause, M., Fire, A., Harrison, S.W., Priess, J., Weintraub, H., 1990. CeMyoD accumulation defines the body wall muscle cell fate during C. elegans embryogenesis. Cell 63, 907–919.

Table S1: CRISPR-Cas9 Protocol & sgRNA Sequences Used for Strain Generation (Related to STAR Methods)

STRAIN NAME	GENOTYPE	METHOD & REFERENCE	sgRNA SEQUENCE
OD2953	*him-10(lt52[him-10::GFP])III*	Direct Integration (Waaijers et al., 2013)	ACGGAAAAACTCGAATCGTT
OD3026	*knl-1(lt53[knl-1::GFP::tev::loxP::3xFlag])III*	SEC (Dickinson et al., 2015)	TCGAATGCTGGTGTTCTCTA
OD3029	*ndc-80(lt54[ndc-80::GFP::tev::loxP::3xFlag])IV*	SEC (Dickinson et al., 2015)	ATGTGCTGGCATTGAAAAGG
OD3101	*knl-3(lt46 [GFP::knl-3])V*	Direct Integration (Waaijers et al., 2013)	CGCGGCTCTGACCGAGAATG
OD3230	*air-2(lt58[air-2::GFP::tev::loxP::3xFlag])I*	SEC (Dickinson et al., 2015)	TTTTTGCCTCCATCATTCCC, GGAAGGAACACTACTGGATCC
OD3244	*dyf-7(lt60)X*	Ribonucleoprotein complex (Paix, et al., 2015)	CTTCAAGTATGAATCAATTG, TTTGGCAGGTGTCCAGTGAA
OD3367	*rod-1(lt62[GFP::rod-1)IV*	Direct Integration (Waaijers et al., 2013)	CCACAGCTTTTGCTTCGCCT
OD3407	*knl-2(lt73[GFP::knl-2])I*	Direct Integration (Waaijers et al., 2013)	CATCTACTAATCTCTGTGCA
OD3453	*spd-2(lt76[gfp::spd-2]) I*	Direct Integration (Waaijers et al., 2013)	TATTCTCAGCGTATTAAAA, TGTTCATTACAGAGATTCAT
OD3410	*hcp-4(lt72[GFP::hcp-4])I*	Direct Integration (Waaijers et al., 2013)	ACAATCGTACTGCGGGTTCG
OD3463	*hcp-3(lt78[GFP::hcp-3])I*	Direct Integration (Waaijers et al., 2013)	CGATGACACCCCAATTATTG
OD3516	*bub-1(lt82 [bub-1::GFP])I*	Direct Integration (Waaijers et al., 2013)	TCATTGTGTTGGGCTACTTT, TTGGTTGGCGGCAAGATCAC
OD3995	*ndc-80[lt126 (ndc-80::7Xβ-11)] IV*	Direct Integration (Waaijers et al., 2013)	ATGTGCTGGCATTGAAAAGG
OD4040	*him-10[lt130(him-10::mSca)] III*	Direct Integration (Waaijers et al., 2013)	ACGGAAAAACTCGAATCGTT

Table S2: Sequences of Regulatory elements & Fluorescent Probes (Related to STAR Methods)

Regulatory element	Length	5' end	3'end
Pelt-2	2912 bp	5'-tacatctttaccggcaccagaaga-3'	5'-agaaactagaaaatagattataga-3'
Pelt-3	2507 bp	5'-cacgttgtttcacggtcatcgtcg-3'	5'-tatcgagtggaaaaagtggccaac-3'
Phlh-1	3345 bp	5'-tgggttaatgtaggtgctggaagg-3'	5'-aattttccagaaatgaacacggaa-3'
Pcnd-1	3230 bp	5'-cagctatgacacgtggctctagta-3'	5'-tgtcatccagttatattttctaca-3'
Pdyf-7	3324 bp	5'-ttcatatactttatgtacggcgta-3'	5'-ctatttcagatttaaacttcaagt-3'
Pnphp-4	874 bp	5'-aaatcagggaaagtacatttttga-3'	5'-tttgtggtaacaaagtctcgaaaa-3'
Pgcy-5	3453 bp	5'-gcggtcaactagtgtatgattcct-3'	5'-aaaaattacttattctgatgaaaa-3'
unc-54 3'UTR	699 bp	5'-gtccaattactcttcaacatccct-3'	5'-ccaatataccaaacataactgttt-3'
dyf-7 3'UTR	1203 bp	5'-aataccgccattcacctcttatttt-3'	5'-cttggtctttcttttgttttagaa-3'
tbb-2 3'UTR	330 bp	5'-atgcaagatcctttcaagcattcc-3'	5'-gccgccaagaaaaaagtctcattg-3'
snb-1 3'UTR	663 bp	5'-gtacacgacctttgtcccggataa-3'	5'-ttagacggcacaataagccaccgg-3'

Sequences of Fluorescent Probes

Probe	Sequence
mScarlet	ATGGTCAGCAAGGGAGAGGCAGTTATCAAGGAGTTCATGCGTT<br>TCAAGGTCCACATGGAGGGATCCATGAACGGACACGAGTTCGA<br>GATCGAGGGAGAGGGAGAGGGACGTCCATACGAGGGAACCCA<br>AACCGCCAAGCTCAAGGTCACCAAGgtaagtttaaacatatatatactaact<br>aaccctgattatttaaattttcagGGAGGACCACTCCCATTCTCCTGGGACA<br>TCCTCTCCCCACAATTCATGTACGGATCCCGTGCCTTCACCAAGC<br>ACCCAGCCGACATCCCAGACTACTACAAGCAATCCTTCCCAGAG<br>GGATTCAAGTGGGAGCGTGTCATGAACTTCGAGGACGGAGGAG<br>CCGTCACCGTCACCCAAGgtaagtttaaacagttcggtactaactaaccatacat<br>atttaaattttcagACACCTCCCTCGAGGACGGAACCCTCATCTACAAG<br>GTCAAGCTCCGTGGAACCAACTTCCCACCAGACGGACCAGTCAT<br>GCAAAAGAAGACCATGGGATGGGAGGCCTCCACCGAGCGTCTC<br>TACCCAGAGGACGGAGTCCTCAAGGGAGACATCAAGATGGCCC<br>TCCGTCTCAAGGACGGAGGACGTTACCTCGCCGACTTCAAGgtaa<br>gtttaaacatgattttactaactaactaatctgatttaaattttcagACCACCTACAAGG<br>CCAAGAAGCCAGTCCAAATGCCAGGAGCCTACAACGTCGACCG<br>TAAGCTCGACATCACCTCCCACAACGAGGACTACACCGTCGTCG<br>AGCAATACGAGCGTTCCGAGGGACGTCACTCCACCGGAGGAAT<br>GGACGAGCTCTACAAG

splitGFP β1-10	ATGAGTAAAGGAGAAGAACTTTTCACTGGAGTTGTCCCAATTCTT<br>GTTGAATTAGATGGTGATGTTAATGGGCACAAATTTTCTGTCCGT<br>GGAGAGGGTGAAGGTGATGCAACAATCGGAAAACTTACCCTTA<br>AATTTATTTGCACTACTGGAAAACTACCTGTTCCATGGgtaagtttaa<br>acatatatatactaactaaccctgattatttaaattttcagCCAACACTTGTCACTAC<br>TCTTACCTACGGTGTTCAATGCTTCTCTAGATACCCAGATCACAT<br>GAAACGTCATGACTTTTTCAAGAGTGCCATGCCCGAAGGTTATG<br>TACAGGAAAGAACTATATCTTTCAAAGATGACGGGAAGTACAAG<br>ACACgtaagtttaaacagttcggtactaactaaccatacatatttaaattttcagGTGCT<br>GTCGTCAAGTTTGAAGGTGATACCCTTGTTAATAGAATCGAGTTA<br>AAAGGTACTGATTTTAAAGAAGATGGAAACATTCTTGGACACAA<br>ATTGGAATACAACTTCAACTCACACAATGTATACATCACCGCAGA<br>CAAACAAAAGAATGGAATCAAAGCTgtaagtttaaacatgattttactaacta<br>actaatctaatttaaattttcagAACTTCACCGTCAGACACAACGTCGAAGA<br>TGGAAGCGTTCAACTAGCAGACCATTATCAACAAAATACTCCAAT<br>TGGCGATGGCCCTGTCCTTTTACCAGACAACCATTACCTGTCCAC<br>ACAAACCGTCCTTTCGAAAGATCCCAACGAAAAG
splitGFP 7Xβ11	GGAGGGAGGGCCGGCTCTGGAGGCTCTGGAAGAGATCATATG<br>GTCCTCCACGAATACGTCAACGCTGCCGGGATCACTGGAGGAT<br>CTGGAGGACGTGACCATATGGTCCTCCACGAATACGTCAATGCC<br>GCCGGAATCACCGGAGGTTCCGGAGGACGTGATCACATGGTCC<br>TCCACGAATACGTCAACGCTGCCGGGATCACTGGAGGAAGCGG<br>AGGACGCGATCATATGGTCCTCCACGAGTACGTTAACGCCGCTG<br>GAATCACCGGAGGATCCGGAGGTAGAGACCATATGGTCCTTCA<br>CGAATACGTCAACGCCGCTGGAATCACCGGTGGATCCGGTGGA<br>CGTGATCACATGGTTCTTCATGAGTACGTTAACGCTGCTGGAAT<br>CACC
